# Determination of complete chromosomal haplotypes by bulk DNA sequencing

**DOI:** 10.1186/s13059-021-02330-1

**Published:** 2021-05-06

**Authors:** Richard W. Tourdot, Gregory J. Brunette, Ricardo A. Pinto, Cheng-Zhong Zhang

**Affiliations:** 1 Department of Data Science, Dana-Farber Cancer Institute, 3 Blackfan Circle, Boston, 02215 USA; 2 Department of Biomedical Informatics, Blavatnik Institute, Harvard Medical School, 10 Shattuck Street, Boston, 02115 USA; 3 Cancer Program, Broad Institute of MIT and Harvard, 415 Main Street, Cambridge, 02142 USA

**Keywords:** Haplotype, Chromosome rearrangement, Cancer genomics

## Abstract

Haplotype phase represents the collective genetic variation between homologous chromosomes and is an essential feature of non-haploid genomes. Here we describe a computational strategy to reliably determine complete whole-chromosome haplotypes using a combination of bulk long-range sequencing and Hi-C sequencing. We demonstrate that this strategy can resolve the haplotypes of parental chromosomes in diploid human genomes with high precision (>99%) and completeness (>98%) and assemble the syntenic structure of rearranged chromosomes in aneuploid cancer genomes at base pair level resolution. Our work enables direct interrogation of chromosome-specific alterations and chromatin reorganization using bulk DNA sequencing.

**Supplementary Information:**

The online version contains supplementary material available at (10.1186/s13059-021-02330-1).

## Background

Haplotype (“haploid genotype”) phase is the combination of genotypes at sites of genetic variation along a chromosome [1]. Haplotype information is required for performing diploid genome assembly [2, 3], interrogating differences in the DNA sequence or epigenetic features between homologous chromosomes [4–6], and relating them to allele-specific gene expression variation [7–9]. Haplotype information can also significantly improve the precision of somatic mutation analysis in polyclonal populations [10, 11] or single cells [12].

There are two strategies of haplotype inference [6, 13]. The first strategy (“statistical phasing”) [14–16] infers haplotype phase based on the recombination probabilities between variant genotypes estimated from linkage disequilibrium in a population [17, 18]. Although statistical phasing can infer haplotype linkage between adjacent variant sites at reasonably high accuracy (>99%), it cannot extend haplotype blocks beyond 10Mb due to accumulation of random switching errors, except with knowledge of the genotypes of closely related individuals [19, 20]. Statistical phasing is also limited to common polymorphisms and not applicable to de novo mutations.

The second strategy directly extracts haplotype linkage from the sequences of single chromosomes or sub-haploid chromosomal fragments (“molecular linkage”) [6]. Direct sequencing of single chromosomes can produce whole-chromosome haplotypes [21–25] but is only applicable to dividing cells and requires laborious experimental procedures of chromosome isolation or tagging. Long-read sequencing or long-range sequencing can either reveal haplotype linkage directly from long contiguous reads, or indirectly from short DNA fragments derived from long DNA molecules that are tagged with unique molecular barcodes [26–34]. The typical size of DNA molecules in long-read or long-range sequencing (10–100kb) is sufficient to link variants in regions of normal variant density (∼1 per kb), but inadequate in regions of low variant density (<1 per 10 kb) and unable to bridge large gaps (>100kb) with no identifiable variants, including all centromeres.

Intra-chromosomal (*cis*) linkage information is also contained in Hi-C fragments generated by proximity-based chromatin ligation [35]. As chromosomes are spatially isolated in separate territories in the cell nucleus, Hi-C contacts are predominantly formed within a single chromosome and can reveal *cis* linkage across the entire chromosome [36] without single-chromosome isolation. However, long-range Hi-C contacts are very sparse and only a small fraction of them overlap with sites of genetic variation except for genomes with very high variant density (∼1 per 150 bp) [36]. The sparsity of haplotype linkage from Hi-C data limits its power to generate contiguous haplotypes [37] or accurately phase de novo mutations.

**Fig. 1:**
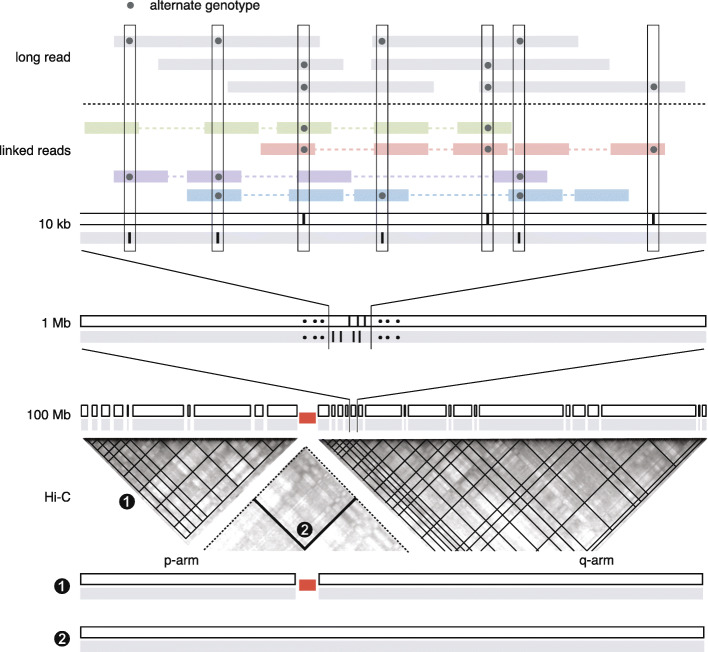
A hierarchical strategy for determining whole-chromosome haplotypes. The haplotype phase of parental chromosomes (open and filled rectangles) are represented by the positions of alternate genotypes at heterozygous sites. Megabase-scale haplotype blocks are determined using linkage information (∼10kb range) from linked-reads or long-read sequencing and then concatenated using Hi-C contacts, first within each chromosome arm and then between the p- and q-arms

Here we describe a computational strategy to accurately determine complete whole-chromosome haplotypes using a combination of long-range sequencing and Hi-C sequencing (Fig. 1). In contrast to previous methods that perform joint haplotype inference using linkage information from different technologies [37–39], we first determine high-confidence local haplotype blocks using linkage information from long-range/long-read sequencing and then merge these blocks into a single haplotype using Hi-C contacts. We formulate both local haplotype inference and haplotype block concatenation as a minimization problem that can be efficiently solved by steepest descent methods. Applying our approach to two diploid human samples with reference haplotype data, we demonstrate that the computational inference reproduces the haplotypes of parental chromosomes with high accuracy (>99%) and completeness (>98%). We further describe applications of haplotype-specific sequence coverage and Hi-C contact to resolving chromosomal alterations in aneuploid cancer genomes. We demonstrate the feasibility to generate haplotype-resolved karyotypes of aneuploid cancer genomes using bulk long-range and Hi-C sequencing by constructing a digital karyotype of the K-562 genome using published data.

## Results

### Data sources

We performed computational haplotype inference and benchmarking on two diploid genomes (data sources listed in Table 1). For the NA12878 genome, we used published linked-reads and Hi-C data for haplotype inference; for the retinal pigment epithelium-1 (RPE-1) genome, we used newly generated linked-reads data and published Hi-C data for haplotype inference. For benchmarking of the NA12878 haplotype solution, we used two public reference haplotype datasets. The first was released by the Genome-In-A-Bottle (GIAB) consortium; the second was generated from diploid de novo assembly of the NA12878 genome using PacBio High-Fidelity reads [40] in combination with short reads of the parental genomes. For benchmarking of the RPE-1 haplotype solution, we used the RPE-1 haplotypes determined directly from single-cell sequencing data of monosomic RPE-1 cells as reference. We further evaluated haplotype inference using low-pass (11 ×) PacBio circular-consensus sequencing (CCS) data of RPE-1 cells. For applications to aneuploid genomes, we used bulk whole-genome sequencing data of aneuploid RPE-1 cells from Ref. [41] and published cytogenetic [42, 43] and sequencing data of K-562 cells (data sources listed in Additional file 1:Table S1). See “Generation of sequencing data” and “Sequence data processing” subsections in the “Methods” section for more details of data generation and processing.

**Table 1:** Sources of data for parental haplotype inference and benchmarking

Sample	Data type	Data source	Read count	Mean	Contacts	Application
				depth	(>1Mb)	
RPE-1	Bulk WGS	[24]	228,708,769^a^	13 ×		Variant calling
RPE-1	Linked reads	New	941,518,426^b^	60 ×^c^		Variant calling
						& local phasing
RPE-1	CCS long reads	New	4,607,047^d^	11 ×		Local phasing
RPE-1	Hi-C	[44]	281,285,484^e^		48,124,211	Long-range phasing
RPE-1	Single cell with	New				hi-conf variants and
	monosomies					reference haplotypes
NA12878	Linked reads v.1	10X Genomics^f^	422,179,395^g^	35 ×^c^		Local phasing
NA12878	Linked reads v.2	10X Genomics^h^	423,854,243^i^	35 ×^c^		Local phasing
NA12878	Hi-C	[35]	486,848,169^j^		91,428,507	Long-range phasing
NA12878	Phased VCF	GIAB^k^				hi-conf variants and
						reference haplotypes
NA12878	Phased VCF	Diploid assembly^l^				hi-conf variants and
						reference haplotypes

^a^SRR1778442: median insert 243; 208,151,992 fragments aligned in pair; 2 ×101bp reads; duplication rate 0.024.

^b^Mean molecular length 24.8kb; median insert 551; 913,660,083 aligned in pair; 2 ×150bp reads; duplication rate 0.255.

^c^excluding the GEMcode sequence and duplicated fragments

^d^Mean read length 7.1kb; 4,606,654 aligned.

^e^SRS1045722: median insert 364; 279,027,892 aligned in pair; 2 ×150bp reads; duplication rate 0.067.

^f^https://support.10xgenomics.com/genome-exome/datasets/2.1.0/NA12878_WGS_210

^g^Mean molecular length 68.7kb; median insert 349; 407,015,530 aligned in pair; 2 ×150bp reads; duplication rate 0.062.

^h^https://support.10xgenomics.com/genome-exome/datasets/2.2.1/NA12878_WGS_v2

^i^Mean molecular length 85.6kb; median insert 370; 418,283,435 aligned in pair; 2 ×150bp reads; duplication rate 0.079.

^j^SRR1658572: median insert 377; 484,211,662 aligned in pair; 2 ×101bp reads; duplication rate 0.028.

^k^https://ftp-trace.ncbi.nlm.nih.gov/ReferenceSamples/giab/release/NA12878_HG001/latest/GRCh38/

^l^http://ftp.dfci.harvard.edu/pub/hli/hifiasm/NA12878-r253/. Phased variants were determined using dipcall (https://github.com/lh3/dipcall) on the sequences of parental chromosomes generated by diploid de novo assembly of the NA12878 genome using PacBio High-Fidelity long reads together with short reads of the parental genomes using hifiasm [40].

### Density and accuracy of molecular haplotype linkage

**Fig. 2:**
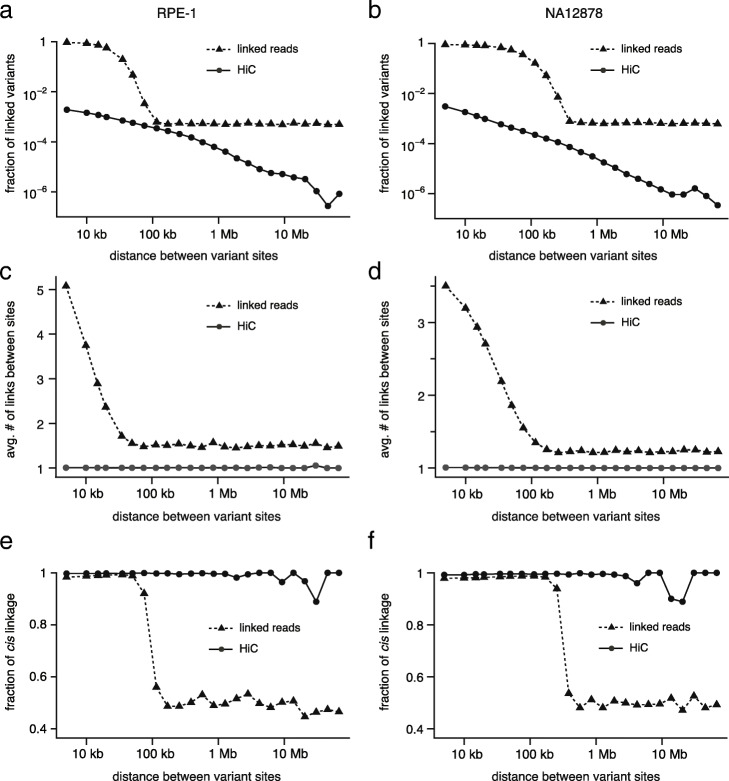
Statistical metrics of molecular haplotype linkage between variants at different genomic distance extracted from linked-reads and Hi-C sequencing data of RPE-1 (**a**,**c**,**e**) and NA12878 (**b**,**d**,**f**) cells. **a**, **b** Fraction of linked variants. **c**, **d** Average number of links between linked variants. **e**, **f** Fraction of links consistent with *cis* linkage. The residual linkage in the linked-reads data ∼50% accuracy (**e** and **f**) reflects tagging of unrelated DNA fragments from both parental homologs by the same molecular barcodes by chance

We first assessed the density and accuracy of molecular haplotype linkage from linked-reads and Hi-C sequencing to design the best strategy to integrate linkage evidence from both data types. The basic unit of linkage evidence (“molecular link”) is a DNA molecule, which can be a single sequencing read (long-read or Hi-C sequencing) or consist of multiple sequencing reads tagged with the same molecular barcode (“synthetic long read”). Molecular haplotype linkage is represented using variant genotypes in each DNA molecule (“Haplotype inference from linkage evidence” section). We extracted molecular linkage from the RPE-1 and NA12878 sequencing data (Additional file 1:Extracting variant linkage information from long-range sequencing) and calculated three metrics of haplotype linkage between variant sites at different genomic distance: (1) percentage of variants with molecular linkage; (2) average number of links between linked variants; and (3) percentage of links consistent with *cis* linkage according to the reference haplotype data. These results are shown in Fig. 2.

In both linked-reads and Hi-C sequencing data, the signal of molecular haplotype linkage is strongest between variants in close proximity but shows different decays against the genomic distance between variants. In the linked-reads data, the range of haplotype linkage is capped by the size of input DNA molecules. The maximum range of haplotype linkage is ∼100 kb in the RPE-1 data (Fig. 2a, c) and ∼300 kb in the NA12878 data (Fig. 2b, d). Both the density (Fig. 2a-d) and the accuracy (Fig. 2e, f) of haplotype linkage decays rapidly as the distance between variants exceeds the molecular size. The distance-independent linkage signal above the molecular size showing 50% *cis* and *trans* linkage (Fig. 2e, f) is consistent with random tagging of DNA fragments from both parental chromosomes. This residual signal most likely results from unrelated DNA molecules being tagged by the same molecular barcode and should be excluded from haplotype inference. The limited range of molecular linkage from linked-reads data (∼100kb) suggests that this datatype is suitable for local haplotype phasing but cannot extend haplotype blocks across regions with low variant density (<1 per 100kb).

The density of haplotype linkage from Hi-C data shows a power-law decay against genomic distance (Fig. 2a, b) that is similar to the frequency of intrachromosomal contacts [45, 46], suggesting that most Hi-C links result from intramolecular contacts (random intermolecular contacts will generate a distance-independent signal and cause deviation from the power-law decay). This is verified by the result that more than 90% of all Hi-C links are consistent with *cis* linkage (Fig. 2e, f). Although Hi-C linkage can extend to the entire chromosome, it is very sparse: In both Hi-C data (RPE-1 and NA12878), the probability that two variant sites separated by 100 kb are linked by Hi-C reads is less than 10^−3^ (Fig. 2a, b) and almost all linkage consists of only one link (Fig. 2c, d). The sparsity of Hi-C linkage limits the accuracy and completeness of haplotype inference [37].

**Fig. 3:**
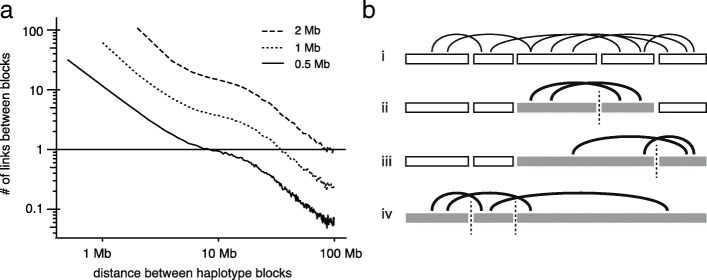
Linking haplotype blocks with Hi-C reads. **a** Average number of Hi-C links between two segments of 0.5Mb, 1Mb, and 2Mb at different genomic distance calculated using the RPE-1 Hi-C data. **b** A schematic illustration of haplotype block concatenation using Hi-C reads. The shown example assumes that at least two links are required to join two blocks. (i) Local haplotype blocks (open rectangles) and Hi-C links (curves); (ii) and (iii) merging of adjacent haplotype blocks based on Hi-C linkage; and (iv) joint inference of the haplotype phase of all blocks using all Hi-C links

One strategy to take advantage of long-range Hi-C linkage without significantly increasing the depth of sequencing is to aggregate Hi-C links between variants in local haplotype blocks to generate a stronger linkage signal. To demonstrate this quantitatively, we calculated the average number of Hi-C links between 0.5-, 1-, and 2-Mb segments at different genomic distance in the RPE-1 data (Fig. 3a). This calculation shows that the signal of haplotype linkage between megabase-scale segments can extend well above 10 Mb and is sufficient to link haplotype blocks across large gaps or regions of low variant density. As it is convenient to generate megabase-scale haplotype blocks either by statistical phasing or using long-range sequencing, the addition of Hi-C data with standard coverage (≥50 million long-range contacts) is sufficient to merge these blocks into a single haplotype for each chromosome (Fig. 3b). We have designed a general computational framework of haplotype inference based on molecular linkage evidence that is applicable to both local haplotype inference and haplotype block concatenation. This framework is presented in “Haplotype inference from linkage evidence” section with its implementation described in Additional file 1: Software implementation of the haplotype inference algorithm.

### Computational inference of parental haplotypes in diploid genomes

We applied our haplotype inference method to generate the complete haplotype phase of bi-allelic single-nucleotide variants (SNVs) in two diploid genomes (RPE-1 and NA12878) and benchmarked the computational inference against reference haplotype data. We detected heterozygous variants from the linked-reads data (“Variant calling and filtering” section) and extracted variant linkage in both linked-reads and Hi-C sequencing data (Additional file 1:Extracting variant linkage information from long-range sequencing). We excluded variants in centromeric or acrocentric regions due to the low variant detection accuracy in these regions caused by mis-alignment of short reads. We further omitted complex alterations, such as insertion, deletion, or structural variants, due to their lower detection and genotyping accuracy than SNVs from short reads.

We first performed local haplotype inference based on linkage evidence from the linked-reads data as described in Additional file 1:Solving haplotype phase by minimization. Only linkage between SNVs within 100kb was included in the calculation. The haplotype solution converged within 10 rounds of iterations for all chromosomes, with Chr.2 taking the longest time (1,000 seconds) to complete (Additional file 2). Our haplotype inference algorithm generated two scores measuring phasing accuracy at each variant site: The spin-flipping penalty (Eq. (18)) measures the probability of local (“short-switching”) phasing errors; the block-switching penalty (Eq. (19)) measures the probability of long-range switching errors. The distributions of these scores are shown in Additional file 1:Fig. S1.

**Fig. 4:**
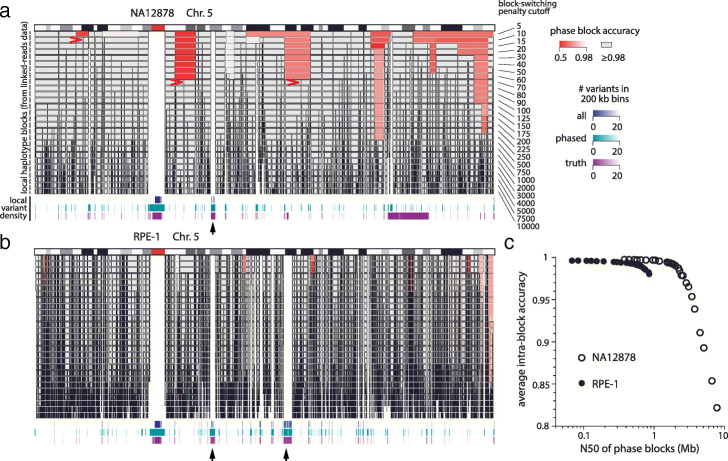
**a**, **b** Haplotype blocks on Chr.5 derived from the NA12878 (**a**) and RPE-1 (**b**) linked-reads data. Each row of haplotype blocks is determined using a different switching-penalty cutoff from *ΔE*=5 to *ΔE*=10,000. Only blocks with 50 or more phased variants are shown. The accuracy of each block is estimated by the percentage of genotypes that are consistent with the majority haplotype of each block determined using the reference haplotype. Blocks with ≥98% accuracy are colored in gray; those with <98% accuracy are colored in red with brightness scaled by the accuracy (minimum 50%). Three examples of low-accuracy blocks each containing a single intra-block switching error are highlighted with red arrows in the NA12878 genome; these blocks are broken into two high-accuracy blocks at a higher cutoff. Shown below the haplotype blocks are three tracks of regional variant density measured by the number of total detected variants (blue), phased variants in the final haplotype solution (green), and phased variants in the reference data (purple) in 200 kb bins. (We have chosen the Genome-In-A-Bottle data as the NA12878 reference haplotype.) Bins with more than 20 variants (variant density more than 1 per 10 kb) are omitted. Black arrows highlight large regions with low variant density, including the spinal muscular atrophy (SMA) region on 5q13.2 consisting of large (∼200kb) segmental duplications with high sequence similarity (>98%) [47] that cannot be resolved by short reads. The other region in the RPE-1 genome reflects loss-of-heterozygosity. **c**. Average intra-block accuracy (weighted by the number of variants in each block) and the N50 length of all haplotype blocks in each sample generated using different switching-penalty cutoffs. The NA12878 dataset produces longer haplotype blocks due to having longer input molecules (Table 1)

To demonstrate the utility of block-switching penalty scores for controlling long-range switching errors, we generated haplotype blocks using different block-switching penalty cutoffs (*ΔE*=5–10000) and assessed intra-block phasing accuracy using the reference haplotype. For each block, we first calculated the percentage of phased genotypes that agree with the reference haplotype *f* and then estimated intra-block accuracy as max(*f*,1−*f*). This definition corresponds to the fraction of genotypes consistent with the major haplotype assignment (min, 50%; max, 100%) and is very sensitive to long-range switching errors that cause a large fraction of genotypes to be assigned to the minor haplotype. The results for Chr.5 are shown in Fig. 4.

As expected, choosing lower block-switching cutoffs produces longer haplotype blocks with more intra-block switching errors than choosing higher cutoffs. Most low-accuracy blocks (colored in red) contain only one or a few switching errors at sites with low block-switching penalty scores: these blocks are broken to two or more high-confidence blocks at a higher block-switching cutoff (red arrows). Because intra-block switching errors will significantly compromise or destroy the signal of Hi-C linkage between blocks (“Phased Hi-C linkage between haplotype blocks” section), we elect to produce short haplotype blocks with high accuracy by choosing conservative block-switching cutoffs determined based on the location of the minimum in the block-switching penalty distribution (Additional file 1:Fig. S1B,E): *ΔE*=1000 for the RPE-1 data and *ΔE*=5000 for the NA12878 data. The resulting haplotype blocks are shorter than reported in Ref. [34] but have no apparent intra-block switching (<99% accuracy) (Fig. 4c). More discussion on the choice of block-switching penalty is given in “Assessing phasing accuracy and determining high-confidence haplotype blocks” section.

We note that sites prone to switching errors (having low block-switching penalty scores) are enriched in low-variant density regions (Additional file 1:Figs. S1C and S1F). Two large low-variant density regions on Chr.5 of the RPE-1 genome are highlighted in Fig. 4b (black arrows). The first one in 5q13.2 is also seen in the NA12878 genome. This region, known as the spinal muscular atrophy (SMA) region, contains large segmental duplications (∼200 kb) with high sequence similarity (>98%) [47] that cannot be resolved by short sequencing reads. Even though this region is not marked as having low variant-density based on unfiltered variants (blue tracks), the reference haplotype data show few phased variants in this region (purple tracks), suggesting a high fraction of false variants in the unfiltered callset. The exclusion of false or low-confidence variants from the haplotype solution (green tracks) confirms that our haplotype inference algorithm can effectively purge these variants based on the specificity of haplotype linkage. By contrast, the second low-variant density region in the RPE-1 genome near 5p21.1 contains few variants in the unfiltered callset and reflects true loss-of-heterozygosity. (See Additional file 1:Fig. S2 for a genome-wide map of low-variant density regions in the haplotype solution and in two independent reference datasets of the NA12878 genome. See Additional file 1:Fig. S3 and S4 for genome-wide maps of low-variant density regions in the NA12878 and RPE-1 genomes and local haplotype blocks generated from the linked-reads data using different switching penalty cutoffs.)

We merged high-confidence haplotype blocks using Hi-C links in two steps (Additional file 1:Concatenating haplotype blocks using Hi-C links). First, haplotype blocks within each chromosome arm were concatenated using Hi-C links between variants separated by ≤10Mb. Second, p- and q-arm haplotypes were joined using all Hi-C links between the arms. The consistency of haplotype solution in each step can be verified by comparing the number of *cis* and *trans* Hi-C links (Additional file 1:Fig. S5). We refer to the concatenated haplotype blocks as the “scaffold” haplotype solution.

Finally, we calculated the linkage between individual variant genotypes and phased variant genotypes in the scaffold haplotype solution using the number of unique molecules supporting each type of linkage (Additional file 1:Calculation of haplotype linkage between individual genotypes and the scaffold haplotype solution)

$$\begin{array}{*{20}l} \#(\text{reference-haplotype A}),\; \#(\text{alternate-haplotype B}), \\ \#(\text{reference-haplotype B}),\; \#(\text{alternate-haplotype A}). \end{array} $$

We determined the final haplotype phase at each variant site based on the combined linkage evidence defined as

1 $$\begin{array}{*{20}l} \eta_{\text{rA}}&=\#(\text{reference-haplotype A}) +\#(\text{alternate-haplotype B}); \end{array} $$

2 $$\begin{array}{*{20}l} \eta_{\text{rB}}&=\#(\text{reference-haplotype B}) + \#(\text{alternate-haplotype A}) \end{array} $$

and selected true heterozygous variants with haplotype linkage satisfying the following criteria. First, true heterozygosity requires that there is haplotype linkage to both genotypes (R and A) and both parental haplotypes (A and B). This was implemented as

$$\begin{array}{*{20}l} \text{(Ia)}\quad &\#(\text{reference-haplotype A})+\#(\text{reference-haplotype B})>0\\ \text{(Ib)}\quad &\#(\text{alternate-haplotype A})+\#(\text{alternate-haplotype B})>0\\ \text{(Ic)}\quad &\#(\text{reference-haplotype A})+\#(\text{alternate-haplotype A})>0\\ \text{(Id)}\quad &\#(\text{reference-haplotype B})+\#(\text{alternate-haplotype B})>0\\ \end{array} $$

Second, segregation of haplotype linkage between opposite genotypes (reference and alternate) and parental haplotypes (A and B) implies that max(*η*_rA_,*η*_rB_)≫ min(*η*_rA_,*η*_rB_)≈0. This was implemented as the following:

$$\text{(II)}\quad \min(\eta_{\text{rA}}, \eta_{\text{rB}})\leq \max[2,0.1\times(\eta_{\text{rA}}+\eta_{\text{rB}})].$$

(I) and (II) represent the “linkage filter” to exclude false variants in the final haplotype solution.

### Benchmark of the haplotype solution

We evaluated the accuracy and completeness of the computationally inferred haplotypes using the reference haplotype data determined directly from the sequence of parental chromosomes (Table 1). For the NA12878 genome, the reference haplotypes were determined using the parental genomes either by alignment-based analysis (the GIAB release) or by diploid de novo assembly of the NA12878 genome. Variants in both reference data have high specificity. The GIAB reference only includes high-confidence regions and leaves out several large regions including the p-arms of Chrs.16 and 18 (Additional file 1:Fig. S2). We used the haplotype derived from diploid de novo assembly to evaluate haplotype inference in these regions. For the RPE-1 genome, we determined the reference haplotypes from the sequencing data of monosomic RPE-1 cells (“Sequencing data of monosomic RPE-1 cells” section). As the RPE-1 variants were detected only from short-reads data, we filtered false variants based on the average variant allele fraction in the single-cell data.

We first benchmarked the scaffold haplotype solution constructed from large haplotype blocks (Additional file 1:Table S2 for NA12878 and Table S3 for RPE-1). We evaluated both the completeness of haplotype inference (percentage of variants in the reference data that are also phased in the computational solution) and the global accuracy of the haplotype solution (percentage of phased genotypes in agreement with the reference). The metric of global phasing accuracy is consistent with the metric of intra-block phasing accuracy defined above for local haplotype inference (Fig. 4).

For the NA12878 sample, the scaffold haplotype solution contains 1,746,304 out of 1,867,590 (93.5%) phased variants in the GIAB reference haplotype data and 2,037,593 out of 2,312,059 (88.1%) phased variants in the diploid-assembly reference and shows 99.6% agreement with both datasets. (Chromosome 19 has the lowest accuracy of 98.5%.) For the RPE-1 sample, the scaffold haplotype solution contains 2,071,147 out of 2,320,153 (89.3%) of all phased variants in the reference haplotype data and shows 98.3% agreement. (Chromosome 9 has the lowest percentage of agreement of 96.1%.) No chromosome in either sample shows <95% accuracy, suggesting that the combination of single-variant phasing errors and variants in switched blocks is less than 5%.

We then benchmarked the final haplotype solution determined using the linkage between variant genotypes and the scaffold haplotype solution (Eq. (1)). For the NA12878 sample, the final haplotype solution shows 99.7% accuracy and 97–98.0% completeness when compared to both reference data (Table 2; see Additional file 3 for detailed metrics for each chromosome). (Phased variants from de novo assembly but not detected in the linked-reads data were not included in the benchmark as these variants are not detectable by short reads.) The linkage filter removes 167,385 variants but does not affect phasing accuracy as most of the false variants are not present in the reference data. We further performed indel variant phasing on Chr.21 based on their molecular linkage to the scaffold haplotype phase of SNV genotypes (Additional file 1:Phasing of indel variants using haplotype linkage). The haplotype phase of indel variants shows similar accuracy when compared to the reference data, but the original callset (7663) contains significantly more variants than its intersection with either reference dataset (∼4000). The linkage filter removes a large number of variants in the unfiltered callset (most are likely false calls) and preserves 80–90% of phased variants in the reference data. This result demonstrates the utility of haplotype linkage for improving the specificity of variant detection that is independent of alignment accuracy.

**Table 2:** Comparison between the final haplotype solution and the reference haplotype of NA12878

All SNV sites	Phased from	Reference	Comparable	Agreed	Accuracy	Fraction of
	bulk data	haplotype	sites			completion
2,652,381	2,319,027^a^	1,861,941^b^	1,824,401	1,818,042	0.997	0.980
	2,151,642^c^		1,815,197	1,809,886	0.997	0.975
	2,319,027^a^	2,183,123^d^	2,122,256	2,114,548	0.996	0.969
	2,151,642^c^		2,096,982	2,091,821	0.998	0.958
**Indel variants**						
**on Chr.21**						
9,285	7663^e^	3618^f^	3553	3535	0.995	0.982
	4702^c^		3183	3177	0.998	0.880
	7663^e^	4581^g^	4478	4426	0.988	0.978
	4702^c^		3835	3827	0.998	0.837

^a^All phased variants without any filtering

^b^Variants detected in the linked-reads data that are also contained in the GIAB release. Total number of phased SNVs in the GIAB release, 1,867,590

^c^Filtered by haplotype linkage: ≥1 link connecting ref, alt, HapA, and HapB, and minor linkage ≤2 or minor linkage/total linkage ≤0.1

^d^Phased variants determined from phased de novo assembly of parental chromosomes that are also detected in the linked-reads data. Total number of phased variants from diploid de novo assembly, 2,312,059

^e^Variants phased by molecular linkage to phased SNVs in the scaffold haplotype solution

^f^Intersection with phased indel variants in the GIAB data with exactly matching variant genotypes. Total number of phased indels in the GIAB release, 4090

^g^Intersection with phased indels derived from de novo assembly of parental chromosomes with exactly matching variant genotypes. Total number of phased indels from diploid assembly, 7128

For the RPE-1 sample, the final haplotype solution shows 98% agreement with the reference data before variant filtration (Table 3). After excluding false variants based on either the variant allele fraction in the single-cell data (from >100 samples) or the specificity of haplotype linkage from linked reads, we see >99% agreement between the haplotype solution and the reference haplotype. The independent linkage filter and allele fraction filter show good consistency: 2,054,859 variants pass both filters and represent 95% of variants passing each individual filter. Among variants passing both filters, the percentage of agreement between the haplotype solution and the reference haplotype is 99.6% and comparable to the NA12878 haplotype solution. These results validate the completeness (>98%) and accuracy (>99%) of computational haplotype inference.

**Table 3:** Comparison between the final haplotype solution and the reference haplotype of RPE-1

Filter	Total variant	Phased from	Phased from	Agreed	Discordant	Fraction of
	sites	bulk data	monosomies			discordance
None	2,475,311	2,242,237	2,320,153	2,101,195	40,006	0.019
Allele fraction^a^	2,172,689	2,087,188	2,109,589	2,018,906	12,903	0.006
Linkage^b^	2,156,423	2,156,346	2,071,674	2,054,006	17,616	0.009
Combined^c^	2,054,859	2,054,832	2,001,674	1,993,552	8,098	0.004

^a^From single-cell data: minor allele fraction ≥0.3 in disomic regions and in the [0.18,0.48] range in the trisomic region of Chr.10q

^b^ ≥1 link connecting ref, alt, HapA, and HapB & minor linkage ≤2 or minor linkage/total linkage ≤0.1

^c^With both the allele fraction (a) and the linkage (b) filter

To further test the reliability of our haplotype inference method against false variants in the input data, we performed haplotype inference on the RPE-1 data with all detected variants, including those in centromeric regions or on the short arm of Chr.21. The benchmark is summarized in Additional file 1:Table S4 with detailed metrics for each chromosome provided in Additional file 4. With the haplotype-linkage filter, the final haplotype solution shows similar overall accuracy (99.1%) but adds ≈40,000 phased variants in centromeric regions with ∼90% agreement with the reference data. With variants in centromeric or acrocentric regions excluded, the highest absolute error rate is 2.5% (Chr.17). Together, these results demonstrate the robustness of our haplotype inference method that contrasts with previous methods (“Whole-chromosome haplotype inference by HapCUT2” section).

### Haplotype inference with down-sampled data

To determine the minimum sequencing depth of each data type (linked reads and Hi-C) that is required to achieve whole-chromosome haplotype inference, we performed haplotype inference on randomly down-sampled variant-overlapping reads in the RPE-1 data. For both data types, we generated 66%, 50%, and 33% down-sampled reads from the original data; the benchmark metrics of the scaffold haplotype solution with each combination of linked-reads and Hi-C data are summarized in Additional file 1:Table S5 with additional metrics provided in Additional file 4. We confirmed that with >50% linked-reads and >50% Hi-C data, our method can reliably generate whole-chromosome haplotypes with >99% accuracy and >97% completeness relative to the original haplotype solution. The completeness of the haplotype solution is primarily determined by the depth of linked-reads data and drops to 95% with 33% linked reads. The depth of Hi-C sequencing controls long-range switching errors. For Chr.X that has the lowest average variant density, using 33% Hi-C reads results in large switching blocks (>10% of the entire chromosome) that can only be rescued with the original linked-reads data. Except for Chr.X, there is no significant long-range switching (resulting in overall accuracy <90%) even with 33% linked reads and 33% Hi-C reads.

We further performed haplotype inference on 11 × PacBio Circular-Consensus Sequencing data of RPE-1 cells in combination with the same Hi-C data and benchmarked the results against the reference haplotype. The results are summarized in Additional file 4. When the complete variant callset was used as input, we needed to choose a conservative switching cutoff (*ΔE*=250) to avoid switching errors due to false variants; this resulted in 1,922,469 phased variants (in contrast to 2,156,423 from 60x linked-reads) with 97.5% average accuracy. Chromosome X has the highest error rate ∼10% that is likely due to the combination of low variant density, low sequencing coverage, and the shorter range of molecular linkage of PacBio reads in comparison to linked reads. When we used high-quality variants (determined by the linkage filter from linked reads) as input, we could lower the switching cutoff to *ΔE*=5 and still preserve intra-block accuracy; the final haplotype solution contained 2,015,625 phased variants (93% of all high-quality variants) with 97.7% average accuracy.

We note that all the results generated from down-sampled data were derived using variants detected from the original linked-reads data. Therefore, these results only demonstrate the robustness of our haplotype inference algorithm but not the sufficiency to generate complete whole-chromosome haplotypes solely from the down-sampled data.

### Resolving chromosome-specific alterations using haplotype copy number

To demonstrate this application, we used the parental RPE-1 haplotypes to calculate haplotype-specific DNA copy number of aneuploid RPE-1 cells generated in a recent study [41]. In this study, the authors performed bulk whole-genome sequencing on the progeny populations of single RPE-1 cells that underwent telomere crisis. We downloaded and processed the sequencing data using the same workflow as described in “Sequence data processing” section and calculated haplotype-specific coverage in 250-kb bins as

3 $$\begin{array}{*{20}l} C_{A,B}^{(i)}=D^{(i)}\cdot \overline{R^{(i)}_{A,B}}. \end{array} $$

For each bin (*i*=1,2,⋯), *D*^(*i*)^ is the normalized mean sequence coverage ($\overline {D^{(i)}}=1$) and $\overline {R_{A,B}^{(i)}}$ is the mean haplotype fraction (A or B) across all variants. For a mostly diploid genome, the median value of *C*_*A,B*_ of all homologous chromosomes corresponds to the average coverage of a single homologous chromosome. We therefore normalized *C*_*A,B*_ by its median to calculate haplotype-specific DNA copy number.

**Fig. 5:**
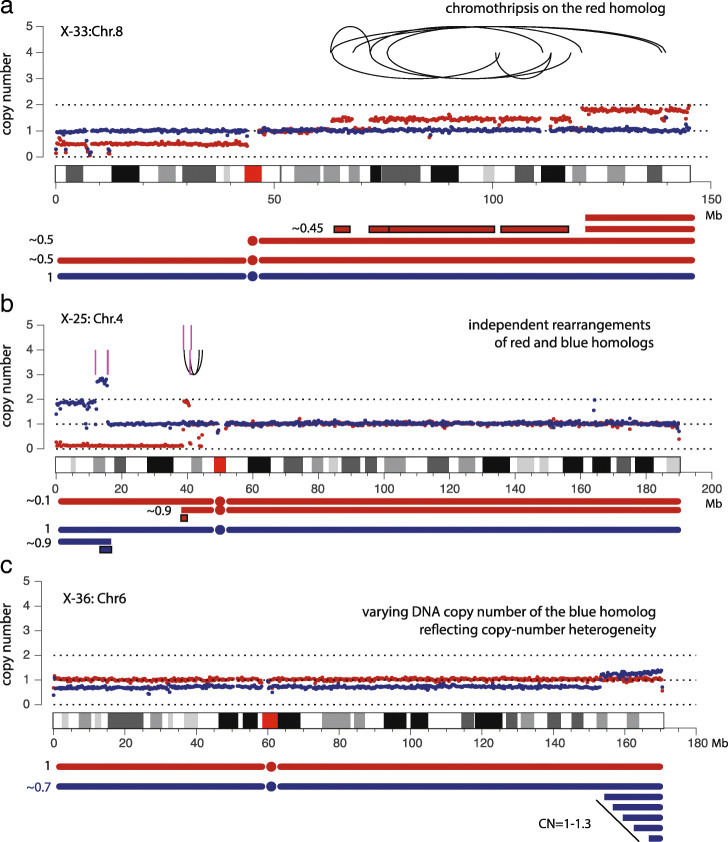
Haplotype-resolved DNA copy number and rearrangement analysis of post-crisis RPE-1 cells from Ref. [41]. Schematic diagrams of alterations to each homolog and their clonal fractions are shown below the copy-number plots. Non-telomeric chromosomal fragments involved in complex rearrangements are outlined. **a** Haplotype-specific DNA copy number and rearrangement (black arcs) of Chr.8 in the X-33 sample. Both rearrangements and copy-number alterations are restricted to the red homolog; the non-integer copy-number states indicate that the altered Chr.8 is present in a subclonal population (∼45%). **b** Haplotype-specific DNA copy number and rearrangement of Chr.4 (black arcs: intrachromosomal; magenta vertical lines: interchromosomal to Chr.13) in the X-25 sample. Rearrangements and copy-number alterations affect both homologs: Segmental changes of both homologs (blue: gain, red: loss) appear to be subclonal (∼90%). **c** Haplotype-specific copy number of Chr.6 in the X-36 sample. The blue homolog shows non-constant copy number (1-1.3) near the q-terminus that contrasts with constant copy number of the red homolog (∼1) or the rest of the blue homolog (∼0.7). We interpret this pattern as reflecting extensive copy-number heterogeneity in the population

Figure 5 shows three examples of chromosomes with complex alterations, each taken from a different sample that underwent telomere crisis. The DNA copy number of both haplotypes is shown using red and blue dots; chromosomal rearrangements related to copy-number alterations are shown as black arcs (intrachromosomal events) and magenta vertical lines (breakpoints of interchromosomal translocations). The first example (Fig. 5a) shows a chromothripsis event affecting the 8q arm of the red homolog. Based on the non-integer copy-number states of the red haplotype and the near diploid karyotype of this sample [41], we infer that the altered Chr.8q is present in a subclonal population (∼45%). The second example (Fig. 5b) shows alterations to both Chr.4 homologs on the p-arm: Both the gain of the blue haplotype and the loss of the red haplotype are subclonal (∼90%); the broken ends on both homologs are linked to Chr.13 (magenta lines), suggesting a complex event involving these three chromosomes. The last example (Fig. 5c) shows non-constant copy number of the blue haplotype at the q-terminus that contrasts with the constant copy number of the red haplotype or the rest of the blue haplotype. We interpret this copy number pattern as reflecting the retention of varying terminal segments in different cells in the population [48].

The haplotype copy-number analysis demonstrates that the progeny populations of single cells passing through telomere crisis can be highly heterogeneous and such heterogeneity can be identified directly from bulk DNA sequencing. The feature of non-constant haplotype copy number is of particular interest and may be used as a signature to infer ongoing genome instability in a cell population.

### Walking derivate chromosomes using haplotype-specific Hi-C contacts

Hi-C sequencing has previously been used to detect long-range chromosomal rearrangements [35, 49, 50]. The formation of new junctions between distal loci (separated by >1Mb genomic distance or located on different chromosomes) creates new *cis* contacts with a significantly higher density than *trans* contacts in a normal genome. With parental haplotype information, we can further phase rearrangement junctions and infer the organization of syntenic blocks in rearranged chromosomes from haplotype-specific Hi-C contacts and DNA copy number.

**Fig. 6:**
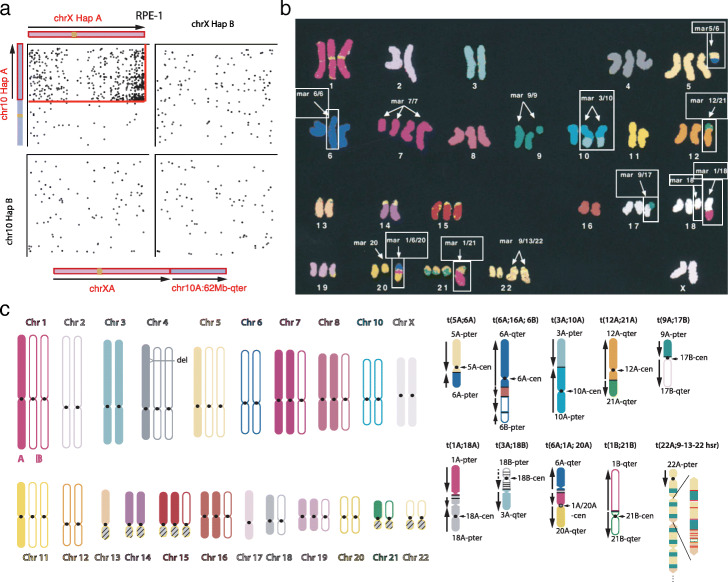
Haplotype-resolved synteny of rearranged chromosomes and aneuploid genomes. **a** Walking the translocated X chromosome in the RPE-1 genome using phased Hi-C links (dots) between different homologs of Chr.10 and Chr.X. A significant increase of Hi-C links is seen in only one haplotype combination reflecting *cis* links generated by the translocation between Chr.10 and Chr.X. The enrichment of Hi-C links throughout the entire X chromosome suggests the 10q segment is attached to an intact X chromosome with structure shown below the Hi-C contact map. Arrows denote the orientation of the two segments from the p-terminus to the q-terminus. **b** The cytogenetic K-562 karyotype reported in Ref. [42] (reprinted with permission from the publisher) with outlined structurally altered (marker) chromosomes resolved by sequencing data (shown in **c**). **c** The digital K-562 karyotype with haplotype assignment to both normal (left) and structurally altered (right) chromosomes determined from linked-reads and Hi-C sequencing data. The digital karyotype mostly agrees with the cytogenetic karyotype and the differences may be attributed to additional alterations during cell culture. Among all marker chromosomes listed in **b**, we are able to determine the syntenic structure of the following: mar5/6, mar6/6, mar3/10, mar12/21, mar9/17, mar1/18, mar1/6/20, and mar1/21, and resolve most rearrangement junctions at the base-pair level. Arrows represent the orientations of rearranged segments relative to the standard p-q arm orientation. Multiple junctions contained local fold-back rearrangements (inverted colored arrows in t(1A;18A), t(3A;18B), t(6A;1A;20A)) that are consistent with local DNA copy number gains; these events cannot be resolved by cytogenetic analyses. The mar18 described in Ref. [42] is probably related/similar to t(3A;18B). The *BCR-ABL* amplification is contained in a homogeneously staining region (hsr) in the marker chromosome t(22A;9-13-22hsr). We infer the structure of the amplicon from DNA copy number and rearrangements but cannot validate the inferred structure due to technical limitations. We are further able to partially resolve the structure of the altered Chr.7 and Chr.9 and completely resolve the structure of three additional marker chromosomes described in Ref. [43] but not in Ref. [42]: t(2A;22A), t(3A;10A;17A), t(9A;13A). Details of the analysis are presented in Additional file 5 and explained in Additional file 1:Determination of the K-562 karyotype by haplotype-specific genomic analysis

As each rearrangement breakpoint is originally generated on one parental chromosome, the newly formed *cis* contacts near the rearrangement junction should be phased to one haplotype on both sides of the junction. For interchromosomal rearrangements, *cis* contacts between the partner chromosomes should be observed in one out of four possible haplotype combinations (AA, AB, BA, or BB); for intrachromosomal rearrangements, newly formed *cis* contacts should be observed in one out of three possible combinations (AA, AB, or BB). Combining haplotype-specific connectivity from Hi-C contacts with haplotype DNA copy number from linked-reads data enables us to determine the structure of derivative chromosomes and generate phased karyotypes (Fig. 6).

We first illustrate this application using a simple example in the RPE-1 genome (Fig. 6a). RPE-1 cells contain a duplicated segment from Chr.10q (62 Mb-qter). The DNA sequence near the breakpoint on Chr.10q shows repeat sequence whose origin cannot be determined even with the PacBio data; cytogenetic analysis indicates that this segment is translocated to the q-terminus of Chr.X. In the phased Hi-C contact map, this translocation is easily recognized from the enrichment of contacts near the q-terminus of Chr.X and the breakpoint on Chr.10q (∼62 Mb) that is restricted to one haplotype combination (arbitrarily denoted as A for both chromosomes). Importantly, the enrichment of Hi-C contacts extends throughout Chr.X to the p-terminus, indicating that the 10q segment joins a complete X chromosome and confirming the result from cytogenetic analysis.

We further demonstrate this strategy by generating a “digital karyotype” of the K-562 genome using published sequencing data (Additional file 1:Table S1). The K-562 genome is highly aneuploid [50] and contains multiple structurally abnormal (marker) chromosomes [42, 43] (Fig. 6b) and large regions of loss-of-heterozygosity (LOH). We first determined the parental haplotypes in heterozygous regions from linked-reads and Hi-C data and then calculated haplotype-specific DNA copy number using phased coverage in the linked-reads data. We next determined the linkage between rearranged chromosomal segments using both phased molecular linkage from the linked-reads data and long-range haplotype-specific Hi-C contacts near copy-number breakpoints. The digital karyotype was constructed by a joint analysis of haplotype-specific DNA copy number, rearrangements, and Hi-C contacts and is schematically shown in Fig. 6c. Details of this analysis are presented in Additional file 1:Determination of the K-562 karyotype by haplotype-specific genomic analysis accompanying results presented in Additional file 5. The digital karyotype shows excellent agreement with results by cytogenetic analyses reported in Ref. [42] (Fig. 6b) and [43]. In addition to resolving the synteny of rearranged chromosomal segments, the digital karyotype resolves the parental origin of each segment and the rearrangement junctions with base-pair resolution in 9 marker chromosomes reported in Ref. [42] (outlined in Fig. 6b and schematically shown in Fig. 6c) and 3 additional marker chromosomes reported in [43]. We also partially resolved the structure of the complex amplicon containing the *BCR-ABL* fusion in t(22A;9-13-22hsr) combining sequencing and cytogenetic data.

## Discussion

Here we describe a computational method that can accurately determine complete chromosomal haplotypes using a combination of linked-reads sequencing (30-60 × mean depth) and Hi-C sequencing data (≥50 million long-range contacts). The computationally inferred haplotypes show high accuracy (>99%) and completeness (>98%) when compared to reference haplotype data directly obtained from parental chromosomes.

Our method offers several advantages over previous methods. First, both linked-reads and Hi-C sequencing data can be generated on standard sequencing platforms and the construction of sequencing libraries does not involve special experimental techniques required for single-chromosome isolation [21–23], single-cell sequencing [24], or similar techniques such as “Strand-Seq” [25, 51]. Second, the computational algorithm implicitly excludes inconsistent linkage evidence from false variants based on the specificity of haplotype linkage. This contrasts with previous methods [37] that require high-quality variants as input (“Whole-chromosome haplotype inference by HapCUT2” section). Our method further enables a variant-filtering strategy based on haplotype linkage that can be used to exclude false variants due to alignment errors and validate complex variants such as insertions, deletions, or large structural variants.

Our formalism of haplotype inference as a minimization problem also has several unique features. The symmetric representation of binary genotypes and haplotypes simplifies the inference of complementary parental haplotypes into one minimization problem based on linkage evidence from both parental chromosomes. The haplotype inference algorithm is not affected by allelic imbalance, including loss-of-heterozygosity, and is directly applicable to aneuploid tumor genomes (demonstrated in the K-562 example). We demonstrate that a simple iteration strategy can efficiently solve the parental haplotypes of diploid genomes but it is straightforward to incorporate more sophisticated minimization algorithms (e.g., Monte-Carlo methods) when necessary (Additional file 1:Haplotype inference and energy minimization of the 1D spin model).

A key feature of our method in contrast to others [37, 39] is that it is designed to completely eliminate large block-switching errors using Hi-C contacts. Even with low-coverage linked-reads or PacBio data (10-20 ×), the scaffold haplotype solution generated by concatenation of local haplotype blocks using Hi-C links shows consistent global phasing accuracy (>95%) relative to a single parental haplotype. One useful extension of our method is to perform joint haplotype inference using population genotypes and Hi-C data. Population-based statistical phasing [16] can produce long haplotype blocks (>1Mb) that contain random but rare switching errors. It should be possible to correct these errors using Hi-C data and determine the complete haplotype phase of common variants on individual chromosomes [39], which can then be used to generate phased Hi-C contact maps.

A major limitation of alignment-based analysis (especially of short reads) is that it cannot resolve repetitive sequences or sequences that are highly divergent from the reference. Inaccurate alignment of sequencing reads derived from repetitive or highly divergent sequences can lead to both false-positive and false-negative variant detection. Although our method can filter false-positive variants based on haplotype linkage, it cannot rescue missed variants due to incorrect alignment. Resolving haplotype linkage in these regions requires different strategies such as long-read sequencing or de novo assembly.

Knowledge of chromosomal haplotypes can be used to directly relate variations in the DNA sequence, histone marks, chromatin structure, and gene expression on each chromosome. This is especially useful for the analysis of cancer genomes where homologous chromosomes often acquire independent alterations that can cause differential changes in chromatin organization or gene expression [52]. We demonstrate the feasibility to determine the synteny of derivative chromosomes in aneuploid genomes directly from sequencing data by constructing a digital karyotype of the K-562 genome using linked-reads and Hi-C sequencing data. We expect this strategy to be generally applicable to complex cancer genomes and useful for investigating the connection between 2D chromosomal structural alterations and 3D chromatin reorganization.

## Conclusions

We describe a computational strategy to determine complete parental haplotypes of diploid genomes and haplotype-resolved karyotypes of aneuploid genomes using a combination of bulk long-range sequencing and Hi-C sequencing.

## Methods

### Generation of sequencing data

#### Bulk linked-reads sequencing data of RPE-1 cells

The RPE-1 linked-reads data were generated at the Yale Center for Genome Analysis. High-molecular weight DNA from RPE-1 cells was extracted using the RevoluGen PuriSpin Fire Monkey kit following the protocol provided by the vendor with the following modifications: Cells were lysed at 56 ^∘^C for 2 h, followed by addition of ∼100 ng RNase A and additional incubation for 15 min at 56 ^∘^C. A single linked-reads library was constructed using the Chromium Genome Library Kit v2 from 10X Genomics following the standard protocol. The library was then sequenced on the Illumina NovaSeq platform to generate 941,518,426 read pairs with 60 × mean depth of coverage. See Table 1 for additional metrics of the sequencing data.

#### PacBio Circular Consensus Sequencing data of RPE-1 cells

PacBio Circular Consensus Sequencing data of a progeny population derived from a single cell were generated at the Broad Institute. A total of 4,607,047 High-Fidelity (Hi-Fi) reads were generated after circular consensus correction with N50 read length 7.3kb. The mean sequence coverage is ∼11×. The sequencing data will be released at the NCBI Short Read Archive as SRR13579109.

#### Sequencing data of monosomic RPE-1 cells

Monosomic RPE-1 cells were generated using three different strategies: (1) Nocodazole block and release [24]; (2) Induction of dicentric chromosome bridges [48]; and (3) Treatment with Paclitaxel, a spindle toxin that induces tetraploidization by preventing microtubulin disassembly. All three strategies significantly increase the frequency of chromosome missegregation and the generation of monosomic daughter cells. Monosomic cells were first selected based on the arm-level DNA copy number estimated from low-pass (0.1×) whole-genome sequencing and then sequenced to 5–30 × on either the Illumina HiSeq 2500 or the Illumina NovaSeq platforms at the Broad Institute of MIT and Harvard. We then identified and validated completely monosomic chromosomes based on the “normalized heterozygosity” [24] in the deep sequencing data defined as

$$\frac{\text{observed heterozygosity}}{(\text{observed allelic coverage})^{2}}=\frac{p_{\text{het}}}{(p_{\text{ref}}+p_{\text{alt}})^{2}/4}. $$

The *observed heterozygosity* *p*_het_ is defined as the fraction of parental heterozygous sites that show heterozygous coverage in a single-cell genome; the *observed allelic coverage* is defined as the median of the fraction of heterozygous sites showing reference coverage *p*_ref_ and the fraction of heterozygous sites showing alternate coverage *p*_alt_, which is roughly equal to the average coverage of each parental chromosome in disomic regions in a single cell genome [24]. Heterozygous variants in the parental genome were detected using the bulk sequencing data as described below in the “Variant calling and filtering” section. To eliminate false heterozygosity due to sequencing or amplification errors in the single-cell data, we considered a variant site to show reference or alternate coverage only when the number of sequencing reads showing either genotype exceeds a threshold set as *d*^∗^= max(2,1+0.1×mean sequencing depth of chromosome): *d*^∗^=2 if the mean sequencing depth is ≤10× (most samples) and *d*^∗^=4 if the mean sequencing depth is 30 ×. The minimum threshold of 2 reads was used to eliminate random sequencing errors; the threshold of 0.1× mean sequencing depth served to exclude low frequency (<10%) amplification errors. Complete monosomies were selected based on the criteria that the normalized heterozygosity is less than 0.1× the median from all diploid cells (≈ 1). For the current study, we selected 39 cells with one or multiple monosomic chromosomes (32 from nocodazole release, 5 from bridge induction, and 2 from Paclitaxel treatment), containing 98 monosomic chromosomes in total. The sample names, mean sequencing depths, and the normalized heterozygosity of monosomic chromosomes are listed in Additional file 6.

### Sequence data processing

All the sequencing data listed in Table 1 and S1 were re-processed starting from unmapped sequencing reads. For the linked-reads data, we used the LongRanger software from 10X Genomics to extract the molecular barcode of each sequencing fragment that was preserved in the “BX” tag in the BAM record. The molecular barcode information was only used as molecular linkage evidence but not for sequence alignment. Alignment and post-alignment processing of all sequencing data except the K-562 linked-reads data were completed using the same pipeline as described below. For the K-562 linked-reads data, we used the output from LongRanger for downstream analysis.

#### Sequence data alignment

We aligned all sequencing data (both linked reads and Hi-C) using a standard short-read aligner (https://github.com/lh3/bwa) with default parameters (“bwa mem”). Using a barcode-agnostic aligner ensures better specificity of linkage information (and therefore better phasing accuracy) than using a barcode-aware aligner such as Lariat (https://github.com/10XGenomics/lariat) in the LongRanger pipeline. The rationale is explained below in Additional file 1:Linkage evidence from molecular identifier and sequence alignment of linked reads.

The PacBio CCS data of RPE-1 cells were aligned using minimap2 (https://github.com/lh3/minimap2) with the following command: minimap2 -ax map-pb.

#### Post-alignment processing

When choosing the primary alignment positions of sequencing reads with multiple alignment positions (supplementary or secondary alignments), we gave preference to alignment positions consistent with the proper-pair configuration, i.e., placing the two mates at the forward-reverse orientation with inferred insert size within the 0.1% and 99.9% percentile of the insert size histogram. The insert size histogram was generated for each sequencing library from 2,000,000 uniquely (both mates having mapping quality 60) and properly (two mates are placed at the forward-reverse orientation with <2000 bp separation) aligned read pairs based on the alignment positions of pairmates. We used the MarkDuplicates program in Picard (https://broadinstitute.github.io/picard/) to infer sequencing reads corresponding to PCR duplicates based on the primary alignment positions and adjusted the duplication tag of both primary and supplementary alignments accordingly.

### Variant calling and filtering

We ran the HaplotypeCaller program from GATK (v4.0.12.0-6-gfef36e3-SNAPSHOT) in the discovery mode (“--genotyping-mode DISCOVERY”) to detect genetic variants. We imposed the following read filters in addition to the standard parameters and read filters used by HaplotypeCaller to exclude reads with improper, inaccurate, or low-confidence mapping:


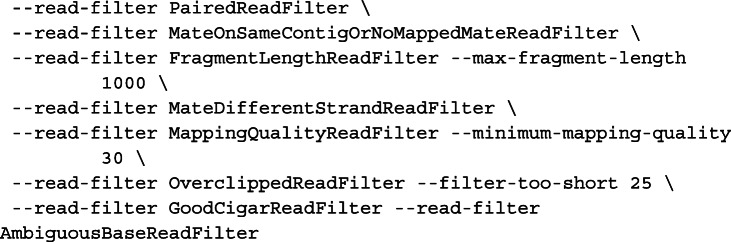


For the RPE-1 genome, variant discovery was performed jointly on the new linked-reads data (60×) and the previously published standard whole-genome data (13×) [53]. For the NA12878 genome, variant discovery was performed on both linked-reads data (35× each) [54].

We selected bi-allelic single-nucleotide variant sites (one reference plus one alternate) as the input for haplotype inference, excluding sites in pericentric, acrocentric, and centromeric regions based on the standard chromosome banding annotation (“acen,” “gvar,” “stalk”) provided by the UCSC genome browser. No other filter (e.g., variant quality score recalibration) was applied.

### Haplotype inference from linkage evidence

We first introduce a binary numerical representation of genotypes at heterozygous variants as +1 for the reference base and −1 for the alternate base. A haplotype block consisting of *N* variant sites is represented as a vector

$$\mathbf{S}=\left(s_{1},s_{2},\cdots, s_{N}\right),\quad s_{i}=\pm1.$$

Similarly, a molecular link with genotype information at multiple variant sites is represented as

$$\boldsymbol{\sigma}=\left(\sigma_{1},\sigma_{2},\cdots\right), \quad \sigma_{i}=\pm1.$$

Using the binary genotype representation, we can simplify four types of linkage between genotypes (either *σ*_*i*_ or *s*_*i*_)

$$\begin{array}{*{20}l} \begin{array}{ll} \text{reference-reference linkage:} & s_{i}=1,s_{j}=1; \\ \text{alternate-alternate linkage:} & s_{i}=-1,s_{j}=-1; \\ \text{reference-alternate linkage:} & s_{i}=1,s_{j}=-1; \\ \text{alternate-reference linkage:} & s_{i}=-1,s_{j}=1. \end{array} \end{array} $$

into two types of haplotype linkage

$$\begin{array}{*{20}l} \begin{array}{ll} \text{reference-reference/alternate-alternate linkage:} & s_{i}\cdot s_{j}=1, \\ \text{reference-alternate/alternate-reference linkage:} & s_{i}\cdot s_{j}=-1. \end{array} \end{array} $$

Moreover, a molecular link (*σ*_*i*_,*σ*_*j*_) between sites *i* and *j* is consistent with haplotype linkage (*s*_*i*_,*s*_*j*_) if and only if

$$\sigma_{i}\sigma_{j}s_{i}s_{j}=1.$$

If the error probability of a molecular link is given by *ε*_*ij*_, then

$$p(\sigma_{i}\sigma_{j}s_{i}s_{j}=1)=1-\epsilon_{{ij}};\quad p(\sigma_{i}\sigma_{j}s_{i}s_{j}=-1)=\epsilon_{{ij}}.$$

Assuming a uniform prior probability *p*(*s*_*i*_*s*_*j*_=1)=*p*(*s*_*i*_*s*_*j*_=−1)=1/2, we can re-write the above equation as

$$\frac{p(\sigma_{i}\sigma_{j}|s_{i}s_{j}=1)}{p(\sigma_{i}\sigma_{j}|s_{i}s_{j}=-1)}=\left(\frac{1-\epsilon_{{ij}}}{\epsilon_{{ij}}}\right)^{\sigma_{i}\sigma_{j}}.$$

Extending this to a collection of links $\left \{\sigma _{i}^{(k)}\sigma _{j}^{(k)},1\leq k\leq n\right \}$, we have

4 $$ \frac{p\left(\left\{\sigma_{i}^{(k)}\sigma_{j}^{(k)}\right\}|s_{i}s_{j}=1\right)}{p\left(\left\{\sigma_{i}^{(k)}\sigma_{j}^{(k)}\right\}|s_{i}s_{j}=-1\right)}=\prod_{k}\left(\frac{1-\epsilon^{(k)}_{{ij}}}{\epsilon^{(k)}_{{ij}}}\right)^{\sigma^{(k)}_{i}\sigma^{(k)}_{j}},  $$

which leads to the following log-likelihood function

5 $$\begin{array}{*{20}l} L\left(\left\{\sigma_{i}^{(k)}\sigma_{j}^{(k)}\right\}|s_{i}s_{j}\right)&=s_{i}s_{j}\left[\ln p\left(\left\{\sigma_{i}^{(k)}\sigma_{j}^{(k)}\right\}|s_{i}s_{j}=1\right)- \ln p\left(\left\{\sigma_{i}^{(k)}\sigma_{j}^{(k)}\right\}|s_{i}s_{j}=-1\right)\right]\\ &=\sum_{k}\sigma^{(k)}_{i}\sigma^{(k)}_{j}s_{i}s_{j}\ln\left(1-\epsilon_{{ij}}^{(k)}/\epsilon_{{ij}}^{(k)}\right). \end{array} $$

If we assume a constant error rate for all links, $\epsilon _{{ij}}^{(k)}=\epsilon $, Eq. (5) is simplified to

6 $$\begin{array}{*{20}l} L\left(\left\{\sigma_{i}^{(k)}\sigma_{j}^{(k)}\right\}|s_{i}s_{j}\right)&=\ln\left(\frac{1-\epsilon}{\epsilon}\right)\sum_{k} \sigma^{(k)}_{i}\sigma^{(k)}_{j}s_{i}s_{j} \\ &\propto\underbrace{\#(\sigma_{i}\sigma_{j}s_{i}s_{j}=1)}_{\text{consistent links}}-\underbrace{\#(\sigma_{i}\sigma_{j}s_{i}s_{j}=-1)}_{\text{inconsistent links}}. \end{array} $$

The haplotype linkage inferred from all molecular links is given by

7 $$\begin{array}{*{20}l} s_{i}s_{j}=\left\{\begin{array}{ll} 1 & \sum_{k}\sigma^{(k)}_{i}\sigma^{(k)}_{j}>0;\\ -1 & \sum_{k}\sigma^{(k)}_{i}\sigma^{(k)}_{j}<0\end{array} \right. \end{array} $$

We can generalize Eq. (5) to *N* variants as

8 $$\begin{array}{*{20}l} L\left(\left\{\boldsymbol{\sigma}^{(k)}\right\}|\mathbf{S}\right)=\frac{1}{2}\sum_{1\leq i,j\leq N}s_{i}s_{j}\sum_{k}\sigma^{(k)}_{i}\sigma^{(k)}_{j}\ln\left(1-\epsilon_{{ij}}^{(k)}/\epsilon_{{ij}}^{(k)}\right), \end{array} $$

and solve for the optimal haplotype solution **Ŝ** by maximizing Eq. (8). We further assume a constant frequency of incorrect molecular linkage

9 $$\begin{array}{*{20}l} \epsilon_{{ij}}^{(k)}=\epsilon_{{ij}}. \end{array} $$

With this approximation, we can then simplify Eq. (8) as

10 $$\begin{array}{*{20}l} L\left(\left\{\sigma^{(k)}\right\}|\mathbf{S}\right)&=\frac{1}{2}\sum_{1\leq i,j\leq N}s_{i}s_{j}\ln\left(1-\epsilon_{{ij}}/\epsilon_{{ij}}\right)\sum_{k}\sigma^{(k)}_{i}\sigma^{(k)}_{j}\\ &=\frac{1}{2}\sum_{1\leq i,j\leq N}s_{i}s_{j}\ln\left(1-\epsilon_{{ij}}/\epsilon_{{ij}}\right)(n_{{ij}}^{+}-n_{{ij}}^{-}), \end{array} $$

where we have introduced

11 $$\begin{array}{*{20}l}  n^{+}_{{ij}}&=\#\left(\sigma^{(k)}_{i}\sigma^{(k)}_{j}=1\right)=n_{{ij}}^{\text{RR}}+n_{{ij}}^{\text{AA}}, \end{array} $$

12 $$\begin{array}{*{20}l} n^{-}_{{ij}}&=\#\left(\sigma^{(k)}_{i}\sigma^{(k)}_{j}=-1\right)=n_{{ij}}^{\text{RA}}+n_{{ij}}^{\text{AR}} \end{array} $$

as the number of links consistent with either type of haplotype linkage between site *i* and *j*.

The rationale for the approximation in Eq. (9) is that we expect incorrect linkage due to either random errors (generated in library construction or sequencing) or incorrect sequence alignment to affect each molecule with the same probability. However, incorrect alignment can be significantly enriched near variants detected in low-complexity regions. We therefore estimate *ε*_*ij*_ from the observed linkage evidence as

13 $$ \epsilon_{{ij}}=\max\left[\epsilon_{0},\frac{\min\left(n_{{ij}}^{+},n_{{ij}}^{-}\right)}{n_{{ij}}^{+}+n_{{ij}}^{-}}\right].  $$

Here $\min (n_{{ij}}^{+},n_{{ij}}^{-})/(n_{{ij}}^{+}+n_{{ij}}^{-})$ is the observed fraction of minor haplotype linkage between two variant sites *i* and *j*; $\min (n_{{ij}}^{+},n_{{ij}}^{-})=0$ if there is no discordant haplotype linkage. *ε*_0_ reflects random errors and can be estimated using the average fraction of observed discordant linkage

$$\begin{array}{*{20}l} \epsilon_{0}=\left\langle\frac{\min\left(n_{{ij}}^{+},n_{{ij}}^{-}\right)}{n_{{ij}}^{+}+n_{{ij}}^{-}}\right\rangle. \end{array} $$

The formalism of haplotype inference defined in Eqs. (8) and (10) has several advantages. First, the binary representation of haplotype phase and molecular linkage preserves the symmetry between parental haplotypes (**S** and −**S**) or molecular links derived from parental chromosomes (***σ*** and −***σ***). This is convenient for performing haplotype inference in aneuploid genomes where one homolog may contribute dominant linkage evidence (e.g., in hemizygous or trisomic regions).

Second, the formalism is directly applicable to haplotype block phasing. For example, we can represent the parental haplotype using local blocks **B**_*i*_ and their haplotype phase *b*_*i*_=±1 as

14 $$\begin{array}{*{20}l} \mathbf{B}=b_{1}\mathbf{B}_{1} + b_{2}\mathbf{B}_{2} + \cdots b_{m}\mathbf{B}_{m}.  \end{array} $$

$\overline {\mathbf {B}_{k}}=-\mathbf {B}_{k}$ is the complementary phase of **B**_*k*_. We can calculate inter-block molecular linkage (similar to Eq. (11)) as

15 $$\begin{array}{*{20}l} n_{{st}}^{+}=n(\mathbf{B}_{s}\leftrightarrow\mathbf{B}_{t})+n(\overline{\mathbf{B}_{s}}\leftrightarrow\overline{\mathbf{B}_{t}}); \end{array} $$

16 $$\begin{array}{*{20}l} n_{{st}}^{-}=n(\overline{\mathbf{B}_{s}}\leftrightarrow\mathbf{B}_{t})+n(\mathbf{B}_{s}\leftrightarrow\overline{\mathbf{B}_{t}}) \end{array} $$

and solve for the haplotype phase (*b*_1_,*b*_2_,⋯*b*_*m*_) by maximizing the log-likelihood function that is similar to Eq. (10).

Finally, maximizing Eq. (10) is equivalent to minimizing the energy of a 1D Ising (spin glass) model

17 $$\begin{array}{*{20}l} E(\mathbf{S})=-\frac{1}{2}\sum_{1\leq i,j\leq N}M_{{ij}}s_{i}s_{j} \end{array} $$

with finite range interactions $M_{{ij}}=(n_{{ij}}^{+}-n_{{ij}}^{-})\ln (1-\epsilon _{{ij}}/\epsilon _{{ij}})$, for which there are many existing approaches. Here we solve this problem by introducing two types of perturbations:

$$\begin{array}{*{20}l} \text{spin flip:} \; & (\cdots s_{i-1}, s_{i}, s_{i+1},\cdots) \rightarrow (\cdots s_{i-1}, -s_{i},s_{i+1},\cdots) \\ \text{block switch:} \; & (s_{1},\cdots s_{i},s_{i+1},\cdots s_{N})\rightarrow (s_{1},\cdots s_{i},-s_{i+1},\cdots -s_{N}). \end{array} $$

The changes to *E*(**S**) due to these perturbations are given by

18 $$\begin{array}{*{20}l} \Delta E_{i}=s_{i}\sum_{j}M_{{ij}}s_{j}, \quad \text{(spin flip)} \end{array} $$

and

19 $$\begin{array}{*{20}l} \Delta E_{k|k+1}=\sum_{i\leq k}\sum_{j>k} M_{{ij}}s_{i}s_{j}. \quad \text{(block switch)} \end{array} $$

It can be shown that through iterations of spin flipping and block switching, one can always find (one of) the optimal haplotype solution **Ŝ** that minimizes Eq. (17) if the majority of molecular linkage is consistent with *cis* haplotype linkage (Additional file 1:Haplotype inference and energy minimization of the 1D spin model). For two haplotype configurations **S** and *S*^′^, the energy difference is related to the likelihood ratio

$$\Delta E=E(\mathbf{S})-E(\mathbf{S'})=L(\sigma^{(k)}|\mathbf{S})-L(\sigma^{(k)}|\mathbf{S'})=\ln \frac{p\left(\sigma^{(k)}|\mathbf{S}\right)}{p\left(\sigma^{(k)}|\mathbf{S'}\right)},$$

and the probability of phasing errors is given by

20 $$\begin{array}{*{20}l} \delta=\frac{1}{1+e^{\Delta E}}.  \end{array} $$

A low energy penalty score (*ΔE*≈0) implies low phasing confidence (*δ*≈0.5), and vice versa (*ΔE*≫0⇒*δ*≈0). *ΔE*_*i*_ or *ΔE*_*i*|*i*+1_ can therefore be used to estimate the probability of local phasing errors (*s*_*i*_→−*s*_*i*_) and long-range switching errors (*s*_*j*>*i*_→−*s*_*j*>*i*_).

The spin-flipping energy penalty *ΔE*_*i*_ can be rewritten as

21 $$\begin{array}{*{20}l} \Delta E_{i}&=s_{i}\sum_{j\neq i} M_{{ij}}s_{j}= s_{i}\sum_{j\neq i} (n_{{ij}}^{+}-n_{{ij}}^{-})s_{j}\underbrace{\ln\left(1-\epsilon_{{ij}}/\epsilon_{{ij}}\right)}_ {\chi_{{ij}}}\\ &=s_{i}\left[\sum_{j\neq i, s_{j}=1}\chi_{{ij}}(n_{{ij}}^{+}-n_{{ij}}^{-})-\sum_{j\neq i,s_{j}=-1}\chi_{{ij}}(n_{{ij}}^{+}-n_{{ij}}^{-})\right]\\ &=s_{i}\underbrace{\left(\sum_{j\neq i, s_{j}=1}\chi_{{ij}}n_{{ij}}^{+}+\sum_{j\neq i,s_{j}=-1}\chi_{{ij}}n_{{ij}}^{-}\right)}_{\eta_{R\leftrightarrow\mathbf{S}}} - s_{i}\underbrace{\left(\sum_{j\neq i, s_{j}=1}\chi_{{ij}}n_{{ij}}^{-}+\sum_{j\neq i,s_{j}=-1}\chi_{{ij}}n_{{ij}}^{+}\right)}_{\eta_{A\leftrightarrow\mathbf{S}}}  \end{array} $$

The two terms *η*_*R*⇔**S**_ and *η*_*A*⇔**S**_ in Eq. (21) measure the total linkage between the genotypes at site *i* (*R* for reference and *A* for alternate) and the haplotypes of parental chromosomes (**S** and −**S**) and are related to Eq. (1). For true heterozygous variants, reference and alternate genotypes are phased to complementary haplotypes, i.e., *R*⇔**S** (and hence *A*⇔**−S**), or *A*⇔**S** (*R*⇔**−S**). This implies that either *η*_*R*⇔**S**_≫*η*_*A*⇔**S**_≈0 or *η*_*A*⇔**S**_≫*η*_*R*⇔**S**_≈0. As the genotypes of false variants are generally not phased to complementary haplotypes, false variants tend to have low phasing confidence (*ΔE*≈0) and can be excluded from the haplotype solution based on this feature. Moreover, linkage evidence from false variants is offset by the *χ*_*ij*_ factor due to the presence of significant discordant linkage (*ε*_*ij*_≫*ε*_0_). These features make our haplotype inference method robust against the presence of ambiguous haplotype linkage due to false variants.

#### Phased Hi-C linkage between haplotype blocks

The signal of inter-block Hi-C linkage defined in Eq. (15) is calculated as follows: For two haplotype blocks **B**_*s*_ and **B**_*t*_, the number of Hi-C links supporting *cis*-linkage is given by

22 $$\begin{array}{*{20}l} n_{{st}}^{+}=n\left(\mathbf{B}_{s}\leftrightarrow\mathbf{B}_{t}\right)+n\left(\overline{\mathbf{B}}_{s}\leftrightarrow\overline{\mathbf{B}}_{t}\right)=\#\left[\sigma^{(m)}(x_{m})\sigma^{(m)}(y_{m})\mathbf{B}_{s}(x_{m})\mathbf{B}_{t}(y_{m})=1\right], \end{array} $$

where the count runs over all Hi-C links {***σ***^(*m*)^|*m*=1,2,⋯ } with variant positions *x*_*m*_ and *y*_*m*_ in haplotype blocks **B**_*s*_ and **B**_*t*_. Similarly, the signal of *trans*-linkage is given by

23 $$\begin{array}{*{20}l} n_{{st}}^{-}=n\left(\mathbf{B}_{s}\leftrightarrow\overline{\mathbf{B}}_{t}\right)+n\left(\overline{\mathbf{B}}_{s}\leftrightarrow\mathbf{B}_{t}\right)=\#\left[\sigma^{(m)}(x_{m})\sigma^{(m)}(y_{m})\mathbf{B}_{s}(x_{m})\mathbf{B}_{t}(y_{m})=-1\right]. \end{array} $$

The specificity of Hi-C linkage between haplotype blocks is very sensitive to long-range switching errors within blocks. For example, consider two haplotype blocks with fractions of haplotype A given by *f*_1_ and *f*_2_. If we assume all Hi-C links to be intra-molecular, then the fraction of apparent *cis*-linkage between these two blocks is approximately

$$\rho=\underbrace{f_{1}\cdot f_{2}}_{A\leftrightarrow A}+\underbrace{(1-f_{1})\cdot(1-f_{2})}_{B\leftrightarrow B},$$

and the fraction of apparent *trans*-linkage is

$$1-\rho=f_{1}+f_{2}-2f_{1}f_{2}.$$

When there is no switching error within either block, *f*_1,2_=0 or1 and *ρ*=0 or 1, the Hi-C links have the most specific signal. The presence of long-range switching errors in either block (0<*f*_1_<1 or 0<*f*_2_<1) reduces the specificity of Hi-C linkage; in particular, when *f*_1_≈0.5 or *f*_2_≈0.5 (which can be caused by a single switching error), *ρ*≈1−*ρ*≈1/2.

#### Software implementation

We have implemented a C++ package “mLinker” that performs multiple tasks related to haplotype inference. For a detailed description of the software package, see Additional file 1:Software implementation of the haplotype inference algorithm.

#### Assessing phasing accuracy and determining high-confidence haplotype blocks

The haplotype solution produced by our algorithm includes both phased genotypes at variant sites and two penalty scores (Eqs. (18) and (19)) that measure the confidence of haplotype inference (Eq. (20)) at each variant site. The spin-flipping energy score (Eq. (18)) can be used to exclude single variants with low phasing confidence; the block-switching energy score (Eq. (19)) can be used to identify sites with a high switching-error probability as the boundaries of high-confidence haplotype blocks.

If the switching-penalty cutoff is too permissive, the presence of intra-block switching will compromise the specificity of Hi-C linkage (see “Phased Hi-C linkage between haplotype blocks” section); if the switching-penalty cutoff is too stringent, the resulting phase blocks are too short, which also leads to weaker Hi-C linkage. For the linked-reads data (including down-sampled data), we observed a local minimum in the switching penalty distribution that is ∼0.1× median coverage (Additional file 1:Fig. S6A). This is a conservative cutoff that always ensures intra-block accuracy of local haplotype blocks but also generates sufficient inter-block Hi-C linkage for haplotype concatenation. For the PacBio data (∼11×), we did not see a local minimum in the switching penalty distribution; this may be due to either the low sequencing depth or the shorter range of molecular haplotype linkage of the PacBio data in comparison to the linked-reads data.

To further assess what is the optimal switching penalty cutoff for haplotype block concatenation, we calculated the percentage of variants in long phase blocks (≥50 phased variants) with ≥98% phasing accuracy, the percentage of variants in long phase blocks (≥50 phased variants) with less than 98% accuracy, and the percentage of variants in short phase blocks (<50 variants). These results are summarized in Additional file 1:Fig.S6B (linked reads) and S6C (PacBio). If we use the fraction of variants in high-accuracy (>98%) long phase blocks (≥50 phased variants) as a measure of local haplotype accuracy, then the optimal cutoff is estimated to be 50-200 for the linked-reads data and 5-10 for the PacBio data. These values should be taken as the minimum threshold for determining local haplotype blocks. To ensure the best accuracy of the final haplotype solution, we recommend choosing a more conservative cutoff to avoid any potential switching errors, especially those in low variant-density regions, as long as the N50 phase block size is above 100 kb (to generate sufficient Hi-C linkage). One can also validate the accuracy of local haplotype blocks by the number of *cis* and *trans* inter-block Hi-C links (Additional file 1:Fig. S5).

### Whole-chromosome haplotype inference by HapCUT2

HapCUT2 was originally described in Ref. [37] and can perform haplotype inference on long-read (PacBio), linked-reads, and Hi-C sequencing. The authors of HapCUT2 mentioned that using a combination of 40 × coverage Hi-C data with 34 × linked-reads data, they could assemble haplotypes with 98.9% of variants contained in the largest block for each chromosome, with an average switch error rate of 0.0008 and mismatch rate of 0.003, but did not provide the absolute error rate or the completeness of the haplotype solution of each chromosome.

Given the similar features and input data types of HapCUT2 and mLinker, we compared their performance of whole-chromosome haplotype inference. For haplotype inference from linked-reads and Hi-C data, we ran HapCUT2 (v1.3.1) on both NA12878 and RPE-1 data as follows. Aligned linked-reads data were first converted to the fragment file format with the following commands:


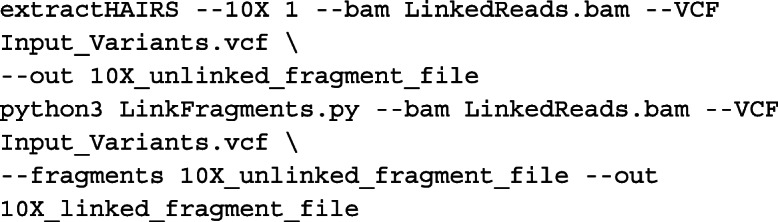


Aligned Hi-C reads were also converted to the fragment file format by the following command:


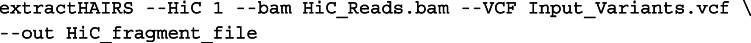


Finally, the linked-reads and Hi-C fragment files were merged and assembled into a single haplotype for each chromosome with the following commands:


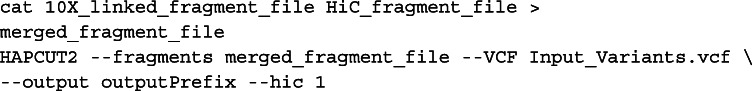


As HapCUT2 does not filter variants with ambiguous linkage, its accuracy depends on the specificity of input variants [37]. We ran two instances for both NA12878 and RPE-1 genomes, first using unfiltered heterozygous variants excluding those in centromeric regions, and then using high-quality variants defined as those phased by mLinker and passing the linkage filter (Tables 2 and 3). The specificity of unfiltered variants is around 90% when estimated by the fraction of unfiltered variants passing the linkage filter, or by the fraction of unfiltered variants that are contained in the truth data. The haplotype solutions from HapCUT2 were benchmarked using the same truth data (trio phase of NA12878 and monosomy phase of RPE-1) as described in “Benchmark of the haplotype solution” section on all phased variants. The results are summarized in Additional file 7.

When unfiltered variants were used as input, multiple chromosomes in the NA12878 solution and all chromosomes in the RPE-1 solution showed >2% error rate; two chromosomes in the NA12878 solution (Chr.9 and Chr.12) and seven chromosomes in the RPE-1 solution showed >10% error rate. Three examples of HapCUT2 solutions containing >10% error rate are shown in Additional file 1:Fig. S7. Some switching errors (e.g., the blue blocks in the 9q arm of NA12878) occur near regions of low variant density (measured by the number of high-quality variants in the truth data in 0.5Mb bins, second track in each panel); others (12q of NA12878 and 19q of RPE-1) are not related to low-variant density. The first example (Chr.9 in NA12878) also shows haplotype switching between p- and q-arms. When only high-quality variants were used, the accuracy of HapCUT2 solutions was comparable to mLinker (RPE-1: 0.9% for mLinker, 1.1% for HapCUT2; NA12878: 0.3% for both). The higher overall accuracy of the NA12878 solution is due to better variant specificity of the truth data (i.e., false variants are filtered in the truth data and therefore not included for comparison).

The above results indicate that the phasing accuracy of HapCUT2 (even considering only high-quality variant sites) is severely impacted by the presence of false variants with ambiguous linkage. In particular, the appearance of long-range switching errors (e.g., between p- and q-arms) indicates that local phasing errors can compromise the accuracy of long-range Hi-C linkage. By contrast, variant filtration embedded in mLinker enables reliable local haplotype inference and the two-tier strategy preserves the specificity of long-range Hi-C linkage. These two features are essential for preserving long-range phasing accuracy.

We further ran HapCUT2 (v1.0) for whole-chromosome haplotype inference using 11 × RPE-1 PacBio CCS data and the same Hi-C data (v.1.3.1 was giving errors on PacBio Hi-Fi data). Aligned PacBio CCS reads were converted to the fragment file format using the following command:


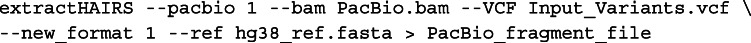


PacBio and Hi-C fragments were then merged and assembled into haplotype blocks with the following commands:


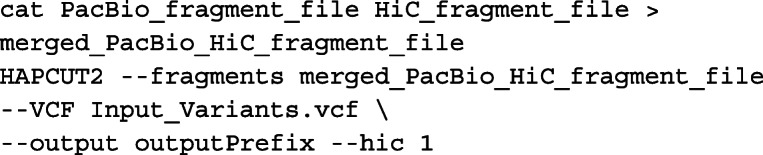


Due to the low depth of the long-read data (11 ×), we expected that ambiguous linkage evidence from false variants would be more problematic for HapCUT2 and therefore only ran HapCUT2 using high-quality variants. The solution from HapCUT2 showed similar accuracy as mLinker but contained more phased variants. However, the Chr.1 solution showed switching at the centromere with p- and q-arm haplotypes (Additional file 1:Fig. S7). By contrast, the mLinker solution derived from high-quality variants showed consistent accuracy across all chromosomes (maximum error rate 4.2% on Chr.X). Even when all variants were used as input, mLinker produced haplotypes at a similar accuracy (<4.4%) for all autosomes but not for Chr.X (∼10%) that has lower variant density. This example demonstrates the robustness of mLinker for long-range haplotype inference in contrast to HapCUT2.

## Supplementary Information

**Additional file 1** This file contains the following figures:
∙ Figure S1: Distributions of the energy penalty scores in the haplotype solution of the RPE-1 and NA12878linked-reads data
∙ Figure S2: Low variant density regions in the NA12878 genome
∙ Figure S3: Low variant density regions in the NA12878 genome and boundaries of haplotype blocks inferred from the linked-reads data
∙ Figure S4: Low variant density regions in the RPE-1 genome and boundaries of haplotype blocks inferred from the linked-reads data
∙ Figure S5: Concatenation of haplotype blocks using Hi-C links
∙ Figure S6: Block switching penalty cutoff and phasing accuracy
∙ Figure S7: Examples of switching errors in the HapCUT2 haplotype solutions
tables:
∙ Table S1: Data used for haplotype inference and karyotype reconstruction of the K-562 genome
∙ Table S2: Comparison between the scaffold haplotype solution and the reference haplotypes of NA12878
∙ Table S3: Comparison between the scaffold haplotype solution and reference haplotypes of RPE-1
∙ Table S4: Benchmark of the RPE-1 haplotype solution including variants in centromeric regions
∙ Table S5: Benchmarks of the scaffold haplotype solution from down-sampled linked-reads and Hi-C data
and sections of supplementary discussion:
∙ Linkage evidence from molecular identifier and sequence alignment of linked reads
∙ Haplotype inference and energy minimization of the 1D spin model in Eq. (14)
∙ Software implementation of the haplotype inference algorithm
∙ Comparison of phased variant genotypes with parent-specific k-mer’s
∙ Determination of the K-562 karyotype by haplotype-specific genomic analysis

**Additional file 2** This table reports the performance of mLinker solve using haplotype linkage in the RPE-1 linked reads data. For each round of minimization, the following numbers are reported: number of spin flips (column 1), number of block switches (column 2), CPU clock time of spin flipping (column 3) and block switching (column 4), and the maximum residual block-switching energy penalty (column 5). Details of the minimization procedure is provided in Additional file 1:Solving haplotype phase by minimization; spin flipping and block switching are defined in Eqs. (18) and (19).

**Additional file 3** This table summarizes additional benchmark metrics of the haplotype solution of the NA12878 genome in comparison to the haplotype phase derived from parental genome sequencing. The parental haplotype data used for comparison include the reference haplotype data released by the Genome-In-A-Bottle consortium (“trio phase”), and phased haplotypes derived from de novo diploid assembly of the NA12878 genome (“dip assembly”) using PacBio CCS reads of the NA12878 genome and short reads of the parental genomes. See Table 1 for more details about the reference haplotype data. In the 1st Tab, each row summarizes the following metrics about the haplotype solution of each chromosome (column 1): phased sites from de novo diploid assembly (column 2), phased sites in the GIAB release (column 3), phased sites in the final unfiltered haplotype solution from mLinker (column 4), phased genotypes in the mLinker solution that are in agreement with the GIAB data (column 5), mLinker phased genotypes in discordance with the GIAB data (column 6), mLinker phased genotypes in agreement with the haplotype from diploid assembly (column 7), mLinker phased genotypes in discordance with the haplotype from diploid assembly (column 8), phased sites in the final filtered haplotype solution from mLinker (column 9), mLinker phased genotypes in agreement with the GIAB data (column 10), mLinker phased genotypes in discordance with the GIAB data (column 11), mLinker phased genotypes in agreement with the haplotype from diploid assembly (column 12), mLinker phased genotypes in discordance with the haplotype from diploid assembly (column 13). The 2nd Tab reports results of the comparison of mLinker-phased genotypes on each parental chromosome with parent-specific sequences derived from the short-read sequencing data of parental genomes. For details about this comparison, see Additional file 1:Comparison of phased variant genotypes with parent-specific *k*-mer’s.

**Additional file 4** This table contains multiple tabs. Tab 1 reports results from the comparison of the mLinker haplotype solution of the RPE-1 genome generated from 60 × linked-reads and Hi-C sequencing data to the reference haplotype data derived from sequencing of monosomic chromosomes. Tabs 2-5 report results from the comparison of the mLinker haplotype resolution generated from downsampled linked-reads and Hi-C data. Tab 6 reports benchmark metrics of the mLinker haplotype solution generated from 11 × PacBio HiFi data and Hi-C data.
In Tab 1, the comparison is performed on both the scaffold haplotype solution and the final haplotype solution filtered by haplotype linkage. In Tabs 2-5, the comparison is only performed on the scaffold haplotype solution. For each mLinker solution in Tabs 1-5, we report results from the comparison of phased genotypes at all phased variant sites (“No filter”), at phased sites not in centromeric/acrocentric regions (“Excluding centromere”), and at phased sites also passing the allele fraction filter from single-cell data (“allele filter from single-cell data”). In Tab 6, we report results from two separate calculations, the first using all variants as input, the second using only high-quality variants (sites that pass the linkage filter in the mLinker final haplotype solution derived from linked-reads and Hi-C data). The comparison in Tab 6 is only performed on high-quality variants.

**Additional file 5** Please refer to Additional file 1:Determination of the K-562 karyotype by haplotype-specific genomic analysis for a detailed explanation.

**Additional file 6** Each row in this table corresponds to a monosomy (column 2) in a single cell sample (column 1). The following metrics are reported for each monosomy: mean sequencing depth of the monosomic chromosome (column 3) and across the genome (column 4), percentage of reference (column 5), alternate (column 6), and heterozygous coverage (column 7), normalized heterozygosity (column 8), SRA BioProject ID (column 9), SRR accession number (column 10). Details of data generation and analysis are provided in “Sequencing data of monosomic RPE-1 cells” section.

**Additional file 7** Please refer to Whole-chromosome haplotype inference by HapCUT2 for a detailed explanation.

**Additional file 8** Review history.

## Data Availability

The data sources are listed in Table 1 (RPE-1 and NA12878) and Additional file 1:Table S1 (K-562). The NA12878 linked-reads bulk whole-genome sequencing data are generated and provided by 10x Genomics [54]. The RPE-1 linked-reads data are available in the NCBI Short Read Archive (SRA) as SRR14077648 under BioProject PRJNA602546 [55]. The K-562 linked-reads data are generated in Ref. [50] and available in [56]. The NA12878 and K-562 Hi-C data are generated in Ref. [35] and available in the NCBI Short Read Archive (SRA) under BioProject PRJNA268125 [57]. The RPE-1 Hi-C data are generated in Ref. [44] and available in the NCBI Short Read Archive (SRA) under BioProject PRJNA292502 [58]. The RPE-1 standard bulk WGS data are available in the NCBI Short Read Archive (SRA) under BioProject PRJNA273160 [53]. The standard whole-genome sequencing data of single monosomic and aneuploid RPE-1 cells are available in the NCBI Short Read Archive under BioProjects PRJNA602546 [55] and PRJNA698413 [59]. Whole-genome sequencing of aneuploid RPE-1 populations used for generating Fig. 5 are available in the European Genome-Phenome Archive under EGAD00001001629 [60]. Two datasets of phased variants of the NA12878 genome (for validation) are generated by the Genome-In-A-Bottle consortium [61] and provided by Heng Li [62]. The main computational method is implemented as an open-source (MIT License) C++ package “mLinker” [63]. Final haplotype solutions of the NA12878 and the RPE-1 genomes are provided in the mLinker github repository. Intermediate results of all analyses are available upon request. Declarations
