# Supplementary material for: Determination of complete chromosomal haplotypes by bulk DNA sequencing
Source: Genome Biol. 2021 May 6;22:139. doi: 10.1186/s13059-021-02330-1 (PMC8101039; doi:10.1186/s13059-021-02330-1)
Supplement: Supplementary file 1 — Additional file 1 This file contains the following figures: ∙ Figure S1: Distributions of the energy penalty scores in the haplotype solution of the RPE-1 and NA12878linked-reads data ∙ Figure S2: Low variant density regions in the NA12878 genome ∙ Figure S3: Low variant density regions in the NA12878 genome and boundaries of haplotype blocks inferred from the linked-reads data ∙ Figure S4: Low variant density regions in the RPE-1 genome and boundaries of haplotype blocks inferred from the linked-reads data ∙ Figure S5: Concatenation of haplotype blocks using Hi-C links ∙ Figure S6: Block switching penalty cutoff and phasing accuracy ∙ Figure S7: Examples of switching errors in the HapCUT2 haplotype solutions tables: ∙ Table S1: Data used for haplotype inference and karyotype reconstruction of the K-562 genome ∙ Table S2: Comparison between the scaffold haplotype solution and the reference haplotypes of NA12878 ∙ Table S3: Comparison between the scaffold haplotype solution and reference haplotypes of RPE-1 ∙ Table S4: Benchmark of the RPE-1 haplotype solution including variants in centromeric regions ∙ Table S5: Benchmarks of the scaffold haplotype solution from down-sampled linked-reads and Hi-C data and sections of supplementary discussion: ∙ Linkage evidence from molecular identifier and sequence alignment of linked reads ∙ Haplotype inference and energy minimization of the 1D spin model in Eq. (14) ∙ Software implementation of the haplotype inference algorithm ∙ Comparison of phased variant genotypes with parent-specific k-mer’s ∙ Determination of the K-562 karyotype by haplotype-specific genomic analysis [file 13059_2021_2330_MOESM1_ESM.pdf]

Supplementary Figures

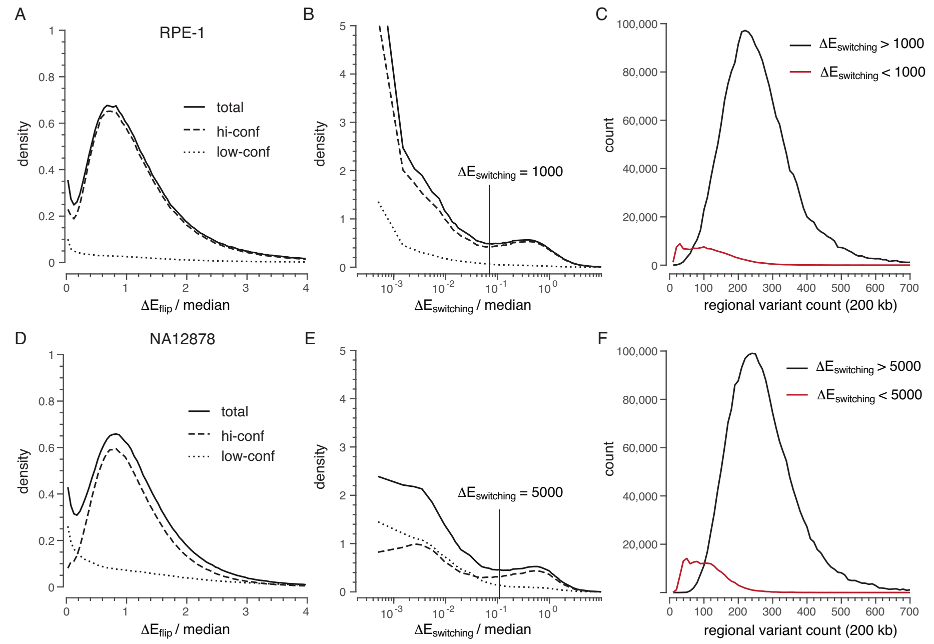

Figure S1: Distributions of the energy penalty scores in the haplotype solution of the RPE-1 (A-C) and NA12878 (D-F) linked-reads data. The energy penalty scores measure the confidence of haplotype inference with respect to single base (A and D) and block-switching (B and E) errors. For each type of phasing error, the distribution of penalty scores is shown for all variants (solid line) and separately for high-confidence variants (dashed line) and low-confidence variants (dotted line). In the distribution plots, the energy penalty is normalized by the median number of unique molecular links at each variant site. Panel C and F show the distributions of local variant density near each variant site (estimated by the number of high-confidence variants within a 200kb neighborhood) with high (black) and low (dark red) switching penalty scores in the RPE-1 genome (C) and in the NA12878 genome (F).

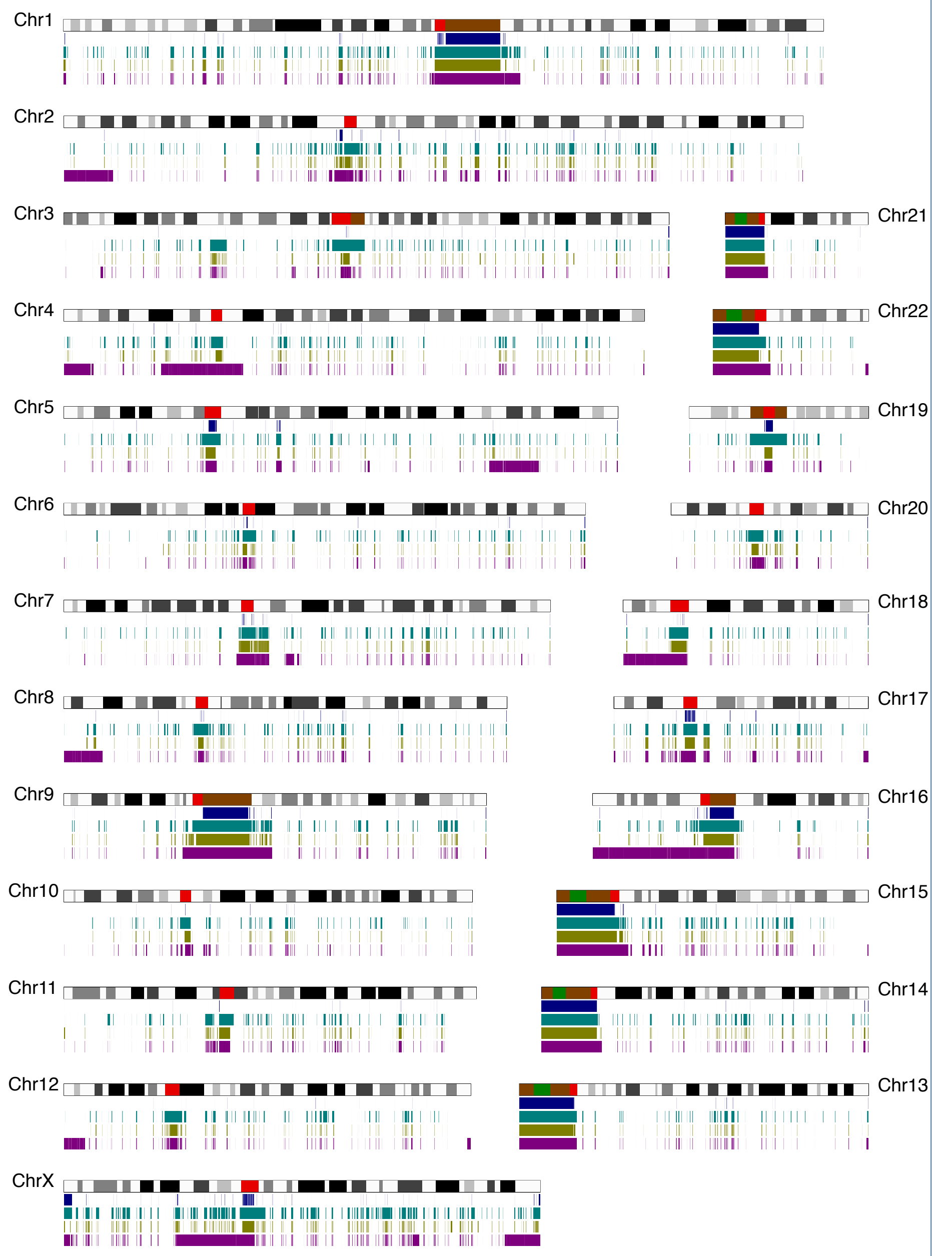

Figure S2: Low variant density regions in the NA12878 genome. Each track represents regional variant density (number of variants in 200kb bins) estimated from (from top to bottom) (1) all SNVs detected by `HaplotypeCaller` on the linked-reads data (blue); (2) phased SNVs in the final haplotype solution from `mLinker` (cyan); (3) phased SNVs from *de novo* assembly of parental chromosomes (olive); (4) phased SNVs from the parental genomes (GIAB reference) (purple). Bins with more than 20 variants (variant density more than 1 per 10 kb) are omitted.

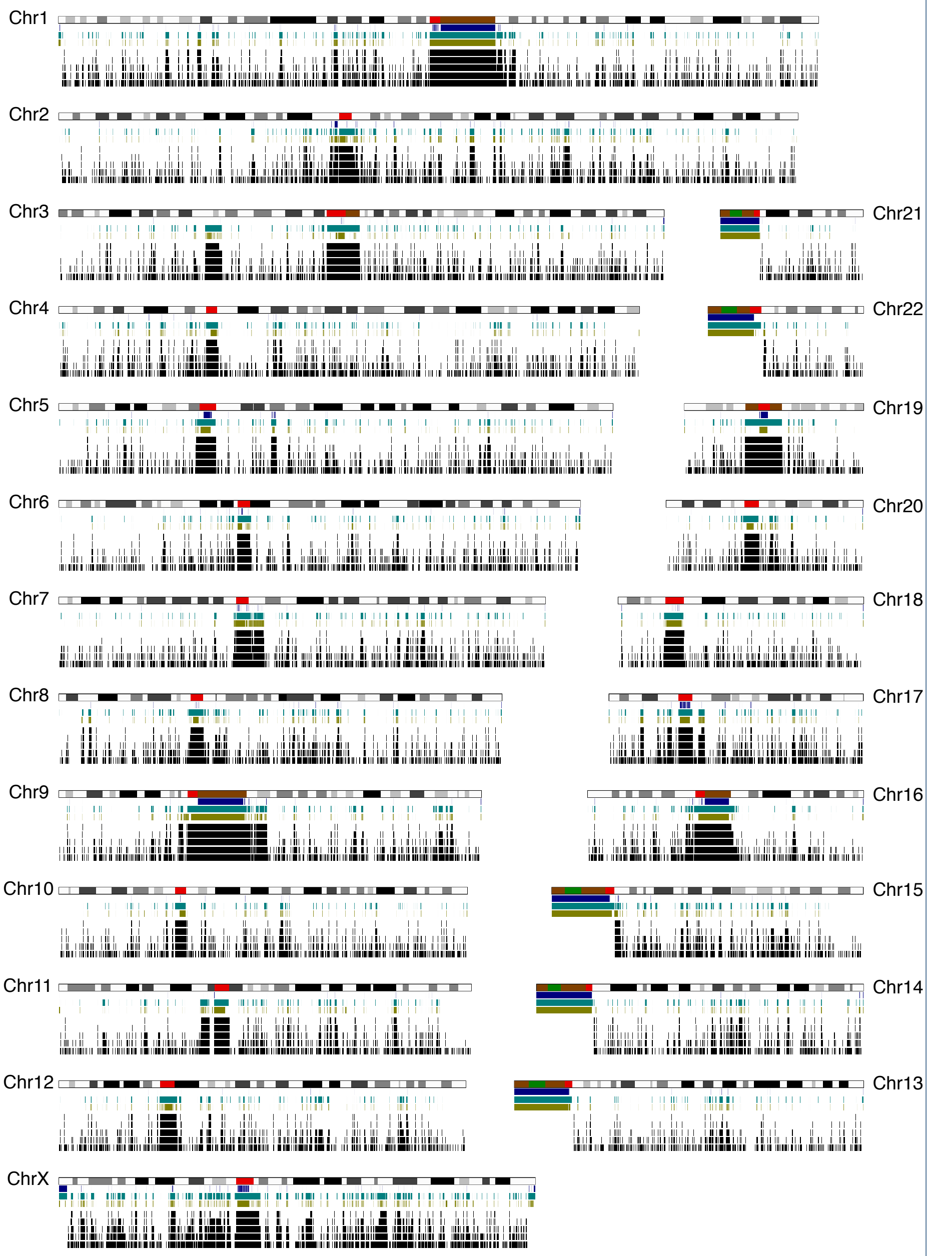

Figure S3: Low variant density regions in the NA12878 genome and boundaries of haplotype blocks inferred from the linked-reads data. Only the first three variant density tracks in Fig. S2 are shown here. The haplotype blocks are determined based on the following block-switching penalty cutoff (top to bottom): 5,20,100,500,5000. The last block-switching cutoff was used to generate high-confidence haplotype blocks in the scaffold haplotype.

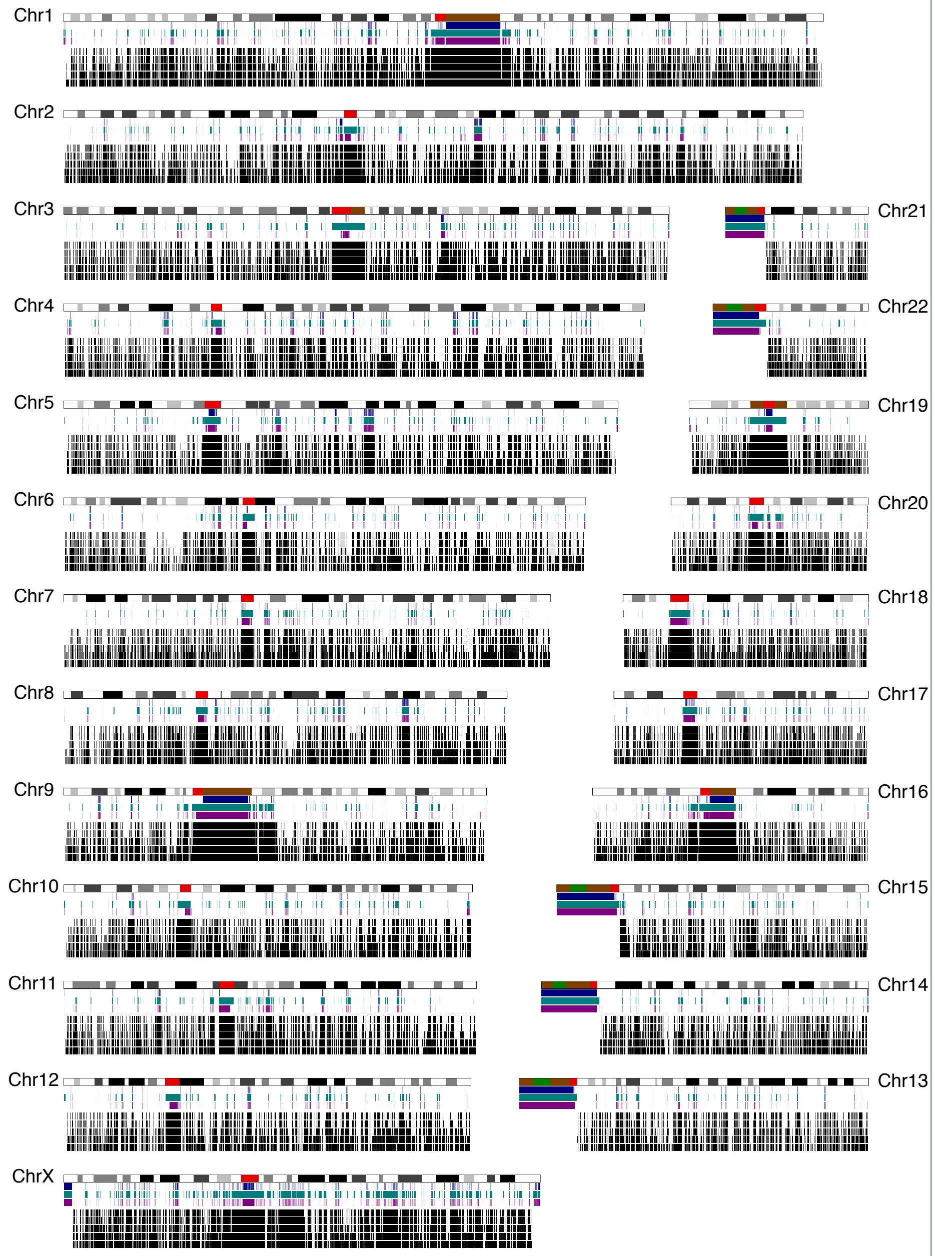

Figure S4: Low variant density regions in the RPE-1 genome and boundaries of haplotype blocks inferred from the linked-reads data. The variant density tracks represent regional variant density (number of variants in 200kb bins) estimated from (from top to bottom) (1) all SNVs detected by `HaplotypeCaller` on the linked-reads data (blue); (2) phased SNVs in the final haplotype solution from `mLinker` (cyan); (3) phased SNVs from the sequencing data of single chromosomes (purple). Bins with more than 20 variants (variant density more than 1 per 10 kb) are omitted. The haplotype blocks are determined based on the following block-switching penalty cutoff (top to bottom): 5,20,100,500,1000. The last block-switching cutoff was used to generate high-confidence haplotype blocks in the scaffold haplotype.

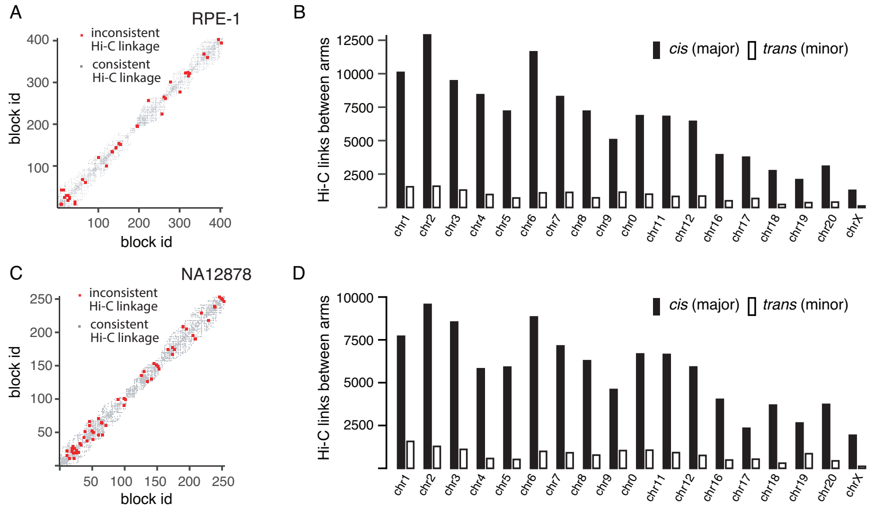

Figure S5: Concatenation of haplotype blocks using Hi-C links. A and C. Heatmaps of *cis* (gray) and *trans* (red) Hi-C linkage between local haplotype blocks of Chr.X determined from the RPE-1 (A) and the NA12878 data (C). The centromere is located near block 200 in the RPE-1 solution and near block 100 in the NA12878 solution. B and D. Total number of major (*cis*) and minor (*trans*) Hi-C links between p- and q-arms in the RPE-1 (B) and the NA12878 data (D).

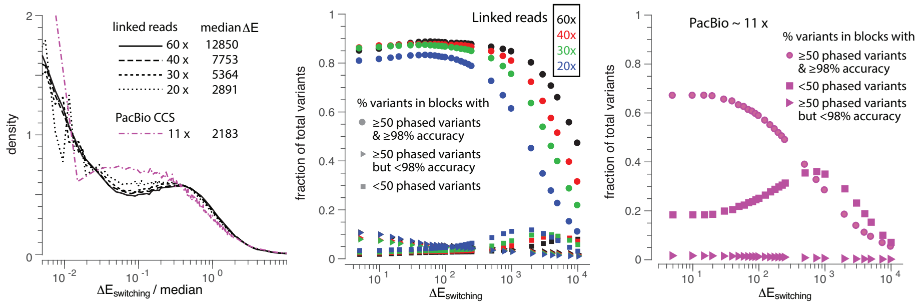

Figure S6: Block switching penalty cutoff and phasing accuracy. A. Distributions of switching penalty scores in the local haplotype solution of linked-reads (60×, 40×, 30×, 20×) and PacBio Circular-Consensus Sequencing data (11×). The switching penalty in the haplotype solution from linked-reads data always shows a minimum around 0.1×median that can be used to determine high-confidence haplotype blocks. The haplotype solution using PacBio reads does not show this feature. B and C. Fraction of phased variants in long (≥50 phased variants) phase blocks with ≥98% accuracy (squares), in long phase blocks with <98% accuracy (triangles), and in short phase blocks (circles) in the haplotype solution derived from linked-reads (B) and PacBio CCS data (C). Variants not in any phase block are excluded. Based on the fraction of variants in long blocks with high accuracy, the optimal switching energy cutoff for the linked-reads data is approximately 100; for the PacBio solution derived using only high-quality variants, the optimal cutoff is between 5 and 10. The better accuracy of the PacBio haplotype solution reflects two advantages of PacBio Hi-Fi reads over short linked reads: better alignment accuracy and more specific haplotype linkage. These advantages make the estimation of switching errors more accurate.

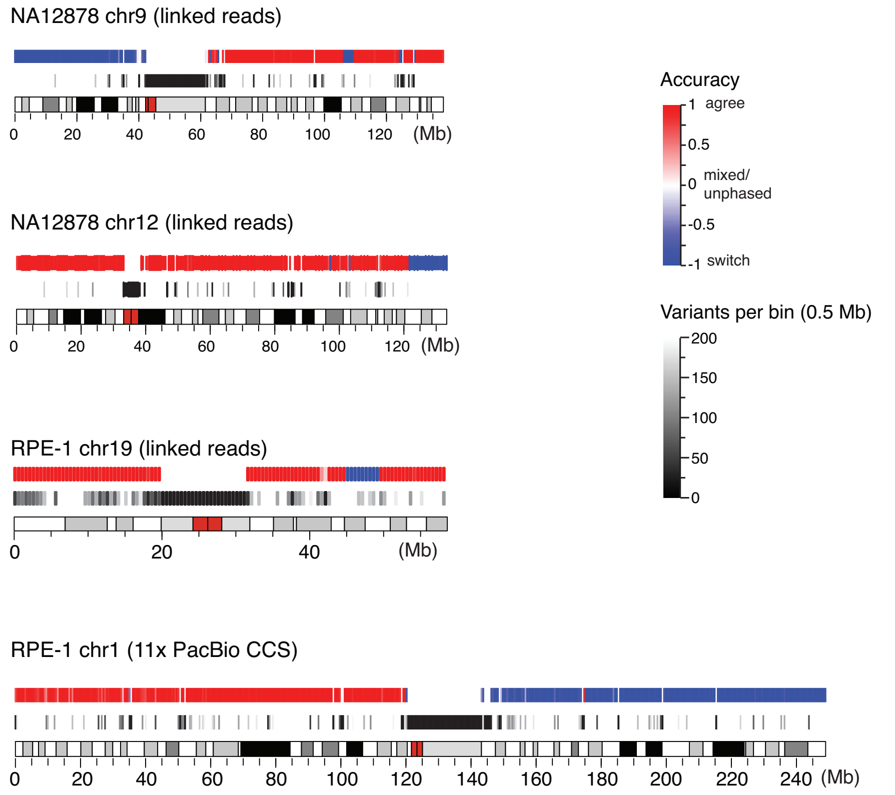

Figure S7: Examples of long-range switching errors in the HapCUT2 haplotype solutions. The first three examples (Chr.9 and Chr.12 from NA12878, Chr.19 from RPE-1) show mixed parental haplotypes (red and blue) in the HapCUT2 solution generated from ~60× linked-reads and Hi-C data with unfiltered heterozygous variants as input. The bottom example (Chr.1 from RPE-1) shows mixed parental haplotypes in the HapCUT2 solution generated from 11× PacBio CCS data and Hi-C data using only high-quality variants. Local phasing accuracy is represented by the percentage of correctly phased genotypes in 0.5Mb bins relative to the truth haplotype. Red color shows bins with haplotype phase in agreement with the major parental haplotype (~100% accuracy), blue color represents bins with the opposite (minor) parental haplotype (~-100%). Switching between parental haplotypes occurs at the boundaries between red and blue haplotypes.

Supplementary Tables

Table S1: Data used for haplotype inference and karyotype reconstruction of the K-562 genome

Sample	Data type	Data source	Read count	Mean depth	Application
K-562	linked reads	Zhou et al. (2019) [49]	736,246,361[a]	60×[b]	local phasing
K-562	Hi-C	Rao et al. (2014) [35]	456,757,799[c]<br>591,854,553[d]		long-range phasing, karyotype construction

[a] https://www.encodeproject.org/experiments/ENCSR053AXS/
Mean Molecular length 58.2 kb; median insert 385; 661,037,851 aligned in pair; 2 × 151bp; duplication rate 0.012.

[b] excluding the GEMcode sequence

[c] SRR1658693: median insert 357; 428,963,615 aligned in pair; 2 × 101bp; duplication rate 0.05.

[d] SRR1658694: median insert 341; 578,201,812 aligned in pair; 2 × 101bp; duplication rate 0.17.

Table S2: Comparison between the scaffold haplotype solution and reference haplotypes of NA12878

ref_id	Total variant sites[a]	Sites for phasing[b]	Scaffold haplotype[c]	Comparable to GIAB	Agreement with GIAB	Comparable to assembly	Agreement with assembly
chr1	193,281	176,458	160,578	137,172	0.995	149,740	0.997
chr2	208,196	189,810	174,664	139,562	0.999	163,572	0.999
chr3	175,998	155,662	145,153	126,777	0.999	135,682	0.997
chr4	179,803	163,757	153,465	110,687	0.999	145,358	0.999
chr5	164.837	151,544	142,076	115,291	0.999	135,066	0.998
chr6	174,820	161,825	152,261	126,250	0.986	137,804	0.999
chr7	147,495	136,008	124,499	102,500	0.996	114,419	0.998
chr8	136,150	126,479	117,763	90,290	0.999	111,478	0.997
chr9	119,635	109,185	99,223	80,150	0.999	87,186	0.998
chr10	133,655	119,667	111,518	91,867	0.992	103,515	0.992
chr11	125,208	111,748	103,244	87,692	0.999	95,878	0.999
chr12	120,828	110,074	102,101	80,984	0.999	95,120	0.998
chr13	89,785	83,292	78,981	69,059	0.999	74,978	0.999
chr14	81,921	76,081	70,468	60,787	0.999	65,586	0.999
chr15	72,770	64,540	58,841	49,344	0.999	55,519	0.999
chr16	80,402	73,735	67,472	35,018	0.994	63,327	0.993
chr17	68,924	61,140	54,597	42,054	0.989	48,718	0.992
chr18	73,239	66,670	62,328	41,705	0.999	58,838	0.999
chr19	63,856	46,748	41,658	30,635	0.985	36,830	0.983
chr20	68,144	53,616	49,585	40,104	0.998	44,705	0.996
chr21	44,623	32,979	31,183	25,818	0.999	29,162	0.999
chr22	44,187	33,498	30,971	23,024	0.990	28,249	0.999
chrX	84,624	74,634	61,529	45,244	0.999	56,863	0.999
Total	2,652,381	2,379,150	2,194,158	1,746,304	0.997	2,037,593	0.997

[a] bi-allelic (one reference plus one alternate) single-nucleotide variants emitted by HaplotypeCaller

[b] excluding sites in centromeric regions or not having linkage to both reference and alternate genotypes

[c] concatenation of local haplotype blocks (solved from linked reads) by long-range Hi-C links

Table S3: Comparison between the scaffold haplotype solution and reference haplotypes of RPE-1

ref_id	Total variant sites[a]	Phased from monosomies[b]	Sites for phasing[c]	Scaffold haplotype[d]	Comparable sites	Agreed	Fraction of agreement
chr1	185,997	179,216	176,078	163,057	159,408	157,056	0.985
chr2	194,641	188,297	186,201	175,383	172,093	170,210	0.989
chr3	169,194	157,887	152,824	145,109	137,854	135,905	0.986
chr4	168,626	161,562	161,915	154,564	150,795	148,588	0.985
chr5	149,212	146,447	142,975	135,587	134,138	132,877	0.991
chr6	156,723	155,379	149,049	143,371	142,649	141,478	0.992
chr7	143,405	139,365	136,858	129,164	126,868	123,263	0.972
chr8	124,489	120,140	120,905	114,559	111,458	110,321	0.990
chr9	115,189	110,993	108,636	99,516	97,845	94,032	0.961
chr10	119,524	112,758	112,078	106,059	102,486	100,767	0.983
chr11	116,321	113,375	107,966	102,255	101,091	98,782	0.977
chr12	111,225	106,331	105,314	99,033	96,115	94,095	0.979
chr13	84,586	83,092	82,270	78,835	77,752	77,059	0.991
chr14	74,463	71,195	71,637	67,289	65,023	64,021	0.985
chr15	70,936	62,662	66,134	61,948	55,646	54,013	0.971
chr16	78,049	70,375	72,948	67,658	62,217	61,154	0.983
chr17	75,407	60,418	69,001	64,261	52,385	50,393	0.962
chr18	64,006	61,622	60,708	57,620	57,037	56,692	0.994
chr19	61,461	26,906[e]	44,083	41,246	18,402	18,023	0.979
chr20	61,029	55,063	49,742	46,735	43,738	42,999	0.983
chr21	44,178	36,240	33,089	31,755	28,079	27,885	0.993
chr22	40,635	36,154	30,115	28,140	26,142	25,802	0.987
chrX	66,015	64,676	62,476	52,503	51,926	51,441	0.991
Total	2,475,311	2,320,153	2,303,002	2,165,647	2,071,147	2,036,856	0.983

[a] bi-allelic (one reference plus one alternate) single-nucleotide variants emitted by HaplotypeCaller
[b] reference haplotypes determined from whole-genome sequencing of single monosomic RPE-1 cells
[c] excluding sites in centromeric regions or not having linkage to both reference and alternate genotypes
[d] concatenation of local haplotype blocks (solved from linked reads) by long-range Hi-C links
[e] generated from one monosomic cell and having a lower percentage of phased variants

Table S4: Benchmark of the RPE-1 haplotype solution including variants in centromeric regions

	Phased from bulk data	Comparable sites	Agreed	Accuracy
Scaffold solution	2,219,275	2,109,626	2,067,657	0.980
Scaffold solution excluding centromeric variants	2,160,500	2,066,502	2,034,188	0.984
Scaffold solution with allele filter	2,051,146	1,992,685	1,981,142	0.994
Final solution[a]	2,197,578	2,102,419	2,082,465	0.991
Final solution excluding centromeric variants	2,155,100	2,070,307	2,054,230	0.992
Final solution with allele filter	2,083,478	2,024,997	2,016,935	0.996

[a] determined from haplotype linkage to the scaffold solution, with linkage filter specified as in Table 3

Table S5: Benchmark of the scaffold haplotype solution from down-sampled linked-reads and Hi-C data

Completeness[a]	all links from linked-reads	66% links from linked-reads	50% links from linked-reads	33% links from linked-reads
all Hi-C links	1	0.988	0.981	0.955
66% Hi-C links	0.997	0.984	0.976	0.950
50% Hi-C links	0.992	0.980	0.971	0.944
33% Hi-C links	0.984	0.970	0.961	0.931
**Accuracy[b]**	**all links from linked-reads**	**66% links from linked-reads**	**50% links from linked-reads**	**33% links from linked-reads**
all Hi-C links	0.994	0.993	0.993	0.992
66% Hi-C links	0.993	0.993	0.993	0.992
50% Hi-C links	0.991	0.992	0.992	0.991
33% Hi-C links	0.992	0.985[c]	0.990[c]	0.988[c]

[a] measured against the scaffold solution derived from all linked-reads and Hi-C linkage
[b] measured by comparison to the haplotype solution determined from monosomic cells at variant sites that pass the single-cell allele fraction filter (see Table 3)
[c] The haplotype phase of Chr.X shows <90% global accuracy. See Additional file 4.

Supplementary Discussion

Linkage evidence from molecular identifier and sequence alignment of linked reads

Molecular linkage between linked reads is reflected in two features. First, fragments derived from the same DNA molecule should share the same molecular barcode:

$$\text{physical linkage} \Rightarrow \text{identical molecular barcode.}$$

Second, fragments derived from the same DNA molecule should map to proximal locations based on their sequence content:

$$\text{physical linkage} + \text{correct alignment} \Rightarrow \text{proximity of alignment positions.}$$

The logic behind barcode-aware aligners (*e.g.*, Lariat) [34] is the following :

$$\left.\begin{array}{r}\text{identical molecular barcode} \dashrightarrow \text{physical linkage}\\ \text{sequence information}\end{array}\right] \rightsquigarrow \text{optimal alignment positions.}$$

This strategy can improve the placement of sequence fragments with multiple possible alignment positions using the alignment positions of **uniquely aligned fragments** with the same molecular barcode. For example, the alignment positions of sequence fragments derived from short interspersed repeats (typically <10kb) may be anchored by the alignment positions of fragments in the flanking non-repeat regions. However, for sequence fragments derived from regions of segmental duplications, or from regions not represented in the reference genome (*e.g.*, centromeres), their true alignment positions cannot be uniquely determined or are not included in the reference. In these scenarios, barcode-aware aligners may reinforce false linkage evidence if these fragments are placed in proximity but at incorrect locations.

To ensure **the best specificity of linkage between sequence fragments**, we want to use molecular barcodes and alignment positions as independent evidence of linkage between sequence fragments. The alignment positions are determined solely based on the DNA sequence and do not use the molecular barcode information (*i.e.*, barcode-agnostic alignment). We consider sequence fragments to be linked in *cis* only when they both share the same molecular barcode and are aligned to proximal positions (< 100 kb) independent of the molecular barcodes:

$$\left.\begin{array}{r}\text{identical molecular barcode}\\ \text{proximity of alignment positions } (< 100\text{ kb})\end{array}\right] \Rightarrow \textit{cis} \text{ linkage between fragments.}$$

The 100kb threshold is determined from the distance feature of linkage density and linkage accuracy in the linked-reads data (Fig. 2).

For sequence fragments derived from "difficult" regions such as segmental duplications, a barcode-agnostic aligner will place them at random locations (if multiple hits are found) and/or with low mapping quality scores. Linkage evidence from these ambiguously aligned fragments will be excluded based on the distance filter (100kb) or by the mapping quality filter (see Extracting variant linkage information from long-range sequencing). The exclusion of ambiguous linkage evidence due to alignment inaccuracy ensures better accuracy of linkage evidence that is appropriate for **haplotype inference**.

For *de novo* variant discovery, especially of **structural variants**, the benefit of improved alignment accuracy using barcode-aware alignment outweighs the compromise of linkage accuracy. For such applications, using barcode-aware alignment may be advantageous provided that the contributions to the mapping quality score from sequence alignment and from the molecular barcode can be accurately calibrated.

Software implementation of the haplotype inference algorithm

In this section we describe the different modules of `mLinker` related to haplotype inference.

Extracting variant linkage information from long-range sequencing

Taking long-range sequencing data and a set of heterozygous variants as input, "`mlinker extract`" generates a hash map from variant genotypes to sequencing reads ("variant-to-read") by iterating over all sequencing reads at each variant site. This module can be executed either on an entire chromosome or on specified regions of interest. The output are plain text files with each line listing the names/identifiers of sequencing reads showing a specific variant genotype. For example,

```
198801_A_C -> {read_identifier1, read_identifier2, read_identifier3};
```

represents the following

```
198801: variant position;
A: reference base;
C: genotype observed in the supporting reads (read_identifier1, etc.).
```

For linked-reads data, `mlinker` uses the 16-bp molecular barcodes (usually stored in the "BX" Tag) as read identifiers. For Hi-C data, `mlinker` uses read names as read identifiers. For long-read data, the default read identifier is constructed by concatenating the read name with the start and end positions of an alignment; this definition ensures that only linkage between variants within a single contiguous alignment is preserved, but not between variants in non-contiguous (split) alignments of the same read. `mLinker` additionally applies the following read filters (to be customizable in a future release):

Data type	Mapping quality	Base Quality	BAM Flag
linked-reads	< 20	< 20	duplicate, non-primary
long-read	< 20	< 8[a]	duplicate, non-primary
Hi-C	< 20	< 20	duplicate

[a] This threshold applies to uncorrected PacBio reads but not to corrected PacBio reads.

As `mLinker` iterates over all variants to generate the variant-to-read map, it simultaneously creates the inverse map from reads to linked variants in a separate text file. Each line in the "read-to-variant" file lists all the variant genotypes linked by each read/molecule. For example,

```
read_identifier1 -> {198801_A_C, 198322_T_T, 196990_C_G};
```

indicates that the `C`, `T`, and `G` bases are observed at positions 198801, 198322, and 196990 in a single read or linked-read molecule with identifier `read_identifier1`. The variant-to-read and the read-to-variant maps are the only input data for downstream analysis.

Calculating haplotype-specific linkage between variants

The signal of haplotype linkage between two variant genotypes (*e.g.*, `198801_A_C` and `198322_T_G`) is measured by the number of unique reads (molecules) linking these genotypes, which is calculated by intersecting the list of reads associated with each variant genotype (using the variant-to-read map). The calculation of haplotype linkage is embedded in the "`mlinker solve`" module prior to haplotype solution. We have implemented a separate module"`mlinker matrix`" that calculates haplotype linkage between variants on each chromosome.

Solving haplotype phase by minimization

`mlinker solve` takes the variant-to-read and the read-to-variant hash maps as input and finds the optimal haplotype solution by alternately performing spin flipping or block switching to lower the energy function given by Eq. (14). During each round of minimization, the program first performs spin flips at sites with negative flipping energy penalties calculated using Eq. (15); it then performs block switches between sites with negative switching energy penalties calculated using Eq. (16).

In calculating the block switching energy $\Delta E_{k|k+1}$, we have taken advantage of the following recursive relationship

$$\begin{aligned}\Delta E_{k|k+1} - \Delta E_{k-1|k} &= \sum_{i\le k}\sum_{j>k} M_{ij}s_is_j - \sum_{i<k}\sum_{j\ge k} M_{ij}s_is_j \\ &= s_k\left(\sum_{j>k} M_{kj}s_j - \sum_{i<k} M_{ik}s_i\right) \\ &= s_k\left(\sum_{i>k} M_{ki}s_i - \sum_{i<k} M_{ki}s_i\right).\end{aligned} \tag{S1}$$

The last step uses the symmetric property of $M_{ik} = M_{ki}$. With the introduction of

$$h_i^+ = \sum_{j>i} M_{ij}s_j \quad \text{and} \quad h_i^- = \sum_{j<i} M_{ij}s_j, \tag{S2}$$

the spin flipping energy ΔE_i and the block switching energy $\Delta E_{k|k+1}$ are given by

$$\Delta E_i = s_i\left(h_i^+ + h_i^-\right) \quad \text{and} \quad \Delta E_{i|i+1} = \Delta E_{i-1|i} + s_i\left(h_i^+ - h_i^-\right). \tag{S3}$$

Therefore, the calculation of both energy penalties over all variant sites has the same complexity as the calculation of h_i^+ and h_i^-, which is proportional to the total number of non-zero M_{ij}'s that is approximately given by the total number of variants (N) multiplied by the average number of variants linked by each molecule (L).

The energy penalties are calculated iteratively from the first spin to the last using Eqs. (S2) and (S3) while the spin configuration is continuously updated. This asynchronous iteration scheme achieves faster convergence to an energy minimum than synchronous iteration in which all spins are updated at once. The difference between these two iteration strategies is illustrated using a toy example of spin flipping.

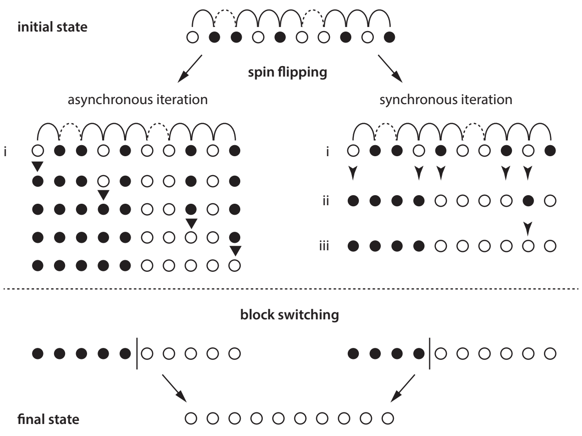

In this example, we start from a random initial configuration ($s_i^{(0)} = \pm 1$) with open and filled circles representing genotypes in complementary haplotypes. The interactions between adjacent genotypes are shown as arcs above the haplotype configuration. The interaction is positive (dashed arc) between genotypes in *cis* and negative (solid arc) between genotypes in *trans*. Spin flips are introduced if the net interaction with adjacent spins is negative. In asynchronous iteration (left), the energy penalty is calculated as spin flips are introduced: In one round of iteration, the 1st, 4th, 8th, and 10th spins are flipped due to negative interactions with the neighbor spins. In synchronous iteration (right), the 5th and the 9th spins are also flipped due to negative interactions with their neighbors in the initial state (ii); it will take another round of iteration to reverse the 9th spin to the optimal configuration (iii). The final configurations from both iterations show a single switching between haplotype blocks that can only be resolved by block switching moves.

Concatenating haplotype blocks using Hi-C links

This calculation is implemented `mlinker scaffold` and involves multiple steps. It first determines high-confidence haplotype blocks from the haplotype solution generated by `mlinker solve` based on the block-switching penalty cutoff (default value $\Delta E = 700$) specified as an input parameter. The program then constructs maps between variants and haplotype blocks as

```
198801 -> haplotype_block_1;
198322 -> haplotype_block_1;
haplotype_block_1 -> {198801, 198322, ...};
haplotype_block_2 -> {199005, 200142, ...}.
```

Phased genotypes within each haplotype block are represented as (1 for reference and -1 for alternate):

```
haplotype[haplotype_block_1]=[1, -1, -1, ...].
```

The phased linkage between haplotype blocks is calculated by iterating over all Hi-C reads spanning variants (generated by `mlinker extract`) in different blocks using Eqs. (19) and (20).

When concatenating haplotype blocks, `mlinker scaffold` first uses Hi-C links between variants within 10Mb to solve the phase of haplotype blocks (cf. Eq. (12)) within each chromosome arm using the same strategy as described in Solving haplotype phase by minimization. In this step, it also drops short haplotype blocks with ≤5 total Hi-C links to other blocks to expedite convergence. The p- and q-arm haplotypes are then joined by evaluating all phased Hi-C links between the two arms. If the haplotypes of both arms are solved correctly, then the Hi-C linkage between arms should show a strong bias (>10:1) towards *cis* (intra-chromosomal) linkage (Fig. S5). The output of `mlinker scaffold` is referred to as the "scaffold haplotype solution".

Calculation of haplotype linkage between individual genotypes and the scaffold haplotype solution

We have implemented "`mlinker recover`"to phase individual variant genotypes directly by their linkage to phased variants in the scaffold haplotype. (Currently this only uses linkage from the linked-reads data between variants within 100kb. It is straightforward to incorporate long-range Hi-C linkage but further optimization is needed to integrate both types of linkage evidence.) The linkage between a variant genotype (*e.g.*, `198801_A_C`) and a scaffold haplotype solution (**S**) can be calculated in two ways. The first definition uses the sum of linkage evidence between the variant genotype of interest and each phased genotype in the scaffold haplotype solution: this is equivalent to the spin flipping energy (Eq. (18)). Its main shortcoming is that read identifiers covering more than two variant sites in the haplotype solution are counted more than once. The second definition of haplotype linkage uses the number of unique molecules linking the variant genotype to all phased genotypes within 100kb in the scaffold haplotype solution. We use the second definition of haplotype linkage to determine the final haplotype solution and filter false variant sites with ambiguous linkage evidence. The haplotype linkage is calculated by intersecting the list of read identifiers showing a variant genotype of interest (reference or alternate) with the union of read identifiers showing genotypes from either scaffold haplotype (A or B); read identifiers containing genotypes from both parental haplotypes are excluded.

Phasing of indel variants using haplotype linkage

The `mlinker recover` module can also be used to determine the haplotype phase of indel or structural variants using the linkage of variant genotypes to the SNV haplotype. It needs a variant-to-read map that includes both phased SNV variants and unphased variants. To improve genotyping accuracy of indel variants, we have implemented an indel genotyping module based on the k-mer's of reference and alternate alleles. We first construct unique 20-mer's of reference and alternate alleles at each variant site, starting with 10-bp 5'-flanking sequence and 3'-flanking sequence taken from the reference sequence. The padding reference sequence on the 3'-end in the alternate 20-mer is adjusted to match the length of the reference 20-mer. If the reference and alternate 20-mer's are identical because the variation reflects shrinkage or expansion of homopolymers or microsatellite repeats, we incrementally increase the length of flanking sequences until the reference and the alternate k-mer's differ. For each read overlapping the variant site, we compare the subsequence taken from read to the reference and alternate k-mer's to decide the genotype supported by the read. Reads that do not extend beyond the 3'-end of the variant k-mer's are discarded.

Comparison of phased variant genotypes with parent-specific k-mer's

In addition to validating the accuracy of phased genotypes relative to the reference genome, we further tested the accuracy of parental haplotype inference by directly comparing the sequences of parental chromosomes constructed from phased genotypes and the sequences of parental genomes of NA12878. As each parental chromosome originates from a single parent, unique sequences from each parental chromosome should also be unique to a single parental genome (either mother or father). To apply this strategy, we used `yak` (https://github.com/lh3/yak) to compare unique k-mer's in

the parental chromosomes against parent-specific k-mer's extracted from the parental genomes of NA12878 (Father: NA12891; Mother: NA12892). Parent-specific 31-mer's of the NA12891 and NA12892 genomes were provided by Heng Li.

We first generated artificial parental chromosome sequences using the reference sequence and phased genotypes on each chromosome by the following commands:

```
gatk FastaAlternateReferenceMaker \
   -R hg38_ref.fa \
   -O Hap_A.fasta \
   -V Hap_A_Variants.vcf
gatk FastaAlternateReferenceMaker \
   -R hg38_ref.fa \
   -O Hap_B.fasta \
   -V Hap_B_Variants.vcf
```

We then interrogated how many parent-specific 31-mer's match with subsequences of each artificial parental chromosome using the following commands:

```
yak trioeval -t16 NA12891-pat.PG.k31.yak NA12892-mat.PG.k31.yak Hap_A.fasta
yak trioeval -t16 NA12891-pat.PG.k31.yak NA12892-mat.PG.k31.yak Hap_B.fasta
```

Results from this analysis is presented in Additional file 3:Tab 2 (Table 2). For each chromosome, `yak` identifies unique 31-mer's from both paternal ("pat vars", column 1) and maternal genomes ("mat var", column 2) that exactly match subsequences in the artificial chromosome (shown are results of one homolog). The Hamming error rate (column 4) reflects the smaller number of 31-mer's from both parental genomes relative to the total number of parent-specific 31-mer's. The switch error rate measures the frequency of switching between adjacent matching parental 31-mer's and is less relevant for global phasing accuracy. For both the reference haplotype (Genome-In-A-Bottle release) and the `mLinker` solution, we see a dominance of 31-mer's from one parent, but also a significant fraction of 31-mer's from the opposite parent.

To better understand this result, we consider different genotype combinations in the trio genomes and their implications for unique k-mer matching. Shown on the right are five representative examples of heterozygous variants in the child's genome with different genotypes in the parents. The first three variants are phased SNV variants; the fourth and fifth variants are unphased deletion variants (dashed squares). The parental chromosomes in the child's genome are shown in black with overlaid genotypes; the complementary homologs in the parents' genomes are shown in gray; k-mer's near each variant genotype (short lines) are colored according to their identity (red for maternal, blue for paternal). For phased SNV variants, variant k-mer's on parental chromosomes will match unique k-mer's in one or both parents as long as either parent shows a homozygous genotype; when both parents are heterozygous, there is no unique k-mer from either parent at this variant site (and therefore no matching to phased genotypes in the child's genome). For unphased variants, the reference sequence is used

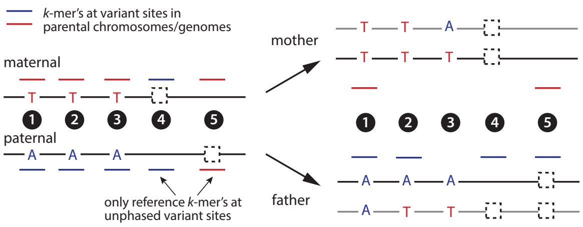

	child	mother	father	k-mer matching
1	phased	homozygous	homozygous	maternal > mother; paternal > father
2	phased	homozygous	heterozygous	paternal > father
3	phased	heterozygous	heterozygous	none
4	unphased	homozygous var	heterozygous	maternal > father; paternal > father
5	unphased	homozygous ref	homozygous var	maternal >mother; paternal > mother

for both parental chromosomes: if either parent shows the homozygous variant genotype, then the reference k-mer from both parental chromosomes will match with the parent with at least one reference allele, causing mis-assignment of the parental origin (highlighted in red in "k-mer matching"). Therefore, unphased variants (including insertion/deletion events and structural variants) may contribute a large fraction of incorrectly matched parental k-mers.

As yak does not output the positions of matching parental k-mer's on the artificial parental chromosome sequence, we cannot directly validate the above explanation. We instead performed a similar analysis to verify the parental origin of phased SNV genotypes only. We generated 35-mer's near high-quality phased variants on each chromosome and concatenated them into artificial parental chromosomes as follows:

```
A_positions=(‘bcftools query -f ‘‘%POS \n’’ Hap_A_Variants.vcf‘)
a=$(echo ‘‘$A_positions[0]-17’’|bc)
b=$(echo ‘‘$A_positions[0]+17’’|bc)
samtools faidx <Hap_A.fasta> 1:$a-$b > Hap_A_35.fa
for i in $(seq 1 ${#A_positions[@]});
do
        a=$(echo ‘‘A_positions[i]-17|bc’’)
        b=$(echo ‘‘A_positions[i]+17|bc’’)
        samtools faidx Hap_A.fasta 1:$a-$b >> Hap_A_35.fa
done
B_positions=(‘bcftools query -f ‘‘%POS \n’’ Hap_B_Variants.vcf‘)
for i in $(seq 1 ${#B_positions[@]});
do
        a=$(echo ‘‘B_positions[i]-17|bc’’)
        b=$(echo ‘‘B_positions[i]+17|bc’’)
        samtools faidx Hap_A.fasta 1:$a-$b >> Hap_B_35.fa
done
```

The error rates of phased SNV variants were then estimated by:

```
yak trioeval -t16 NA12891-pat.PG.k31.yak NA12892-mat.PG.k31.yak Hap_A_35.fasta
yak trioeval -t16 NA12891-pat.PG.k31.yak NA12892-mat.PG.k31.yak Hap_B_35.fasta
```

The results are shown in Additional file 3:Tab 2 (Table 1). In this table, Columns 2 and 3 show the total number of variants with k-mer's from both parental chromosomes matching to k-mer's from opposite parental genomes and the percentage of correct assignment; Columns 4 and 5 show the total number of variants with k-mer's from only one parental chromosome matching to a k-mer in one parental genome and the percentage of correct assignment; Columns 6 and 7 represent the total number of variants with k-mer's from either parental chromosome matching to either parental chromosome and the percentage of correct matching. The percentage of correctly phased SNV variants by this comparison (Column 7) is comparable to results derived from the truth haplotype data (Table 2 and Additional file 3:Tab 1).

About 25-32% of variants on autosomes have no matching 31-mer's in parental genomes. We think a large fraction of these variants are heterozygous in both parents. Chromosome X is special as the paternal X is the only one present in the father's genome (hemizygous). Therefore, all variant-derived k-mer's on the maternal chromosome should match k-mer's in the mother's genome. Indeed, 27,116 of 29,129 variant k-mer's match only to the maternal genome in contrast to 2,013 that match only to the paternal genome. Moreover, matching of k-mer's on the paternal X with the same X chromosome in the paternal genome implies that the maternal genome must be homozygous at the sites where these k-mer's are derived. This implies that the ratio of

variants on Chr.X with both alleles matching to opposite parental genomes (22,271) relative to those with only the maternal allele matching to the mother genome (27,116) should reflect the ratio of homozygous genotypes to heterozygous genotypes on Chr.X in the mother's genome (22,271/27,116=0.82). If we assume the same ratio for autosomes, then we estimate that 55% of heterozygous variants in the child's genome are heterozygous in the mother's genome. If we further assume a similar ratio in the father's genome, then the percentage of heterozygous variants in the child's genome that is also heterozygous in both parents is estimated to be $0.55^2 = 0.30$. This number is comparable to the percentage of variants with no matching parent-specific k-mer's and rules out the possibility that these variants are due to false detection.

Together, these results provide an independent validation of the accuracy of haplotype inference using short reads aligned to the reference genome.

Haplotype inference and energy minimization of the 1D spin model in Eq. (14)

To better understand the minimization strategy of haplotype inference, we introduce a different numerical representation of the haplotype solution using parental haplotypes $\mathbf{H}_A$ and $\mathbf{H}_B = \overline{\mathbf{H}_A}$ instead of reference and alternate genotypes. We can represent the parental haplotype $\mathbf{H}_A$ as

$$h_{A,i} = \begin{cases} 1 & \text{if haplotype A has the reference genotype at site } i \text{ ,} \\ -1 & \text{if haplotype A has the alternate genotype at site } i \text{ .} \end{cases}$$

A numerical haplotype based on the reference/alternate representation $\mathbf{S}$ can be converted to $\mathbf{S}'$ in the paternal/maternal representation as

$$\mathbf{S} = \mathbf{S}' \cdot \mathbf{H}_A, \tag{S4}$$

where

$$s'_i = \begin{cases} 1 & \text{if the genotype at site } i \text{ agrees with haplotype A,} \\ -1 & \text{if the genotype at site } i \text{ agrees with haplotype B.} \end{cases}$$

Using this new representation, we can rewrite the coupling term in Eq. (14) as

$$M_{ij} s_i s_j = M_{ij} s'_i h_{A,i} s'_j h_{A,j} = M'_{ij} s'_i s'_j. \tag{S5}$$

with

$$M'_{ij} = M_{ij} h_{A,i} h_{A,j}. \tag{S6}$$

As $h_{B,i} = -h_{A,i}$, we also have

$$M'_{ij} = M_{ij} h_{B,i} h_{B,j}. \tag{S7}$$

It is straightforward to verify that M'_{ij} is proportional to the difference between *cis* (A-A/B-B) linkage (μ_{ij}) and *trans* (A-B/B-A) linkage (δ_{ij}):

$$\begin{aligned} M'_{ij} &= M_{ij} h_{A,i} h_{A,j} = \chi_{ij} \sum_k \sigma_i^{(k)} \sigma_j^{(k)} h_{A,i} h_{A,j} \\ &= \chi_{ij} \Big[\underbrace{\#(\sigma_i^{(k)} \sigma_j^{(k)} h_{A,i} h_{A,j} = 1)}_{\mu_{ij}} - \underbrace{\#(\sigma_i^{(k)} \sigma_j^{(k)} h_{A,i} h_{A,j} = -1)}_{\delta_{ij}} \Big] \end{aligned}$$

Euation (14) now becomes

$$E'(\mathbf{S}') = -\frac{1}{2} \sum_{i,j} \chi_{ij} \left(\mu_{ij} - \delta_{ij} \right) s'_i s'_j. \tag{S8}$$

Finding the minimum of $E(\mathbf{S})$ is equivalent to finding $\mathbf{S}'$ that minimizes $E'(\mathbf{S}')$ as defined in Eq. (S8). If $\mu_{ij} > \delta_{ij}, \forall i, j$, then $E'(\mathbf{S}')$ has two global minima: $s'_i = 1$ or $s'_i = -1$ corresponding to the parental haplotypes.

We next discuss how spin flipping and block switching can converge to these two minima starting from a random configuration

$$p(s'_{i,0} = 1) = p(s'_{i,0} = -1) = 1/2.$$

We first look at spin flips $s'_i \to -s'_i$. The associated energy changes are given by

$$\Delta E'_{i,0} = \sum_j \chi_{jk} \left(\mu_{ij} - \delta_{ij} \right) s'_{i,0} s'_{j,0} = s'_{i,0} h'_{i,0}. \quad \text{(spin flip)} \tag{S9}$$

By accepting spin flips that lower the energy function, we have

$$\begin{aligned} h'_{i,0} &> 0 \to s_{i,1} = -1; \\ h'_{i,0} &< 0 \to s_{i,1} = 1. \end{aligned}$$

The probability that two sites $s'_{i,1}$ and $s'_{j,1}$ are phased correctly relative to each other is given by

$$p(s'_{i,1} \cdot s'_{j,1} = 1) = p(h'_{i,0} \cdot h'_{j,0} > 0).$$

We have

$$\begin{aligned} h'_{i,0} \cdot h'_{j,0} &= \sum_{k,l} M'_{ik} M'_{jl} s'_{k,0} s'_{l,0} \\ &= \sum_k M'_{ik} M'_{jk} + \sum_{k \neq l} M'_{ik} M'_{jl} s'_{k,0} s'_{l,0}. \end{aligned} \tag{S10}$$

For a random configuration, we have

$$p(s'_{k,0} \cdot s'_{l,0} = 1) = p(s'_{k,0} \cdot s'_{l,0} = -1) = 1/2. \tag{S11}$$

When $M'_{ij} \geq 0$, we have

$$E(h'_{i,0} \cdot h'_{j,0}) = \sum_k M'_{ik} M'_{jk} + \sum_{k \neq l} M'_{ik} M'_{jl} E(s'_{k,0} \cdot s'_{l,0}) \approx \sum_k M'_{ik} M'_{jk} > 0, \tag{S12}$$

which implies

$$p(s'_{i,1} \cdot s'_{j,1} = 1) = p(h'_{i,0} \cdot h'_{j,0} > 0) > p(h'_{i,0} \cdot h'_{j,0} < 0) = p(s'_{i,1} \cdot s'_{j,1} = -1).$$

In other words, more sites are phased correctly relative to each other after one round of spin flipping due to the positive coupling in the first term in Eq. (S12). Equation (S12) further implies that as $p(s'_i \cdot s'_j = 1) \uparrow$ (*i.e.*, more sites are phased correctly), $E(s'_i \cdot s'_j) \uparrow$ and $E(h'_i \cdot h'_j) \uparrow$. Therefore, the haplotype solution will converge to either $\mathbf{H}_A$ or $\mathbf{H}_B$ locally through spin flips.

Although Eq. (S11) is true globally, it can be violated locally. An obvious example is at sites of long-range switching,

$$s'_i = 1,\ i \leq k;\ s'_i = -1,\ i > k \Rightarrow s'_i s'_j = -1,\ i \leq k < j.$$

These errors are most efficiently removed by block switching operations that lower the energy by

$$\Delta E_{k|k+1} = \sum_{i \leq k} \sum_{j > k} \chi_{ij} \left(\mu_{ij} - \delta_{ij}\right) s'_i s'_j \approx -\sum_{i \leq k} \sum_{j > k} \chi_{ij} \left(\mu_{ij} - \delta_{ij}\right) < 0. \tag{S13}$$

Therefore, as long as the signal of true haplotype linkage is much stronger than background noise ($\mu_{ij} \gg \delta_{ij}$, $M'_{ij} > 0$), iteration of spin flipping and block switching will converge **any initial state** to $\mathbf{S}' = 1$ or $\mathbf{S}' = -1$, and accordingly $\mathbf{S} \to H_A$ or H_B.

We can introduce more sophisticated minimization algorithms when the energy landscape of Eq. (14) contains many local minima. This happens when the linkage signal is sparse ($\mu_{ij} = 0$) or contains more errors $\delta_{ij} > \mu_{ij}$. Due to the presence of local minima, the final solution will depend on the initial haplotype configuration. We can use the same "steepest descent" strategy as described above but perform multiple simulations starting from different initial states to obtain a pool of haplotype solutions $\{\mathbf{S}^{(\alpha)}, \alpha = 1, 2, \cdots\}$, from which we can determine the optimal haplotype linkage between site i and j. For example, the haplotype linkage between site i and j in solution $\mathbf{S}^{(\alpha)}$ is given by $s_i^{(\alpha)} s_j^{(\alpha)}$ and its confidence can be estimated using

$$p_{i,j}^{(\alpha)} = \frac{1}{1 + e^{-\Delta E_{i,j}^{(\alpha)}}} \approx \begin{cases} 1 & \Delta E_{i,j}^{(\alpha)} \gg 0 \\ 1/2 & \Delta E_{i,j}^{(\alpha)} \sim 0 \\ 0 & \Delta E_{i,j}^{(\alpha)} \ll 0 \end{cases}$$

where

$$\Delta E_{i,j}^{(\alpha)} = \min\left(\Delta E_i^{(\alpha)}, \Delta E_j^{(\alpha)}, \Delta E_{i|j}^{(\alpha)}\right)$$

measures the overall probability of local (ΔE_i, ΔE_j) and switching ($\Delta E_{i|j}$) errors. We can infer the optimal haplotype linkage $\overline{s_i s_j}$ from the likelihood ratio that is similar to Eq. (3):

$$\frac{p(\overline{s_i s_j}=1)}{p(\overline{s_i s_j}=-1)} = \prod_{s_i^{(\alpha)} s_j^{(\alpha)}=1} \frac{p_{ij}^{(\alpha)}}{1-p_{ij}^{(\alpha)}} \prod_{s_i^{(\beta)} s_j^{(\beta)}=-1} \frac{1-p_{ij}^{(\beta)}}{p_{ij}^{(\beta)}}. \tag{S14}$$

To enable more efficient sampling of the configuration space in each simulation, one can also use the Metropolis algorithm for spin flipping and block switching moves.

Determination of the K-562 karyotype by haplotype-specific genomic analysis

The discussion in this section accompanies results presented in Additional file 5. All genomic coordinates are according to the GRCh38 human genome reference.

Cytogenetic karyotypes

Pages 2 and 3 show the cytogenetic karyotypes of K-562 cells (page 2) and selective marker chromosomes (page 3) analyzed using MFISH by Gribble *et al.* (2000)[42] and Naumann *et al.* (2001)[43].

Chromosomal copy number

Pages 5-7 show the normalized DNA copy number of each parental homolog (100kb bins) calculated using allelic depths from the linked-reads data in combination with parental haplotypes determined using the linked-reads data and the Hi-C data. Chromosomes 3,9,13,14 and X show complete loss-of-heterozygosity and the DNA copy number is calculated using the total sequencing depth. Segmental deletion/loss-of-heterozygosity affects Chr.2A (blue, q-terminus), Chr.10A (red, q-terminus), Chr.12A (blue, p-terminus), Chr.17B (blue, p-arm), Chr.20A (blue, p-arm) and Chr.22A (red, q-terminus). These regions are omitted in the allelic copy-number plots. We also assign large segmental copy-number alterations to derivative chromosomes that are labelled on top of the copy-number plots. The syntenic structures of these derivative chromosomes are discussed next. The only incompletely resolved segmental copy-number changes are gains of Chr.7q (blue homolog) and Chr.9q (outlined with boxes).

Digital karyotype

Pages 9 and 10 summarize the haplotype-resolved synteny of normal (page 9) and marker/derivative chromosomes (page 10). Among the structurally normal chromosomes, both copies of Chr.4B contain a deletion between 159,570,188 and 162,695,549 (annotated); Chr.16q contains a tandem duplication of sequence between 88,525,011 and 88,794,607 that is inferred to affect the B homolog by DNA copy number (not annotated). The only major discrepancy between the sequencing-derived karyotype and the cytogenetic karyotypes is seen in Chr.7. Both Gribble *et al.* and Naumann *et al.* reported a single normal Chr.7 plus three rearranged (marker) Chr.7. Naumann *et al.* inferred that one Chr.7 marker chromosome (M4) contained a paracentric inversion of p11-p22 and two Chr.7 marker chromosomes (M5 and M6) had rearrangements of both p- and q-arms resulting in a net gain on Chr.7q. Our DNA copy number analysis reveals a segmental gain of Chr.7q from ~117Mb to the q-terminus but we cannot detect any rearrangement either near the copy-number breakpoint or elsewhere on Chr.7 from either

linked-reads or Hi-C data. We think the normal Chr.7 in Ref.40 is the A homolog and the K-562 cells used to generate the linked-reads data (from which the DNA copy number is calculated) had gained an extra copy of Chr.7A. The marker chromosome (M4) with the paracentric inversion may have been lost. The two markers M5 and M6 are both derived from the B homolog, but the fusion breakpoints are likely located in heterochromatic regions (centromere/telomere) that cannot be resolved by shotgun sequencing. Among the marker chromosomes, the composition of t(22A;9-13-22hsr) is inferred using both sequencing and cytogenetic data (Page 3) and should be taken as a model.

Marker chromosomes with completely resolved translocations

Pages 11-16 summarize the analysis of sequencing data that completely determines the syntenic blocks and junctions in five marker chromosomes: t(5A;6A), t(12A;21A), t(3A;10A), t(3A;10A;17A), and t(6A;16A;6B). All of these marker chromosomes were also identified by Gribble *et al.* and Naumann *et al.* For each chromosome, we first identify the syntenic blocks from haplotype-specific DNA copy number and Hi-C contacts; we then refine the junctions between blocks first using the barcode map generated from linked-reads data and then by sequencing reads with split alignments. Each phased Hi-C contact map contains 9 panels: the bottom left panel shows the density map of unphased Hi-C contacts; the upper left and bottom right panels show the density maps of Hi-C contacts where one end of the contact is phased (A-U, B-U, U-A, and U-B); the upper right panels are scatter plots of all Hi-C contacts with both ends phased (A-A, A-B, B-A, and B-B). The density map of molecular barcodes shows the number of unique molecular barcodes with sequencing fragments mapping to loci on both axes. A fusion between distal loci results in a sharp increase in the density of off-diagonal contacts in both Hi-C and molecular barcode maps; the breakpoints are annotated using red lines meeting at the apex with the highest density of contacts/barcodes. The barcode density map is generated with 10kb bins, which on average contains about 100 unique molecular barcodes per chromosome. The density of molecular coverage allows the inference of the copy number of the translocated chromosome. For example, t(5A;6A), t(12A;21A), and t(6A;16A;6B) all have one copy per genome; the junction t(3A;10A) has two copies per genome, although one copy has an additional translocation to Chr.17 to make t(3A;10A;17A).

There are a few notable examples. In t(3A;10A;17A), we infer the fusion between Chr.10A and Chr.17A occurs at the centromere; we have omitted the molecular barcode map as the junction is not represented in the human genome reference and therefore cannot be resolved by shotgun sequencing. t(6A;16A;6B) is special as it involves both Chr.6 homologs. The resolution of the structure of this chromosome is possible because the parental Chr.6 haplotype can be determined from Hi-C contacts within the normal Chr.6B, even though there is no normal Chr.6A. The proximity of breakpoints on Chr.6A at 37,856,135 in t(5A;6A) and at 38,139,520 in in t(6A;16A;6B) suggests that these two breakpoints/translocations resulted from a single DNA break on Chr.6A near 38Mb.

Marker chromosomes with incompletely resolved translocations

Pages 17-21 summarize the analysis of derivative/marker chromosomes with one or more junctions not completely resolved. Nearly all of the incompletely resolved junctions contain DNA sequence with ambiguous origin (most likely derived from heterochromatic regions).

We infer t(2A;22A) (Page 18) to contain two inverted duplication (“foldback”) events on Chr.2 followed by a translocation to Chr.22. This chromosome corresponds to M1 in Ref. [43]. Naumann *et al.* reported that the added Chr.22 segment also contains Chr.9 material that forms the *BCR-ABL* fusion. Consistent with this result, we observe an increase in Hi-C contact density between Chr.2 and Chr.9 (data not shown) but cannot determine the telomeric end of this chromosome.

We infer t(1B;21B) (Page 19) to be generated by a translocation between Chr.1B at 162,379,862 and the acrocentric arm of Chr.21B, but cannot determine the breakpoint location on Chr.21.

We determine the composition of t(6A;1A;20A) (Pages 20 and 21) from the Hi-C maps and further detect a small piece from Chr.18 inserted between Chr.6A and Chr.1A (Page 22). For the Chr.1:Chr.18 junction, both breakpoints are resolved by sequencing reads. For the Chr.18:Chr.6 junction, the breakpoint on Chr.6 is almost certainly located at 135,580,256 and the breakpoint on Chr.18 is likely located at 27,330,533, but we cannot assemble the complete junction sequence as these breakpoints are joined by DNA sequence with ambiguous origin (likely derived from heterochromatic regions). We are unable to detect an increase in the molecular barcode density between Chr.6 and Chr.18 breakpoints in the linked reads data, indicating that the inserted sequence may be longer than 100kb.

Besides t(6A;1A;20A), we infer both Chr.18 homologs to be involved in complex rearrangements giving rise to t(1A;18A) and t(3A;18B) (Pages 22-26). Page 22 shows the density map of unphased Hi-C contacts between Chr.18 and Chr.1 and between Chr.18 and Chr.3. The breakpoints in t(1A;18A) are resolved to be Chr.1:54,726,344 and Chr.18:25,907,722 (Page 23). The breakpoint on Chr.3 in t(3;18A) is resolved to be Chr.3:138,669,875 (Page 24) but it is joined with DNA sequence with ambiguous origin. The partner breakpoint on Chr.18 cannot be determined with certainty. Interestingly, both the Chr.1A junction and the Chr.3A junction contain inverted duplications (opposite facing arrows) near the breakpoint.

To completely resolve the alterations to both Chr.18 homologs, we jointly analyze rearrangement breakpoints and haplotype-specific DNA copy number of Chr.18 (Page 26). The Chr.18A homolog contains a foldback rearrangement at 42Mb and 3 other breakpoints (24,413,290, 24,822,840, and 41,843,305) whose fusion partners cannot be identified. An inspection of the Hi-C contact map between Chr.1 and Chr.18 reveals that the foldback rearrangement at 42Mb is part of t(1A;18A). Given that there are two p-arms of Chr.18A but only one normal q-terminus of Chr.18A, we infer that there is one normal Chr.18A and the other Chr.18A p-arm is connected to the rearranged segments on Chr.18q and then joins Chr.1A to form a stable chromosome with two telomeres.

We identify 18 breakpoints on Chr.18B. A close inspection of the Hi-C contact map between Chr.3 and Chr.18 (Page 24) reveals that the rearranged Chr.18B is connected to Chr.3. The fusion between breakpoints at 39,768,382 and 40,287,508 results in a tandem duplication and appears to be unrelated to the others. Among the remaining 16 breakpoints on Chr.18B, 12 are paired and result in little or no DNA copy number change; the breakpoint at 26.97Mb is a foldback rearrangement; three breakpoints (at 10,860,074, 21,920,738, and 27,509,642) are accompanied with DNA copy-number changes. 14 breakpoints are connected by rearrangements (a-e on Page 25) detected from linked reads and further validated on the Hi-C contact map. The junction between

Chr.18:8,110,157 and 26,776,296 is not visible on the Hi-C map. It is evident from the barcode map that this junction is linked to another breakpoint near 26,776,296, which is identified to be located at 26,770,719 but with an unidentifiable fusion partner. From the Hi-C contact map we infer the breakpoint at 8,110,157 eventually joins the breakpoint at 27,509,642 that also has an unidentified partner. Since the only other breakpoint on the B homolog (blue) with an unidentified partner is at 23,730,830, we infer that Chr.18:23,730,830 is connected to Chr.3A at 138,669,875 with additional sequence insertion.

Chromosome 9 has by far the most alterations and was inferred to participate in four marker chromosomes (M7, M8, M15 and M16 in Ref. [43]) in addition to the *BCR-ABL* amplicon present in t(2A;22A) and t(22;hsr). We are able to partially resolve three marker chromosomes involving Chr.9 (Pages 27-31).

We first infer that three Chr.9 fragments are partitioned to separate marker chromosomes from the Hi-C map (Page 27). The p-terminal segment (0-20.75Mb) joins Chr.17q (B homolog) at the centromere, as determined from the Hi-C contact map between Chr.9 and Chr.17 (Page 28). The t(9;17B) chromosome is present at two copies and match M16 in Ref. [43].

The two segments Chr.9:26.58-28.55Mb and Chr.9:31.62-38.43Mb show few contacts with the rest of Chr.9 (Page 27) but form extensive contact throughout Chr.13q (Page 29), suggesting a derivative chromosome t(9;13). The only copy-number breakpoint with an unidentified fusion partner is Chr.9:32,239,218, which most likely joins Chr.13 (Page 30). The t(9;13) matches M15 reported in Ref. [43].

The remaining Hi-C contacts are consistent with a marker Chr.9 with deletion from the p-terminus to ~37Mb. We additionally identify a foldback rearrangement at 37.08Mb that marks the distal end of this chromosome. This chromosome matches M8 reported in Ref. [43] that is annotated as del(9)(:p12-qter). We also detect a gained segment on Chr.9q from 91,321,259 to the q-terminus but cannot map the fusion partner of the breakpoint. Naumann *et al.* reported that this segment is fused to the q-terminus of Chr.9 and forms M7.

Finally, we attempt to infer the structure of the amplicon in the t(9;13;22) chromosome that contains multiple copies of the *BCR-ABL* fusion gene. We start with the amplified segment on Chr.9. This segment is flanked by three breakpoints: 130,731,760 at the 5'-end (fusion 1), 131,199,197 (fusion 2) and 131,280,137 (fusion 3) on the 3'-end.

The breakpoint at Chr.9:131,280,137 joins Chr.13:108,009,063 (fusion 3) and is connected to multiple amplified segments on Chr.13q (Pages 31 and 32). The amplicons on Chr.13q have roughly the same DNA copy number ~12 (Page 33); the rearrangements further suggest that the individual segments are joined together and then amplified as a single unit. Because there is only one junction connecting the Chr.13 fragments to Chr.9, we infer that the Chr.13 fragments form a linear palindromic structure with a foldback junction at Chr.13:91,821,757><91,823,504 and both ends of the palindrome join Chr.9 through fusion 3.

The breakpoint at Chr.9:131,199,197 joins Chr.22:16,819,349 (fusion 2) and its copy number is estimated to be ~8 (Page 33); the breakpoint at Chr.9:130,731,760 joins Chr.22:23,290,555 and creates the *BCR-ABL* fusion gene (fusion 1) with an estimated DNA copy number ~20. In contrast to Chr.9 and Chr.13, Chr.22 shows multiple copy-number states that are consistent with breakage-fusion-bridge cycles interspersed with

chromothripsis. We further identify two rearrangement junctions on Chr.22 with similar DNA copy number as fusion 2. One event is a fusion between Chr.22:18,965,610 and Chr.22:22,592,903, the other is a foldback rearrangement at chr22:16,243,545-16,245,357. The other copy-number breakpoints on Chr.22 all have less DNA copy number and therefore most likely occurred after the initial amplification. Putting all information together, we infer that the Chr.9:Chr.22 amplicon is generated from four segments: Chr.22:16.24-16.81Mb, Chr.9:130.73-131.20Mb, Chr.22:22.59-23.59Mb, and Chr.22:18.96-23.29Mb; these segments are joined in tandem and then form a palindrome with a foldback junction at Chr22:16,243,545><16,245,357. The ends of the palindrome join Chr.9 through fusion 1.

We further infer that the 9:13 amplicon and the 9:22 amplicon are joined together in a single amplicon that is amplified in an inverted tandem array structure. The tandem array configuration is consistent with MFISH analysis in Ref. [42] indicating that the 9:13:22 amplicon creates a homologous staining region (HSR) in two marker chromosomes each containing 4-5 copies of the Chr.13 probe (Page 32). We speculate that the slightly higher DNA copy number of the 9:13 amplicon ($\sim$12) than the 9:22 amplicon ($\sim$8) is due to extra copies of the 9:13 amplicon in the t(2A;22A) derivative chromosome. The basic unit of the tandem array is a palindrome formed by inverted duplication of a single 9:13:22 amplicon; the palindrome then undergoes multiple rounds of duplications in tandem. Interestingly, the tandem array only consists of foldback rearrangements but all DNA sequence is amplified uniformly. This contrasts with amplification by the breakage-fusion-bridge cycles that are also generated by foldback rearrangements but usually result in varying DNA copy number. We think the foldback rearrangements in the 9:13:22 HSR were first generated by breakage-fusion-bridge cycles, but the creation of almost perfect palindromic sequences (Chr.13:91,821,757><91,823,504 and Chr22:16,243,545><16,245,357) leads to more frequent amplifications by homology-dependent DNA recombination or replication errors. We speculate that the formation of palindromic sequence by foldback rearrangements between breakpoints in close proximity may be a hallmark features of HSRs.
