# Supplementary material for: Determination of complete chromosomal haplotypes by bulk DNA sequencing
Source: Genome Biol. 2021 May 6;22:139. doi: 10.1186/s13059-021-02330-1 (PMC8101039; doi:10.1186/s13059-021-02330-1)

K-562: cytogenetic karyotypes

Gribble et al. Cancer Genetics and Cytogenetics, 2000.

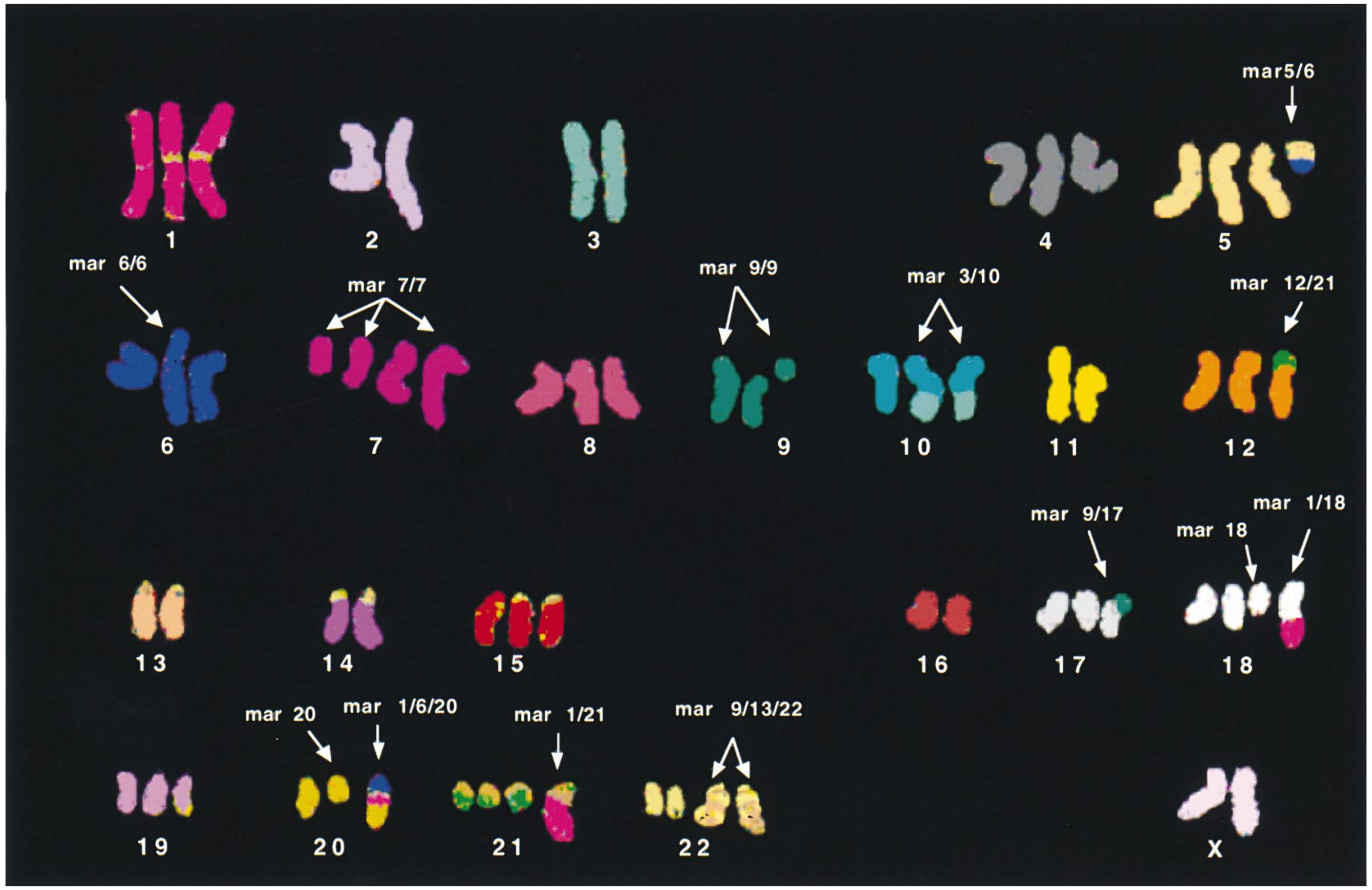

Naumann et al. Leukemia Research, 2001.

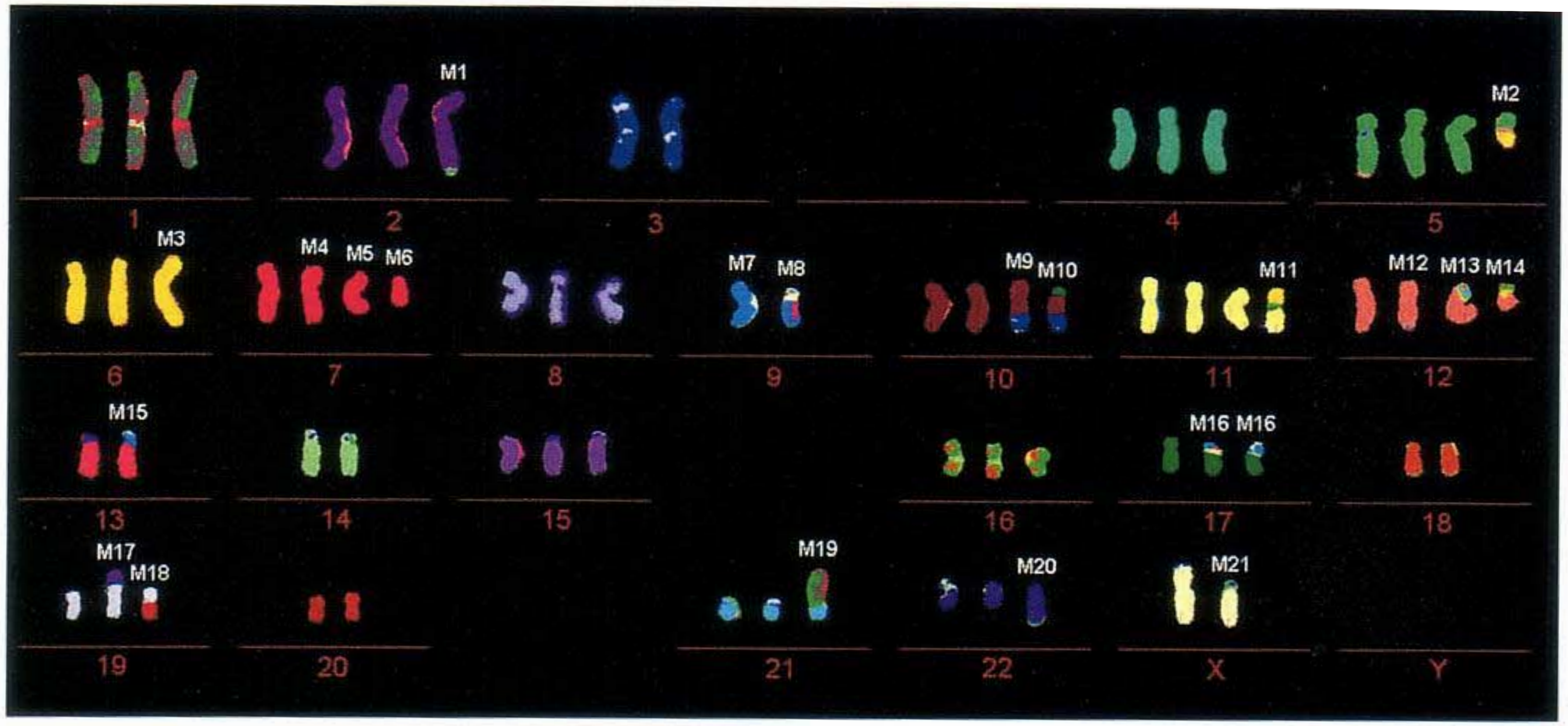

Selected marker chromosomes

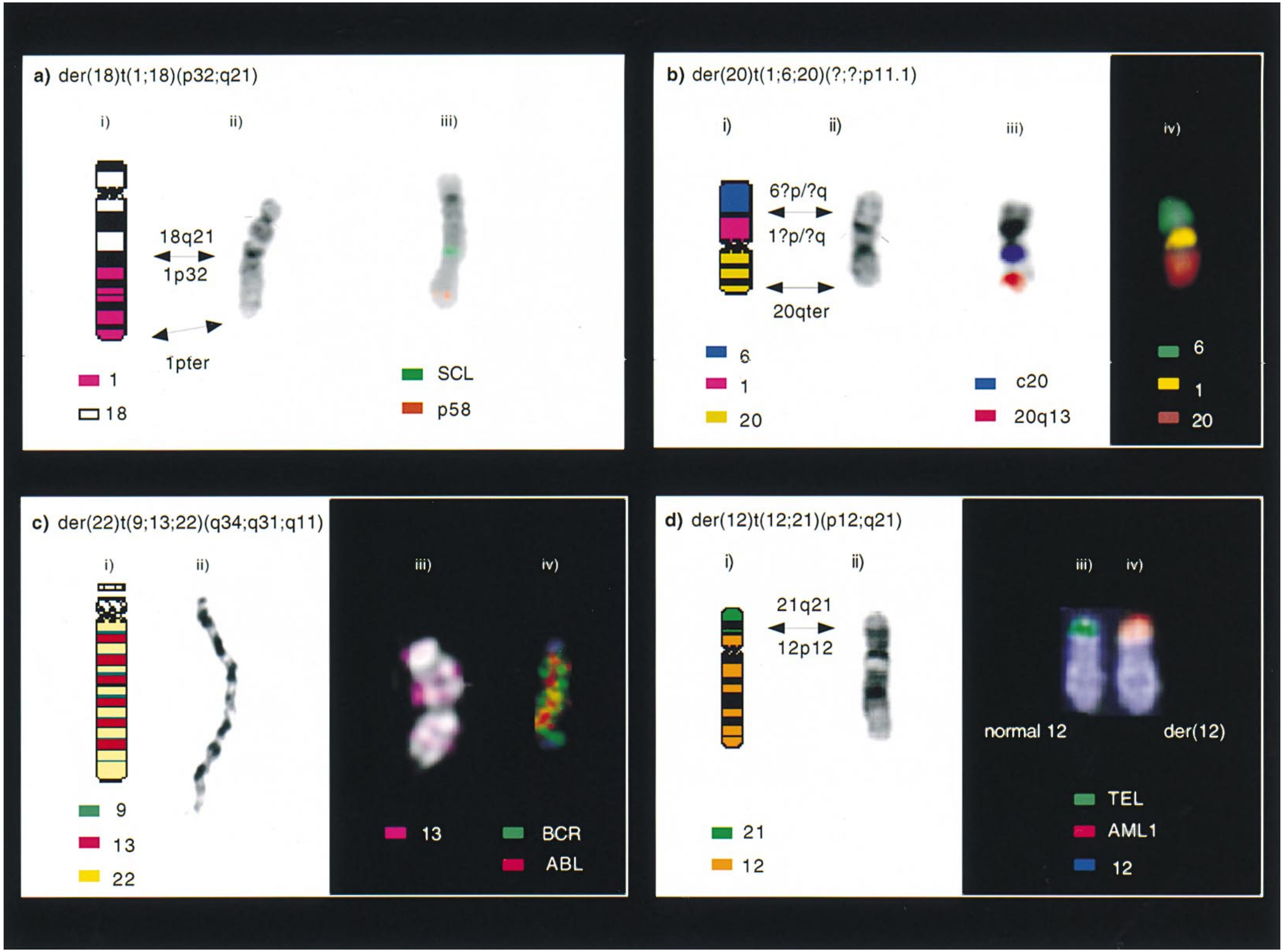

Gribble et al. Cancer Genetics and Cytogenetics, 2000.

Chromosomal copy number

Chromosomal copy number

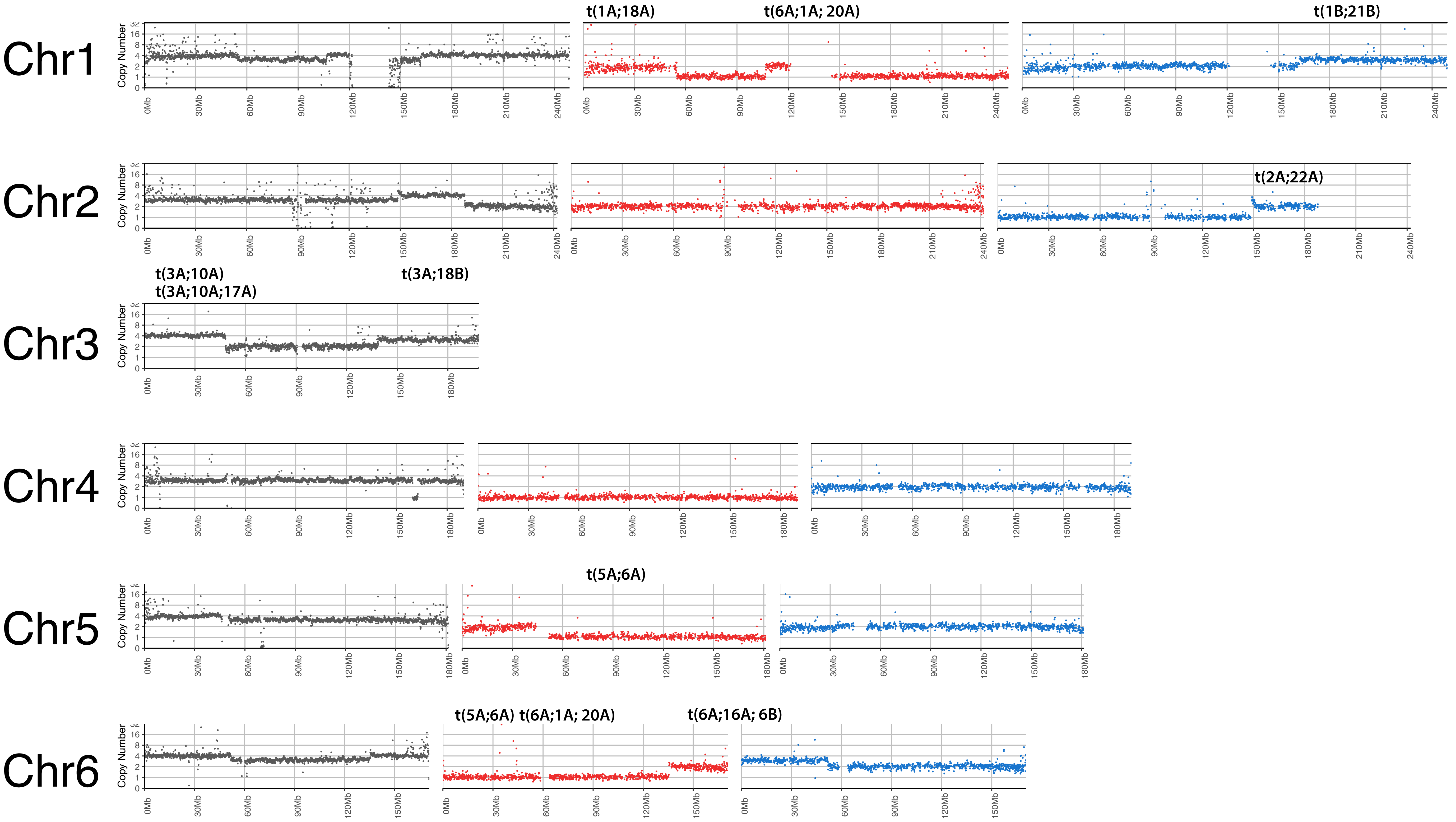

Chromosomal copy number

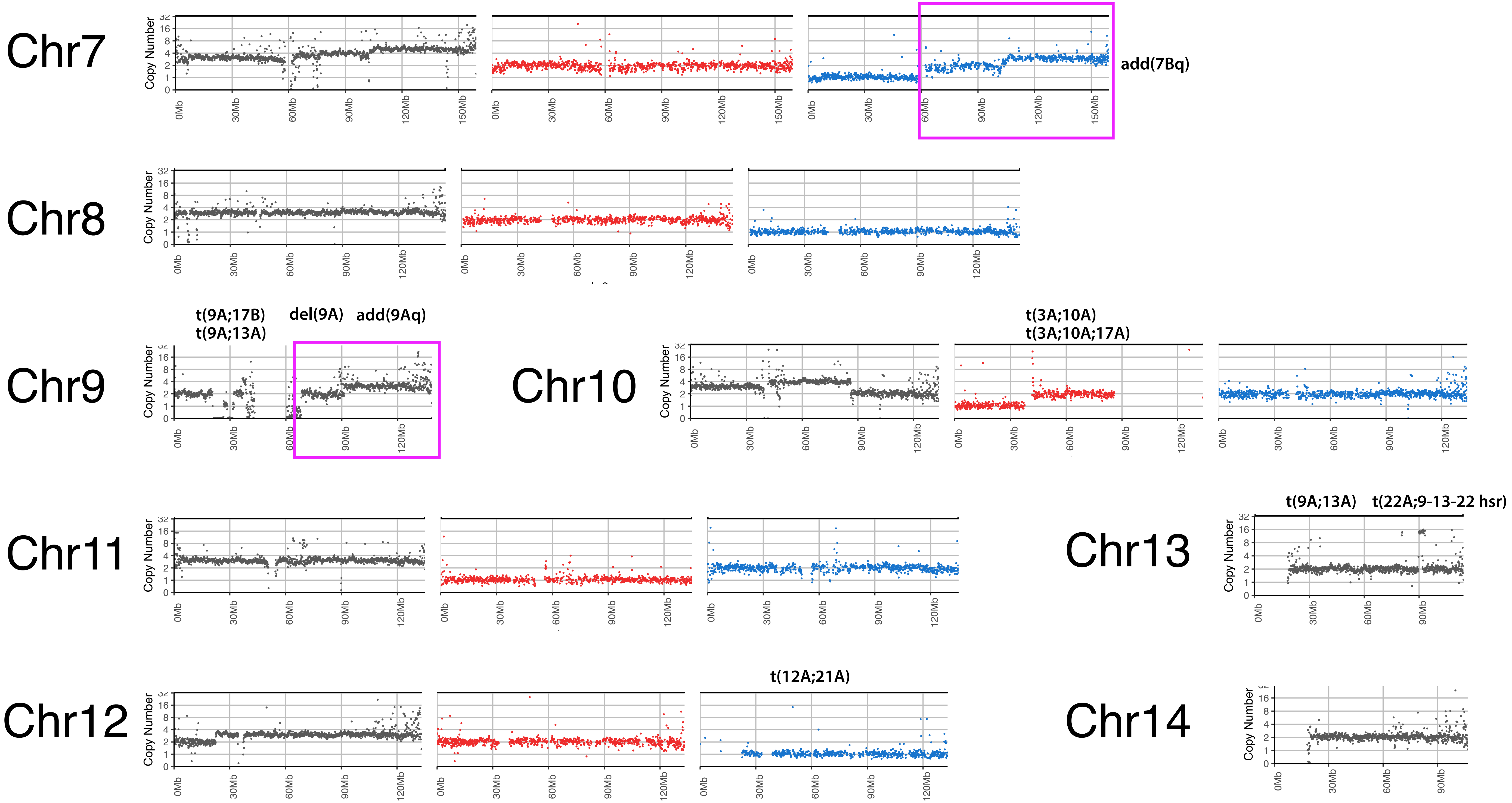

Chromosomal copy number

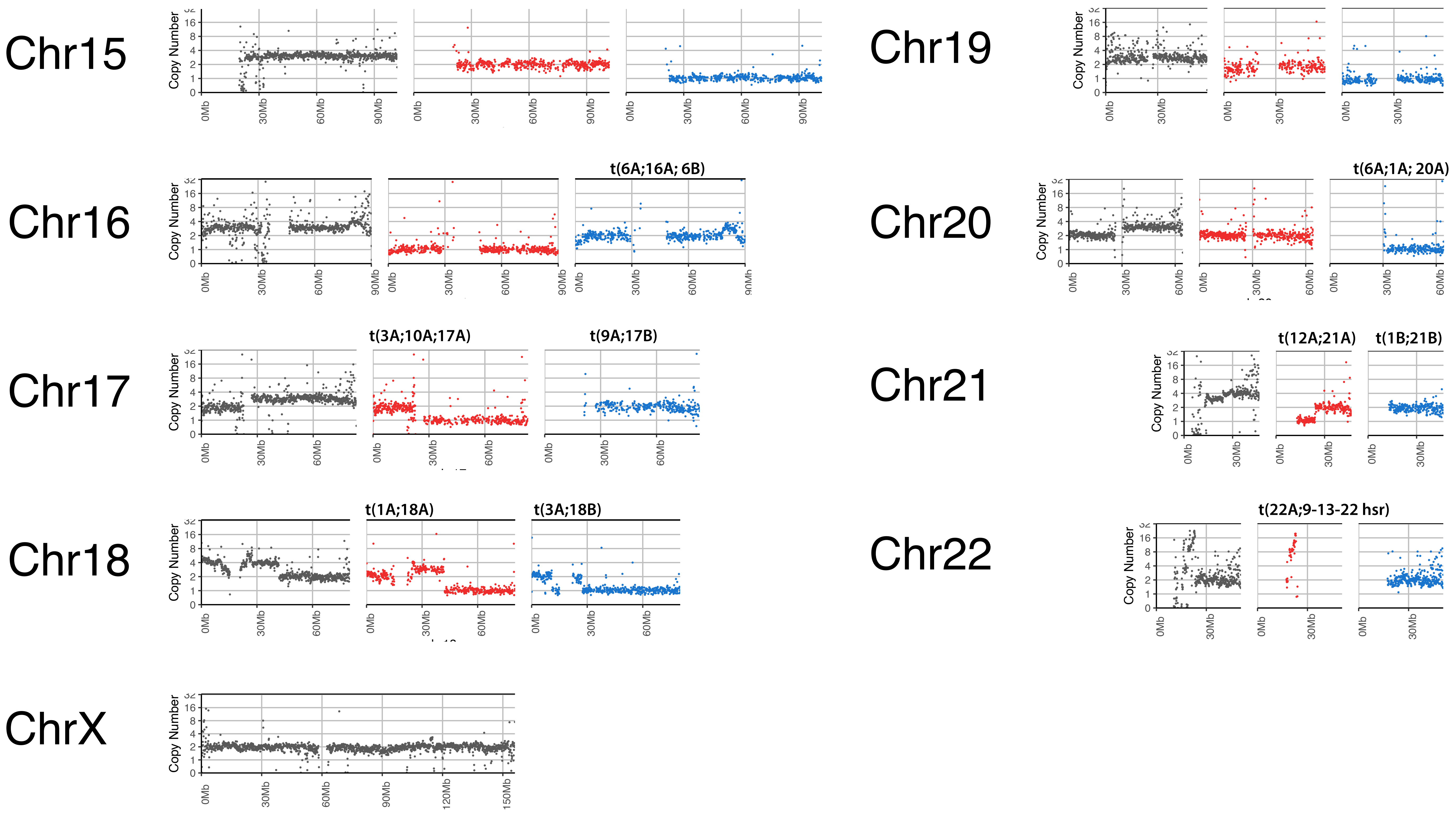

Digital karyotype

Structurally normal chromosomes

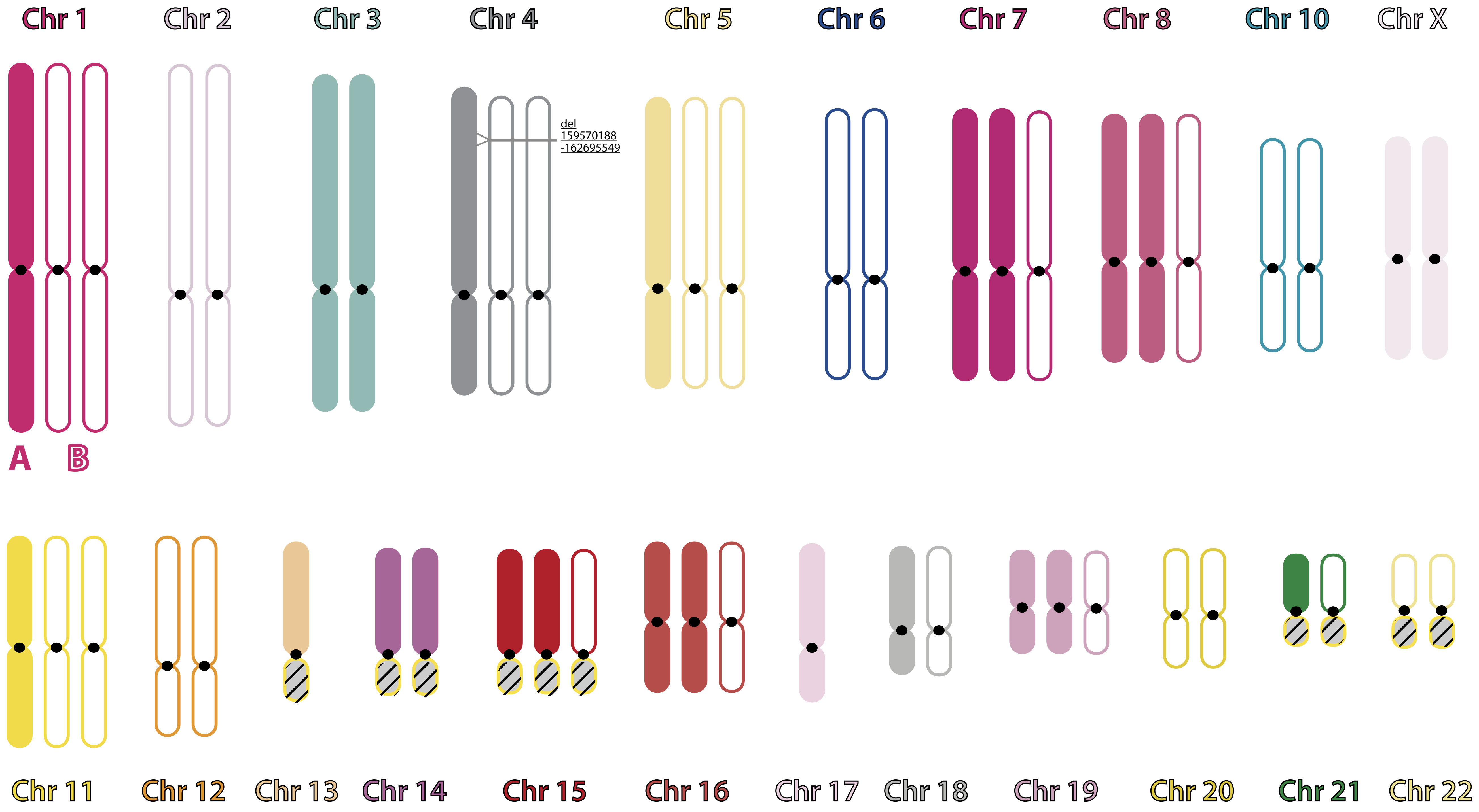

Marker chromosomes

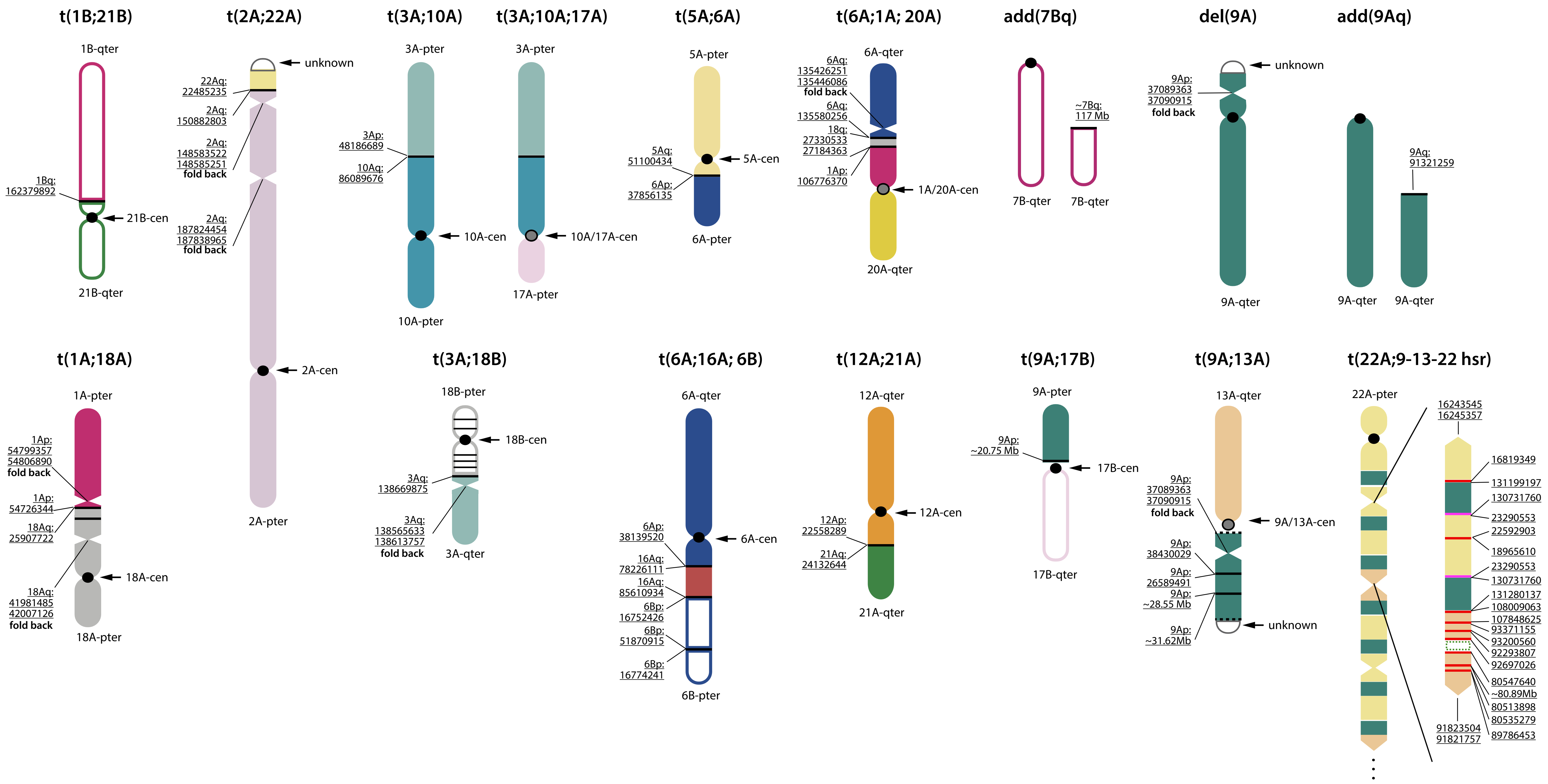

Marker chromosomes with completely resolved translocations

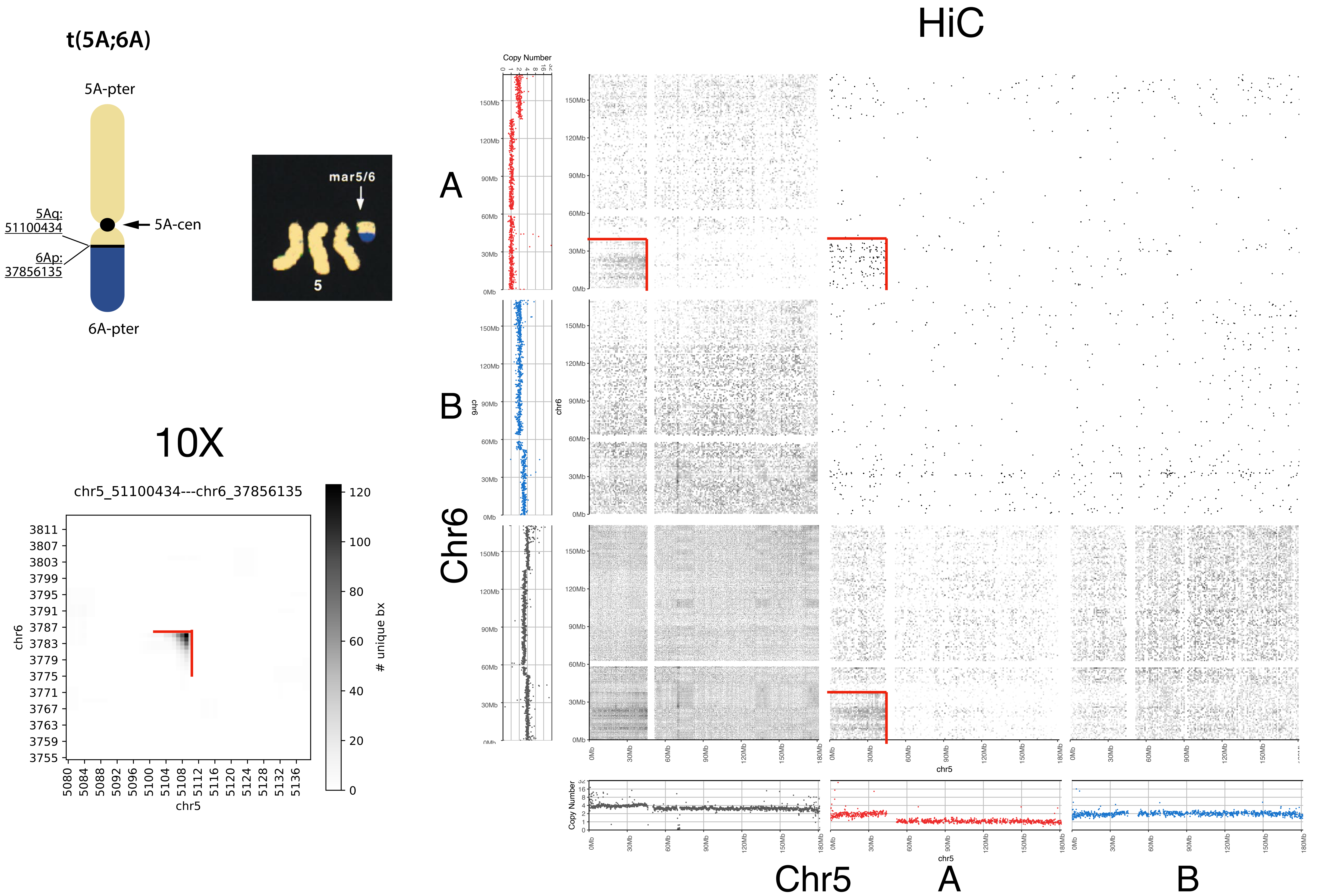

t(5A;6A)
5A-pter
5Aq: 51100434
5A-cen
6Ap: 37856135
6A-pter
mar5/6
5
10X
chr5_51100434---chr6_37856135
unique bx
chr6
chr5
HiC
Copy Number
A
B
Chr6
Chr5
A
B

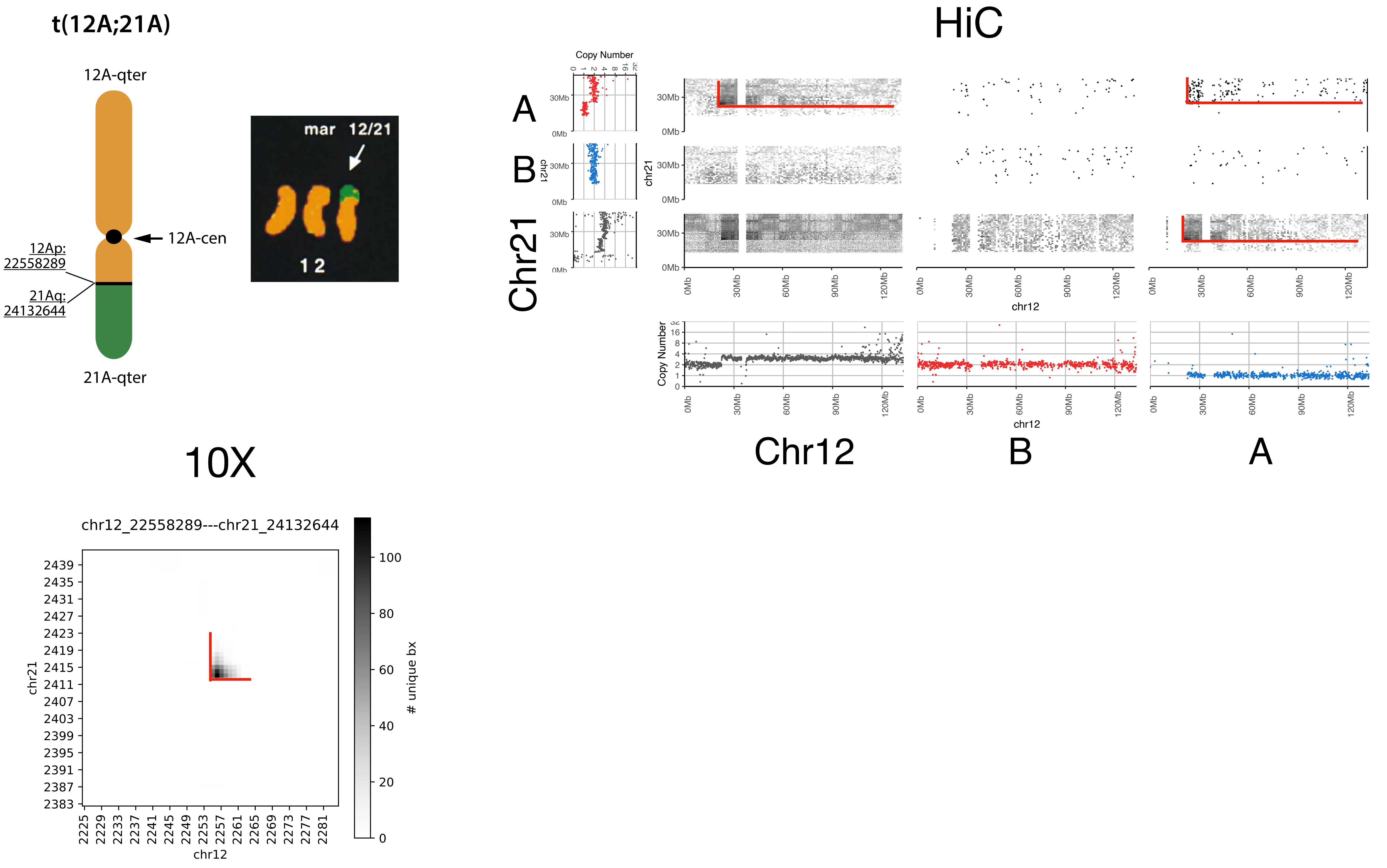

t(12A;21A)
12A-qter
12A-cen
12Ap:
22558289
21Aq:
24132644
21A-qter
mar 12/21
1 2
HiC
Copy Number
A
B
Chr21
chr21
chr12
Copy Number
Chr12
B
A
10X
chr12_22558289---chr21_24132644
chr21
chr12
unique bx

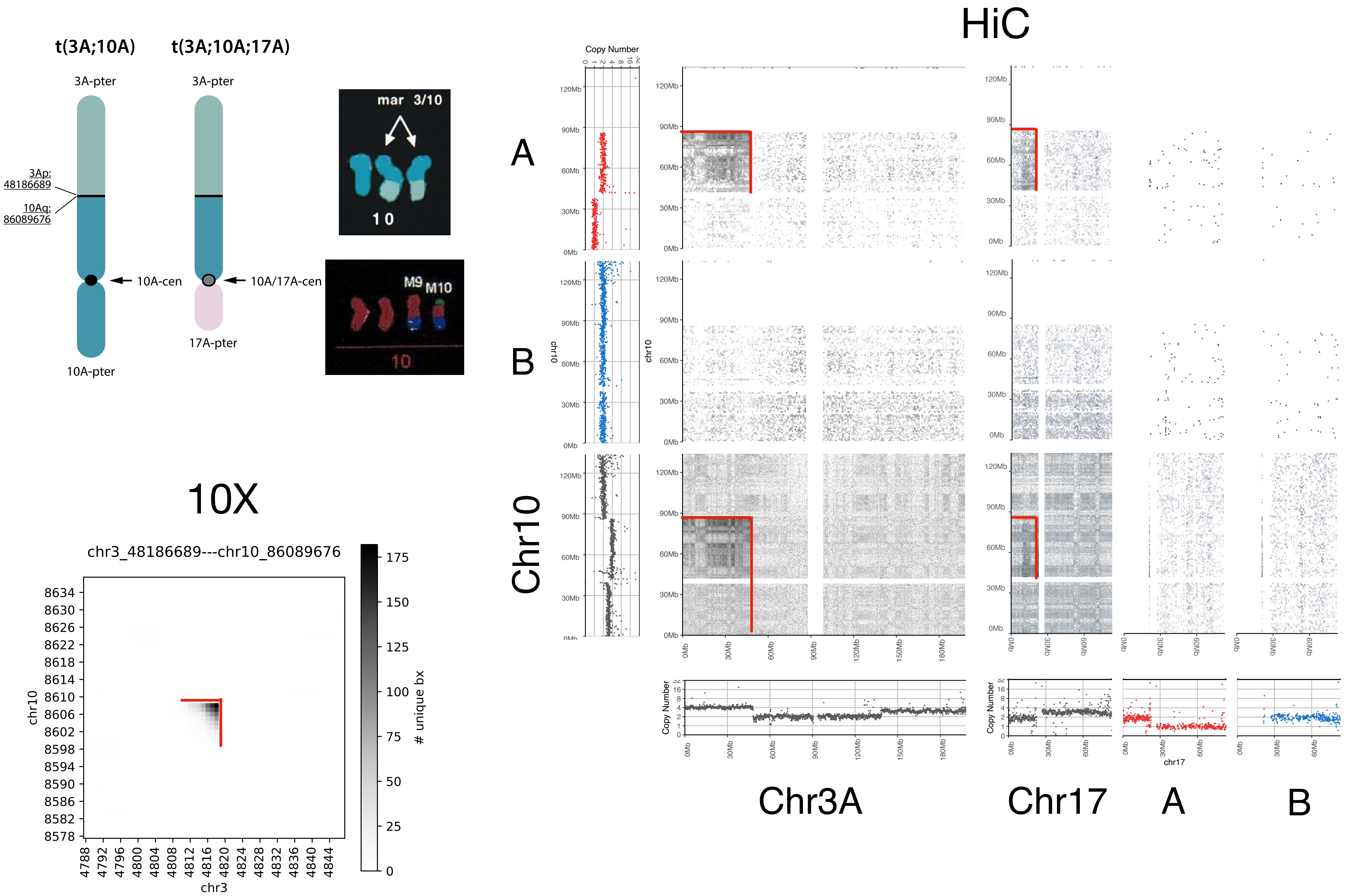
t(3A;10A)
t(3A;10A;17A)
3A-pter
3Ap:
48186689
10Aq:
86089676
10A-cen
10A/17A-cen
10A-pter
17A-pter
mar 3/10
10
M9
M10
HiC
Copy Number
A
B
Chr10
chr10
Chr3A
Chr17
chr17
10X
chr3_48186689---chr10_86089676
chr3
unique bx

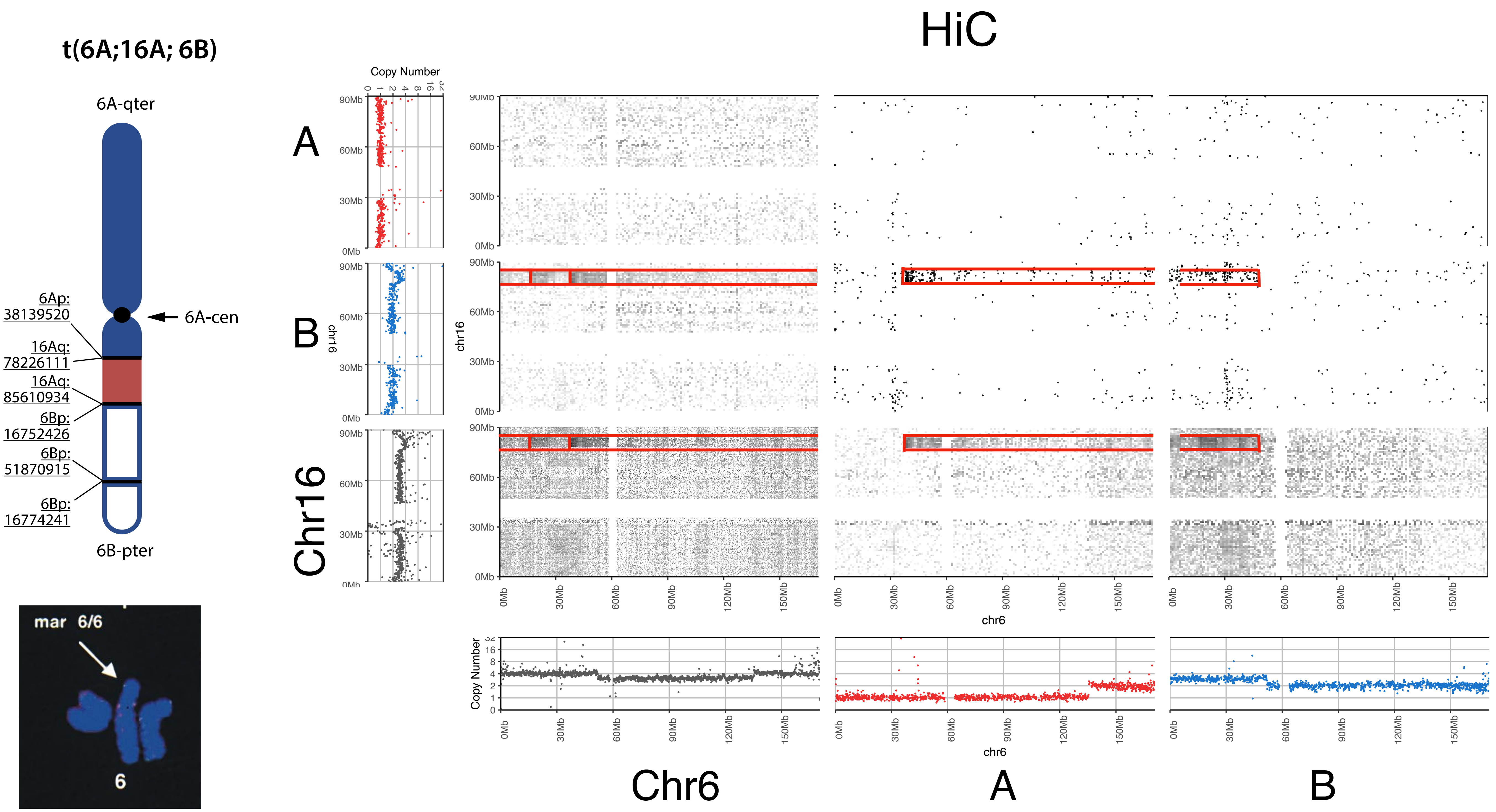

t(6A;16A; 6B)
6A-qter
6Ap: 38139520
6A-cen
16Aq: 78226111
16Aq: 85610934
6Bp: 16752426
6Bp: 51870915
6Bp: 16774241
6B-pter
mar 6/6
6
HiC
Copy Number
A
B
Chr16
chr16
chr6
Chr6
A
B

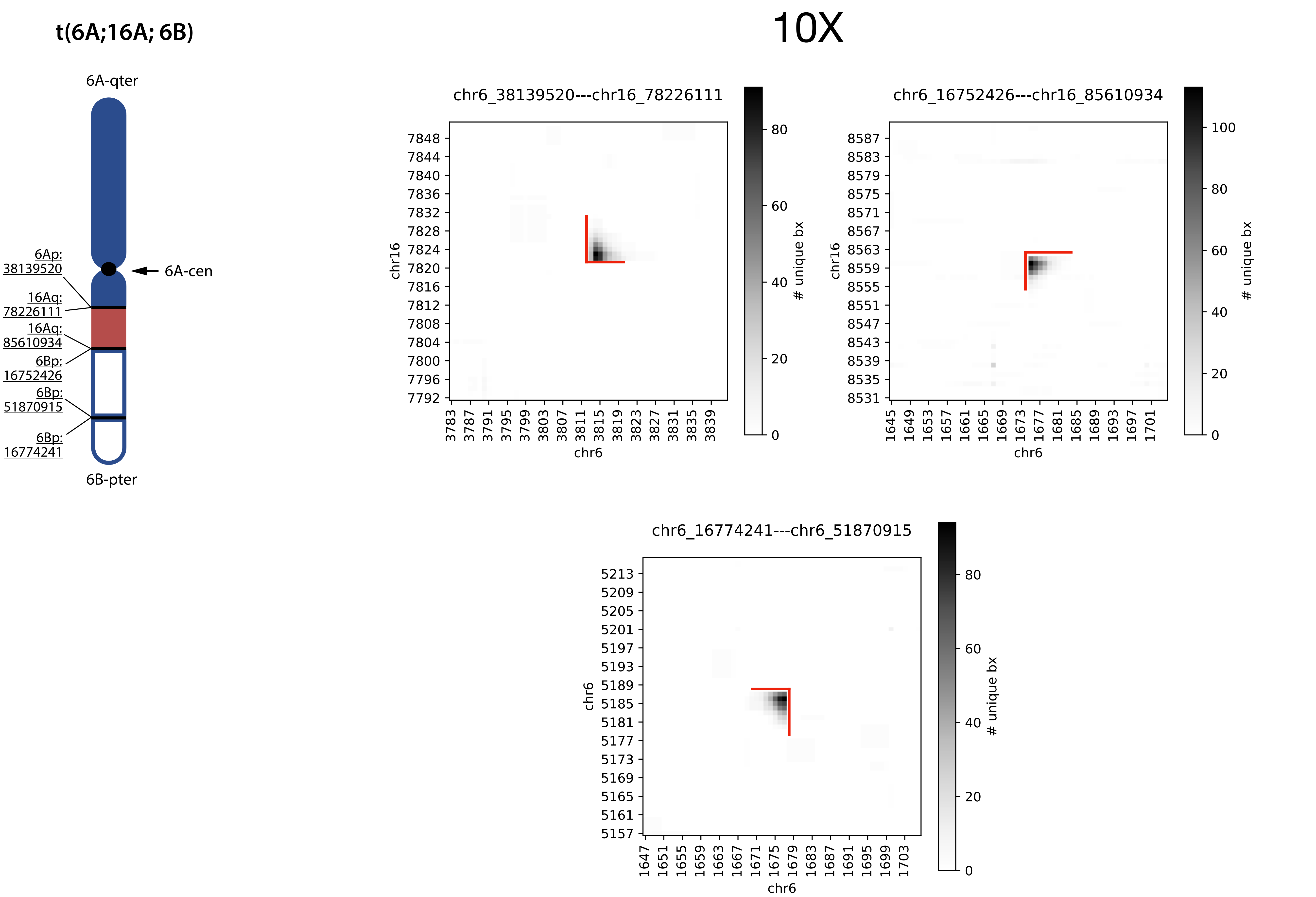

t(6A;16A; 6B)
10X
6A-qter
6Ap: 38139520
16Aq: 78226111
16Aq: 85610934
6Bp: 16752426
6Bp: 51870915
6Bp: 16774241
6A-cen
6B-pter
chr6_38139520---chr16_78226111
chr6_16752426---chr16_85610934
chr6_16774241---chr6_51870915
chr16
chr6
unique bx

Marker chromosomes with incompletely resolved translocations

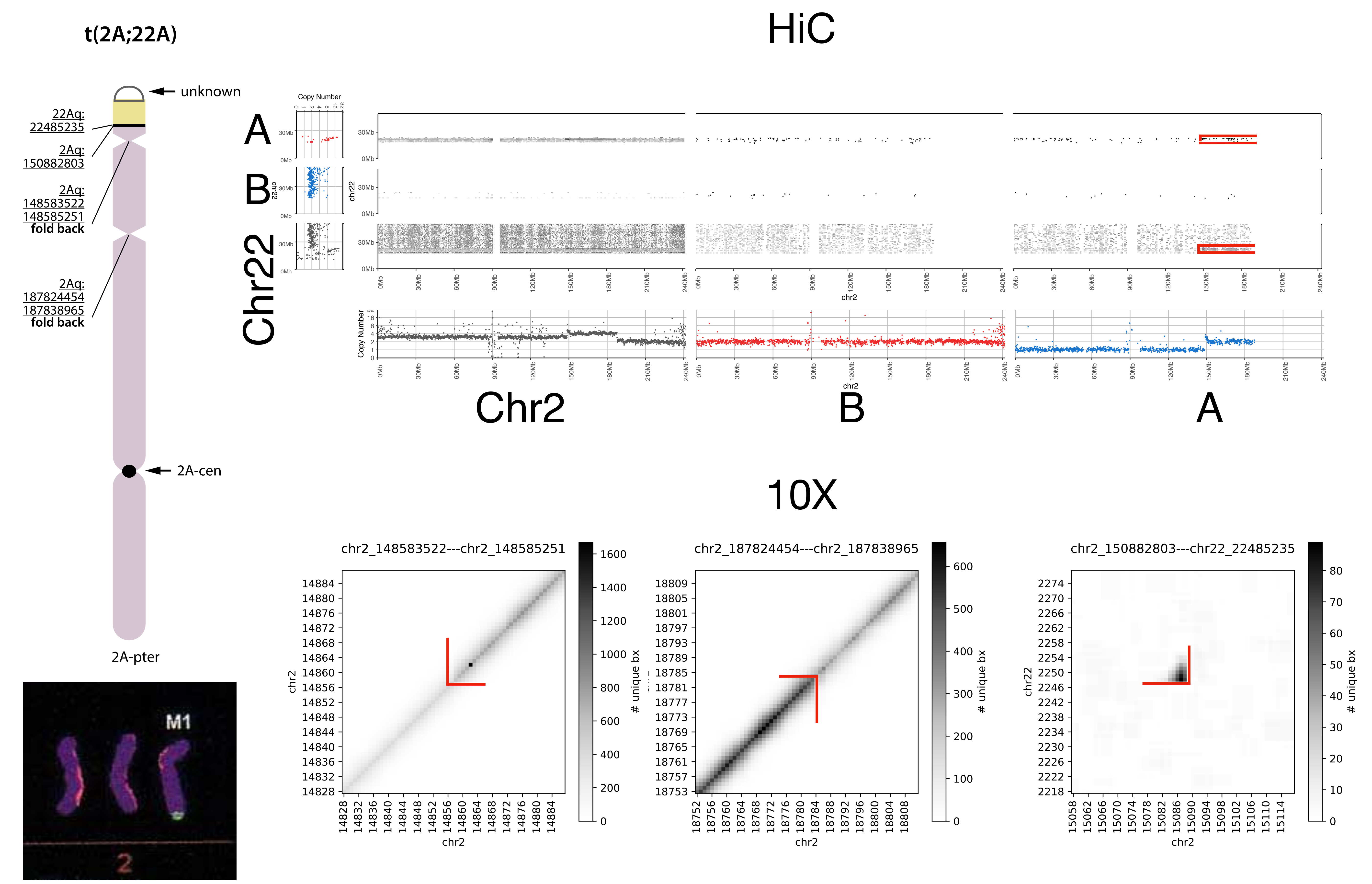

t(2A;22A)
unknown
22Aq: 22485235
2Aq: 150882803
2Aq: 148583522 148585251 fold back
2Aq: 187824454 187838965 fold back
2A-cen
2A-pter
M1
2
HiC
A
B
Chr22
Chr2
B
A
10X
chr2_148583522---chr2_148585251
chr2_187824454---chr2_187838965
chr2_150882803---chr22_22485235
unique bx

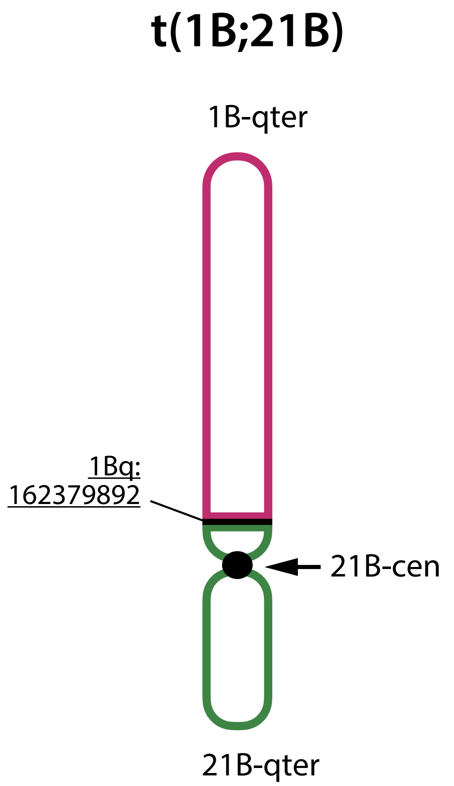
t(1B;21B)
1B-qter
1Bq:
162379892
21B-cen
21B-qter

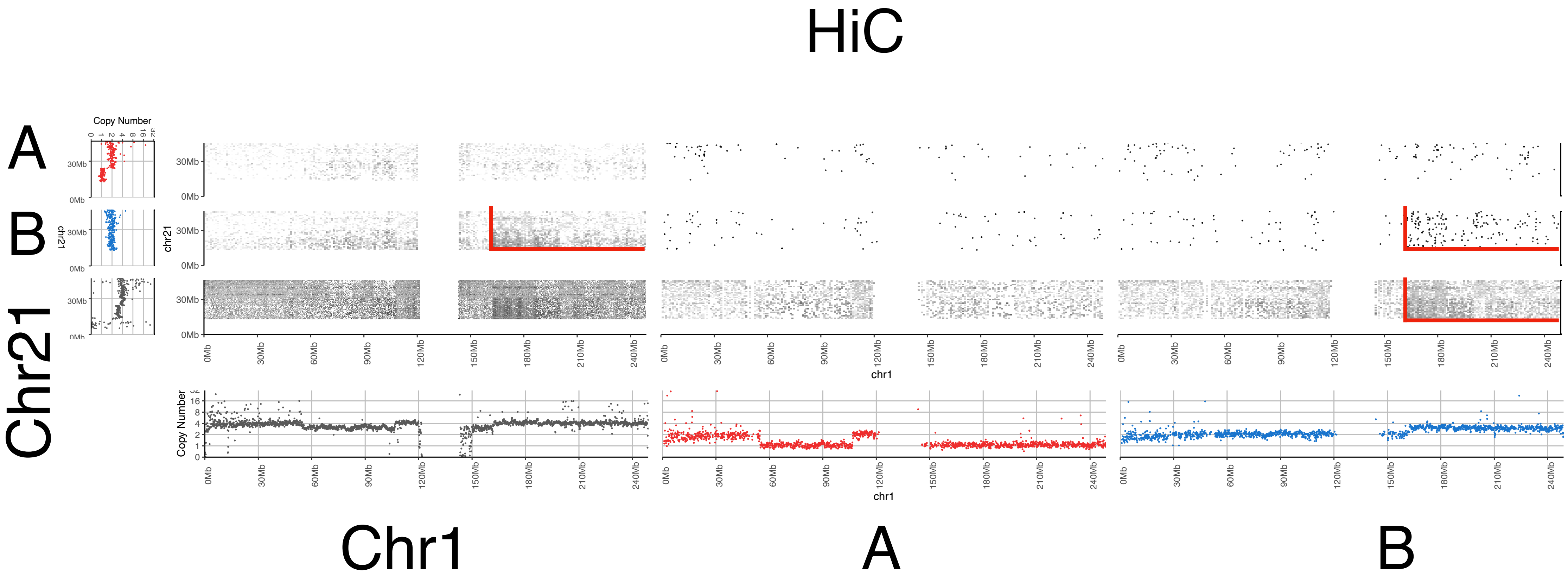
HiC
A
B
Chr21
Copy Number
chr21
Copy Number
chr1
Chr1
A
B

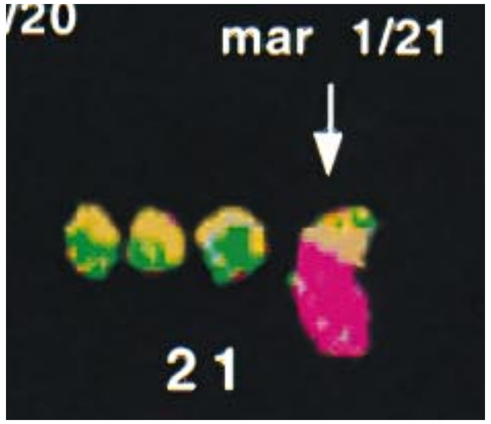
/20
mar 1/21
21

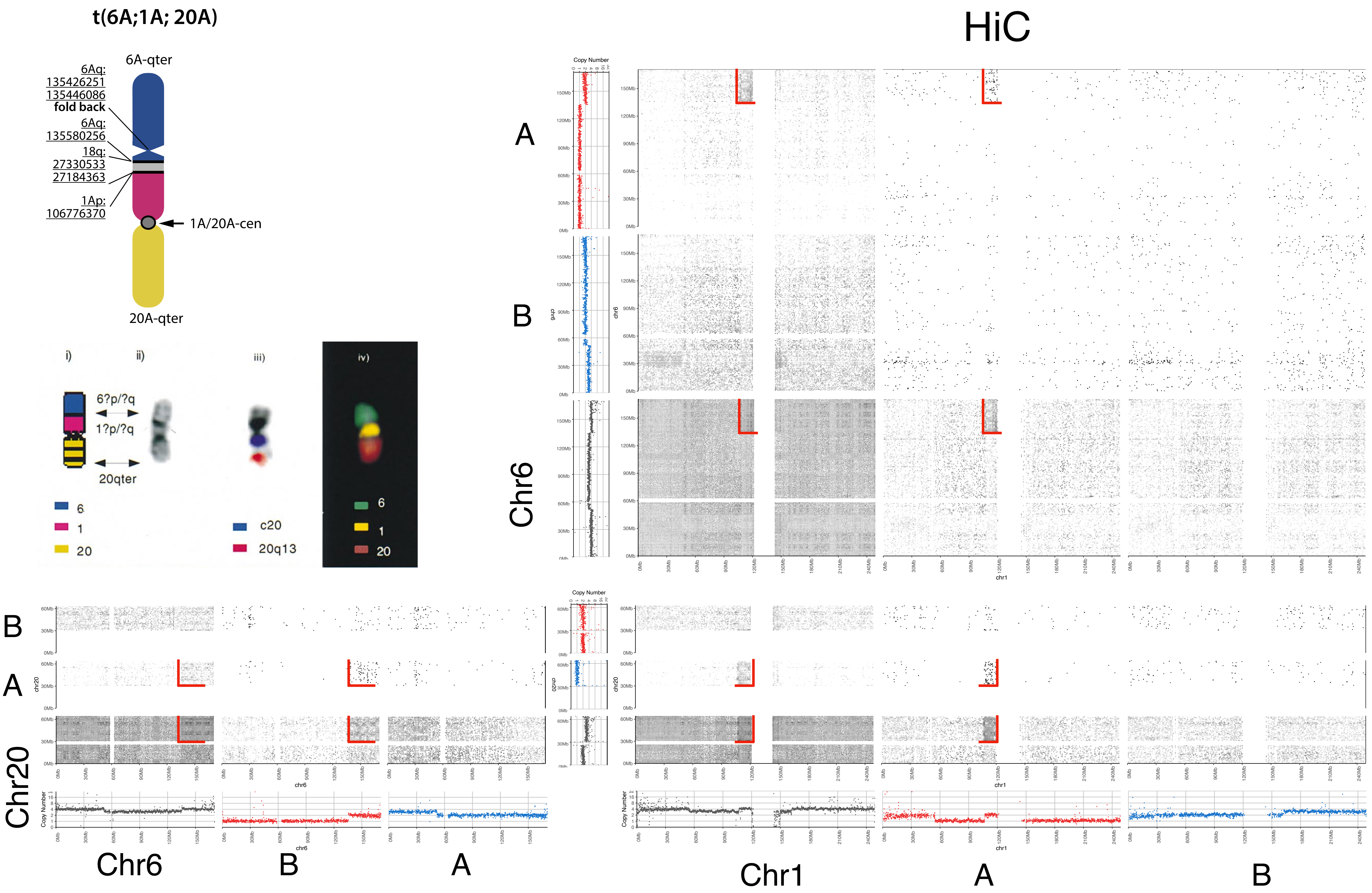

t(6A;1A; 20A)
6A-qter
6Aq:
135426251
135446086
fold back
6Aq:
135580256
18q:
27330533
27184363
1Ap:
106776370
1A/20A-cen
20A-qter
i)
ii)
iii)
iv)
6?p/?q
1?p/?q
20qter
6
1
20
c20
20q13
HiC
Copy Number
A
B
Chr6
chr6
chr1
chr20
Chr20
Chr1

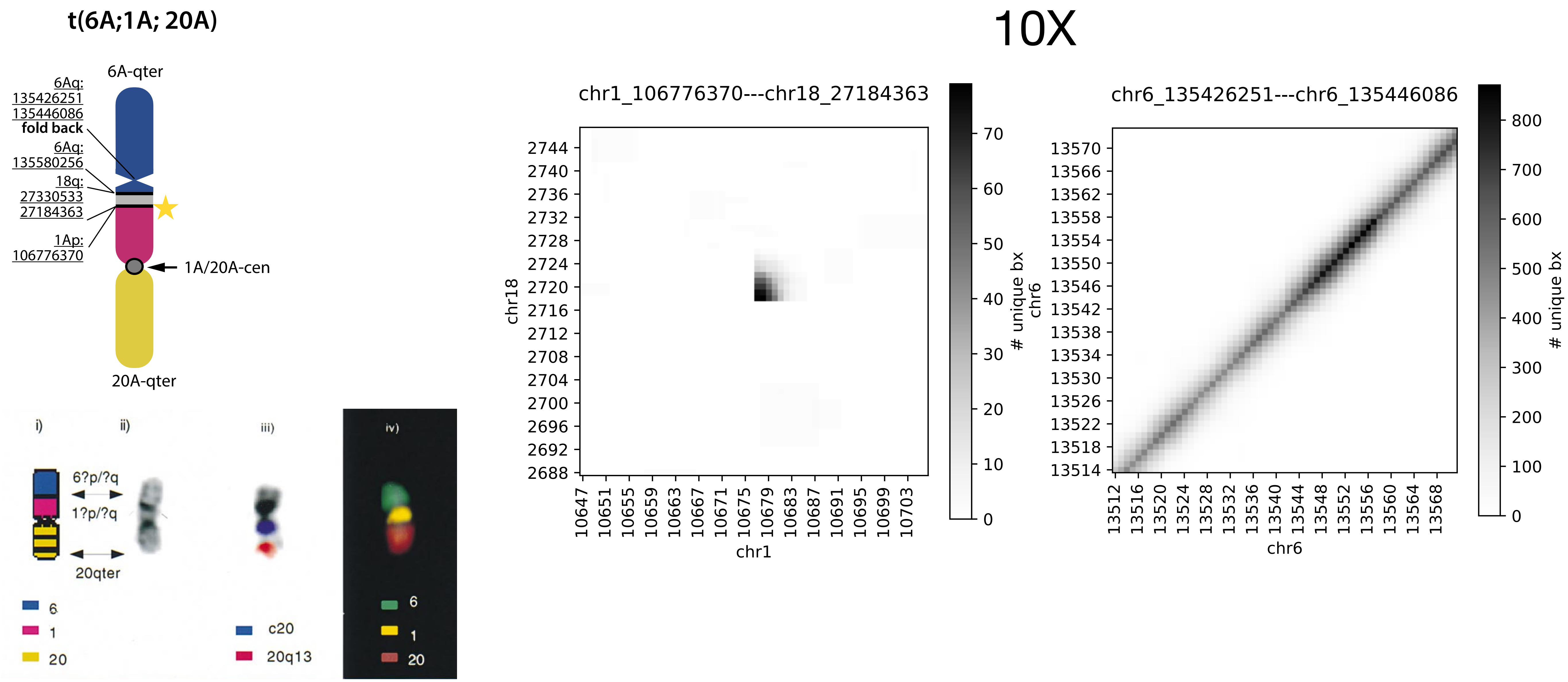

t(6A;1A; 20A)
6A-qter
6Aq:
135426251
135446086
fold back
6Aq:
135580256
18q:
27330533
27184363
1Ap:
106776370
1A/20A-cen
20A-qter
i)
ii)
iii)
iv)
6?p/?q
1?p/?q
20qter
6
1
20
c20
20q13
10X
chr1_106776370---chr18_27184363
chr18
chr1
unique bx
chr6_135426251---chr6_135446086
chr6

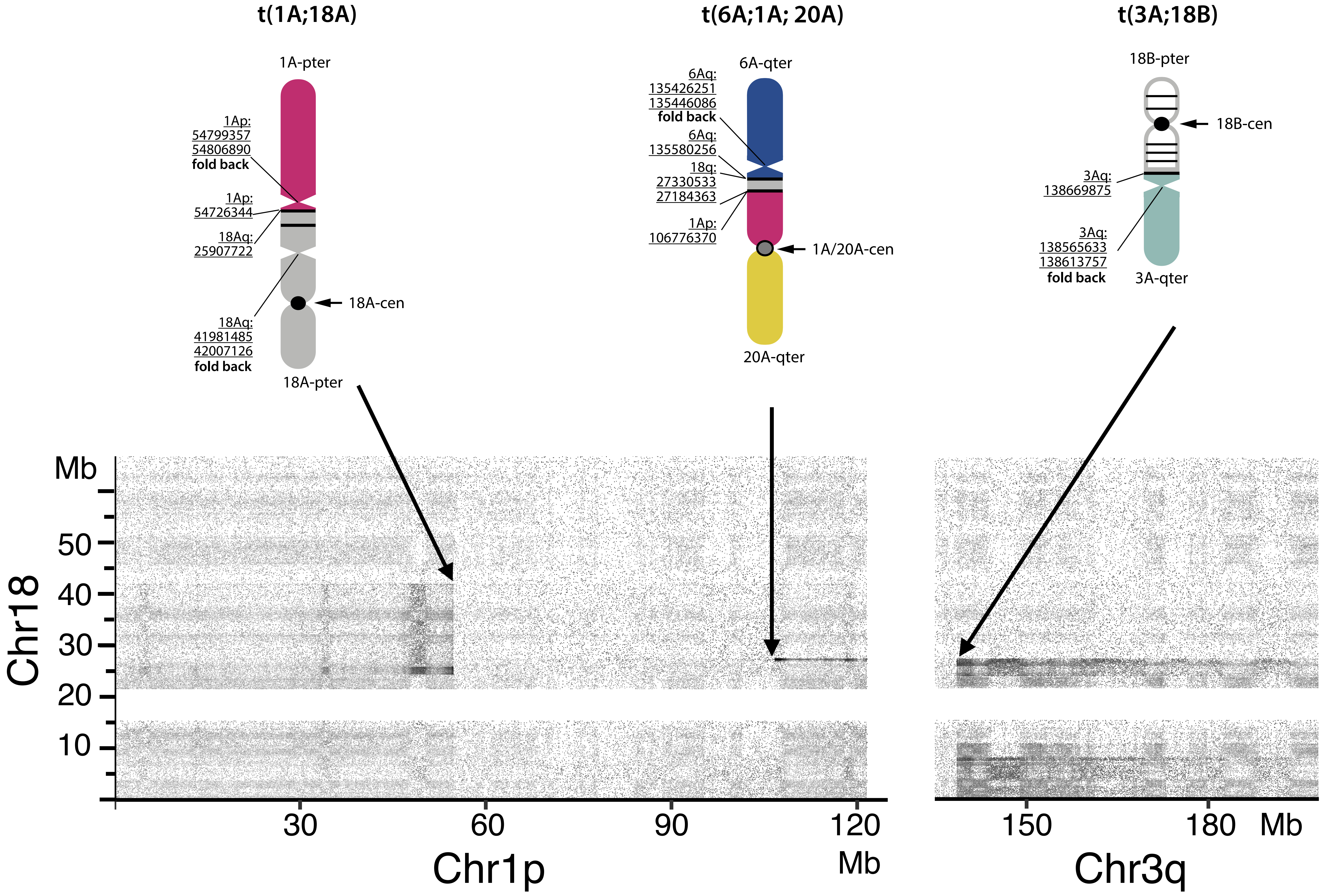
t(1A;18A)
1A-pter
1Ap:
54799357
54806890
fold back
1Ap:
54726344
18Aq:
25907722
18A-cen
18Aq:
41981485
42007126
fold back
18A-pter
t(6A;1A; 20A)
6A-qter
6Aq:
135426251
135446086
fold back
6Aq:
135580256
18q:
27330533
27184363
1Ap:
106776370
1A/20A-cen
20A-qter
t(3A;18B)
18B-pter
18B-cen
3Aq:
138669875
3Aq:
138565633
138613757
fold back
3A-qter
Mb
Chr18
50
40
30
20
10
30
60
90
120
Mb
Chr1p
150
180
Mb
Chr3q

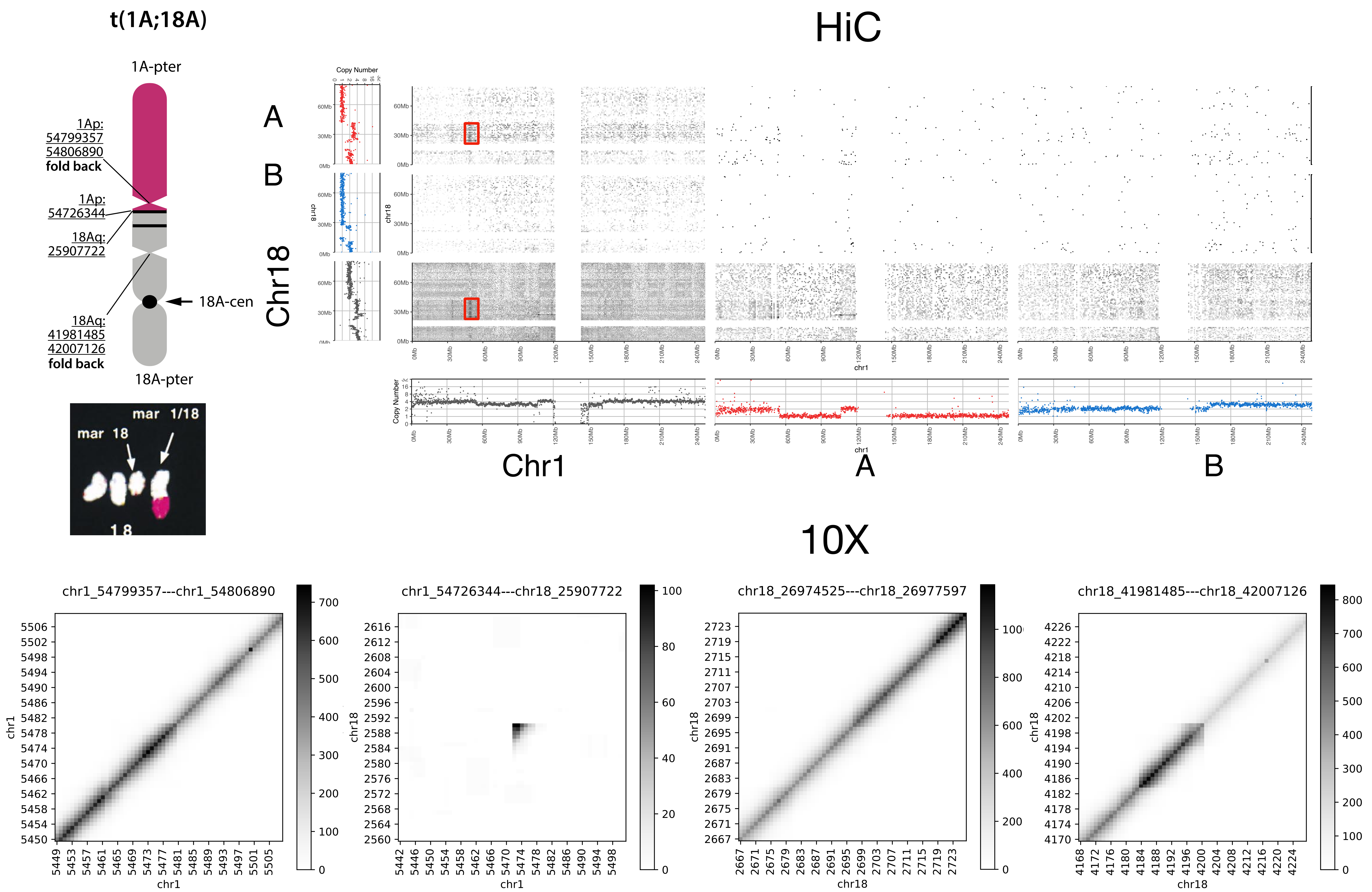

t(1A;18A)
1A-pter
1Ap: 54799357 54806890 fold back
1Ap: 54726344
18Aq: 25907722
18A-cen
18Aq: 41981485 42007126 fold back
18A-pter
mar 18
mar 1/18
18
HiC
A
B
Chr18
Copy Number
Chr1
A
B
10X
chr1_54799357---chr1_54806890
chr1_54726344---chr18_25907722
chr18_26974525---chr18_26977597
chr18_41981485---chr18_42007126

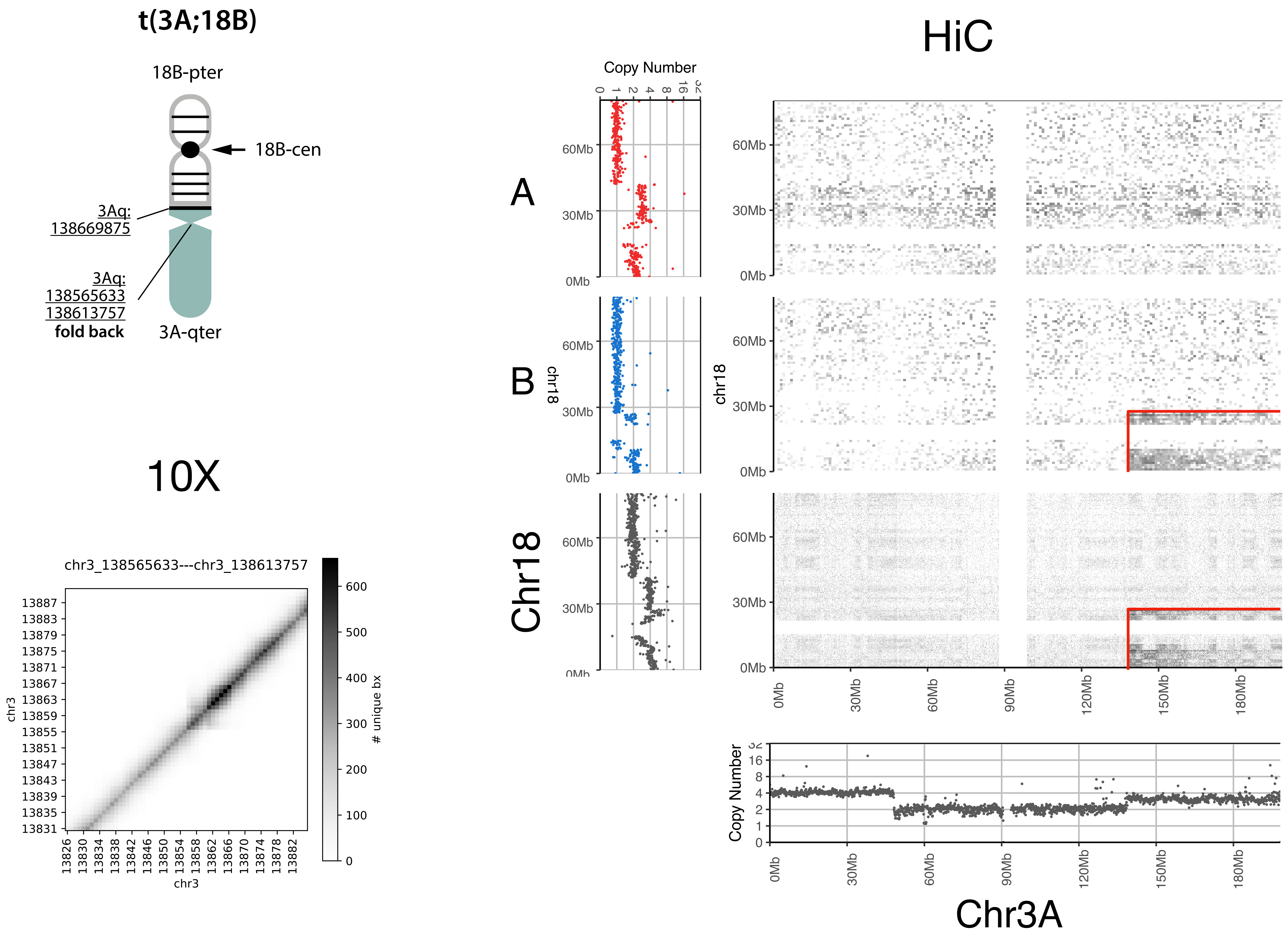

t(3A;18B)
18B-pter
18B-cen
3Aq:
138669875
3Aq:
138565633
138613757
fold back
3A-qter
10X
chr3_138565633---chr3_138613757
chr3
unique bx
HiC
Copy Number
A
B
Chr18
chr18
Chr3A

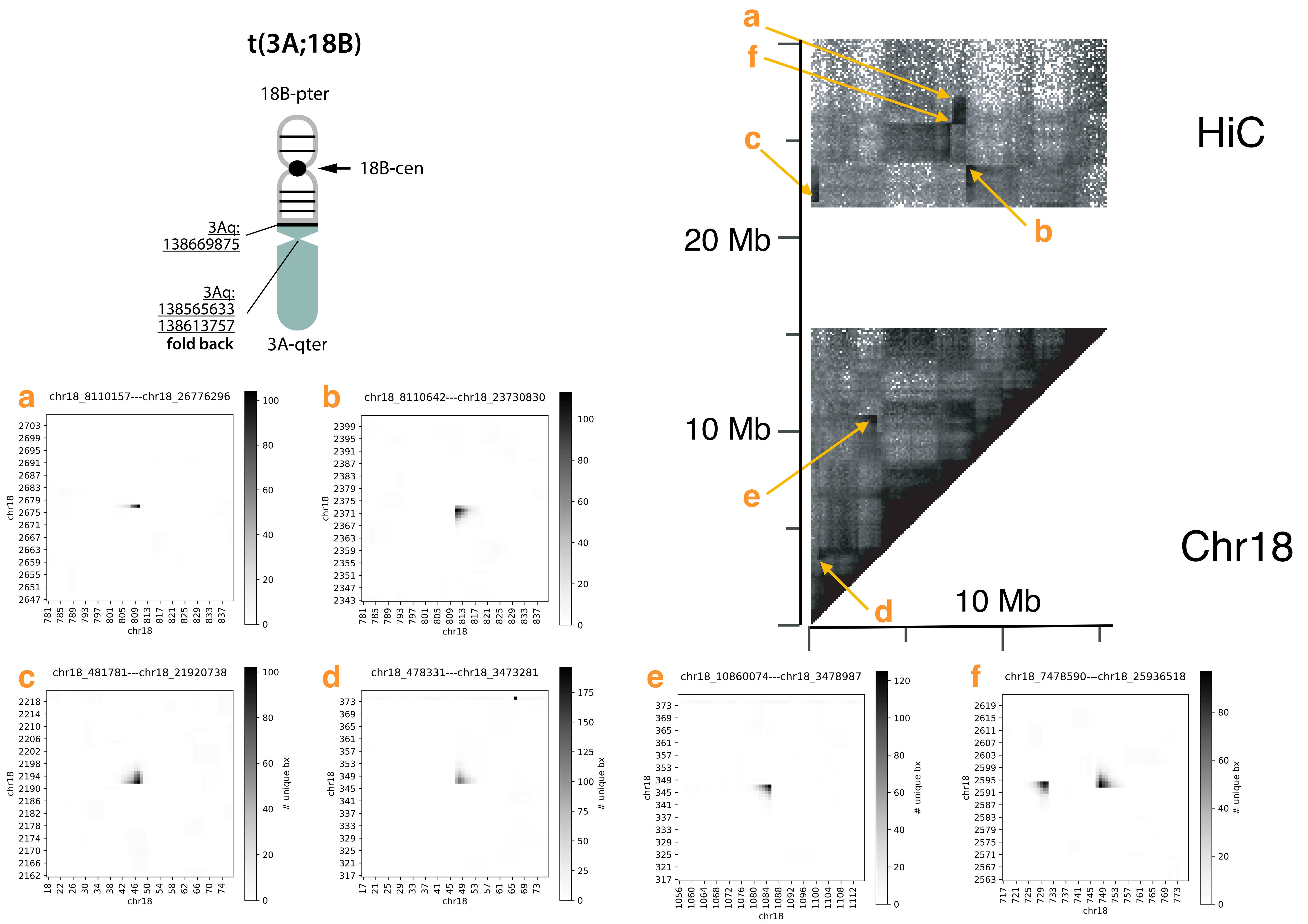

t(3A;18B)
18B-pter
18B-cen
3Aq:
138669875
3Aq:
138565633
138613757
fold back
3A-qter
HiC
Chr18
20 Mb
10 Mb
10 Mb
a
b
c
d
e
f
chr18_8110157---chr18_26776296
chr18_8110642---chr18_23730830
chr18_481781---chr18_21920738
chr18_478331---chr18_3473281
chr18_10860074---chr18_3478987
chr18_7478590---chr18_25936518
chr18
unique bx

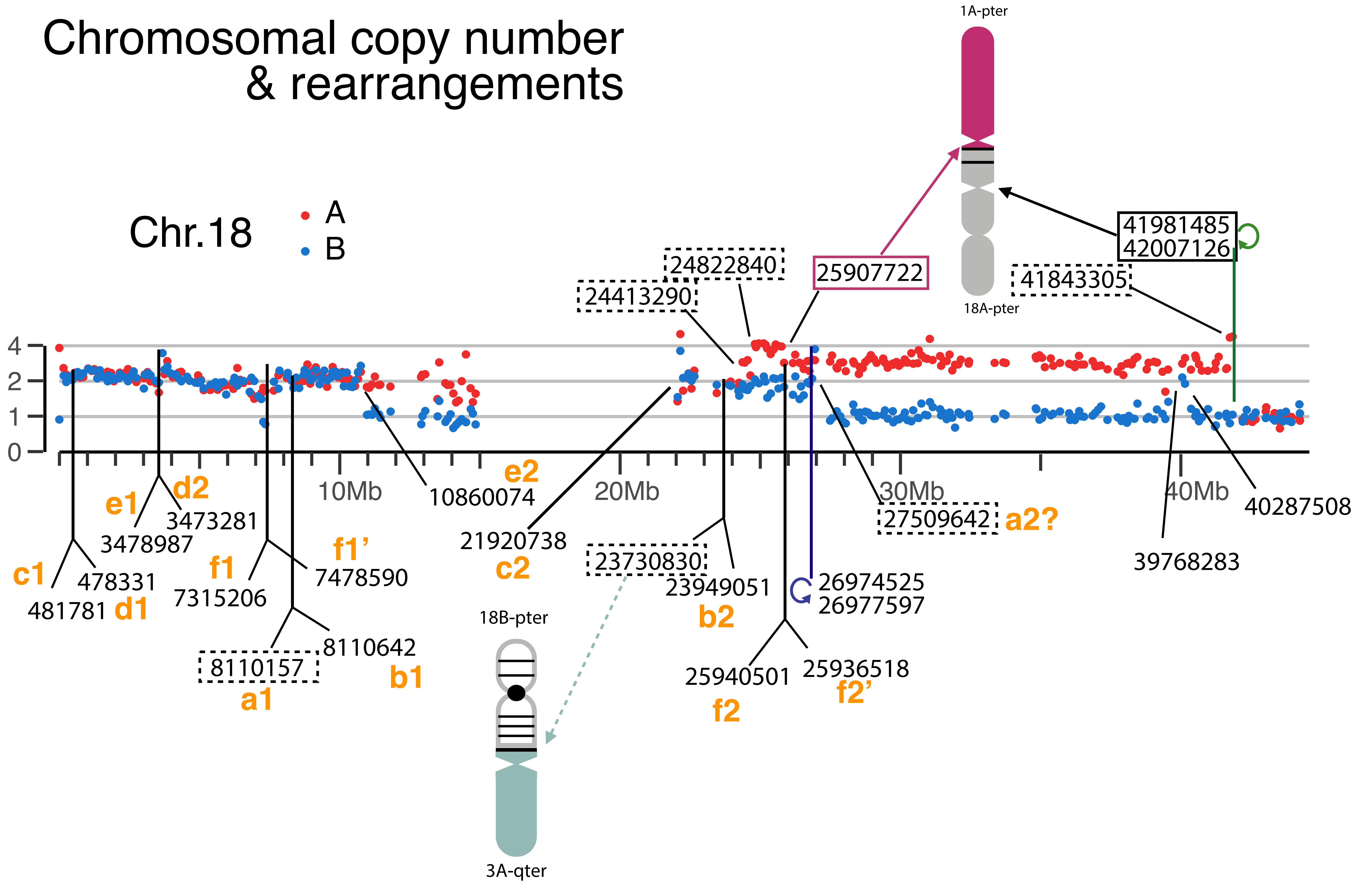
Chromosomal copy number
& rearrangements
Chr.18
A
B
1A-pter
18A-pter
18B-pter
3A-qter
10Mb
20Mb
30Mb
40Mb
0
1
2
4
41981485
42007126
41843305
25907722
24822840
24413290
10860074
21920738
23730830
23949051
27509642
26974525
26977597
25940501
25936518
39768283
40287508
3478987
3473281
478331
481781
7315206
7478590
8110157
8110642
a1
a2?
b1
b2
c1
c2
d1
d2
e1
e2
f1
f1'
f2
f2'

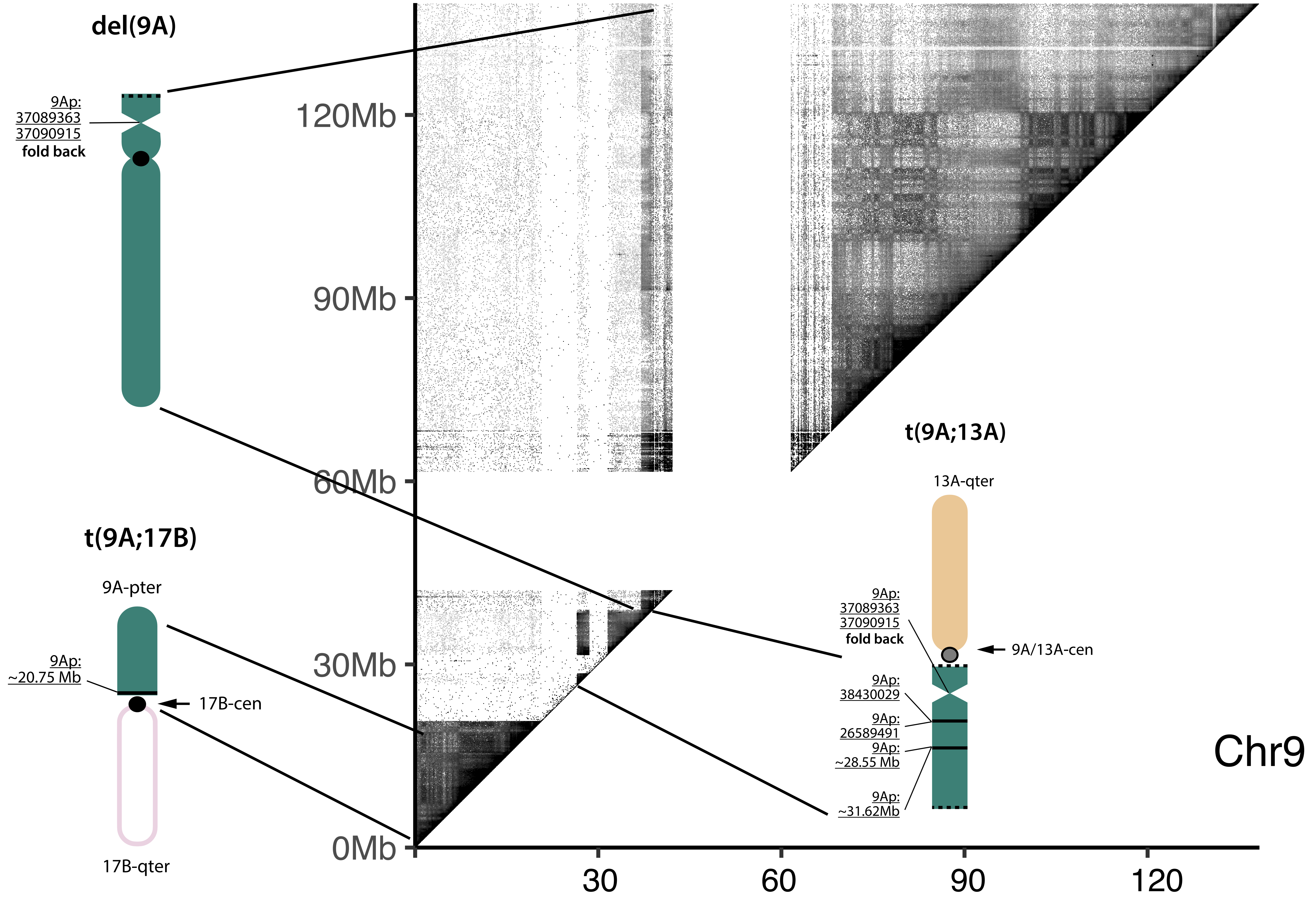

del(9A)
9Ap:
37089363
37090915
fold back
120Mb
90Mb
60Mb
30Mb
0Mb
t(9A;17B)
9A-pter
9Ap:
~20.75 Mb
17B-cen
17B-qter
t(9A;13A)
13A-qter
9Ap:
37089363
37090915
fold back
9A/13A-cen
9Ap:
38430029
9Ap:
26589491
9Ap:
~28.55 Mb
9Ap:
~31.62Mb
Chr9
30
60
90
120

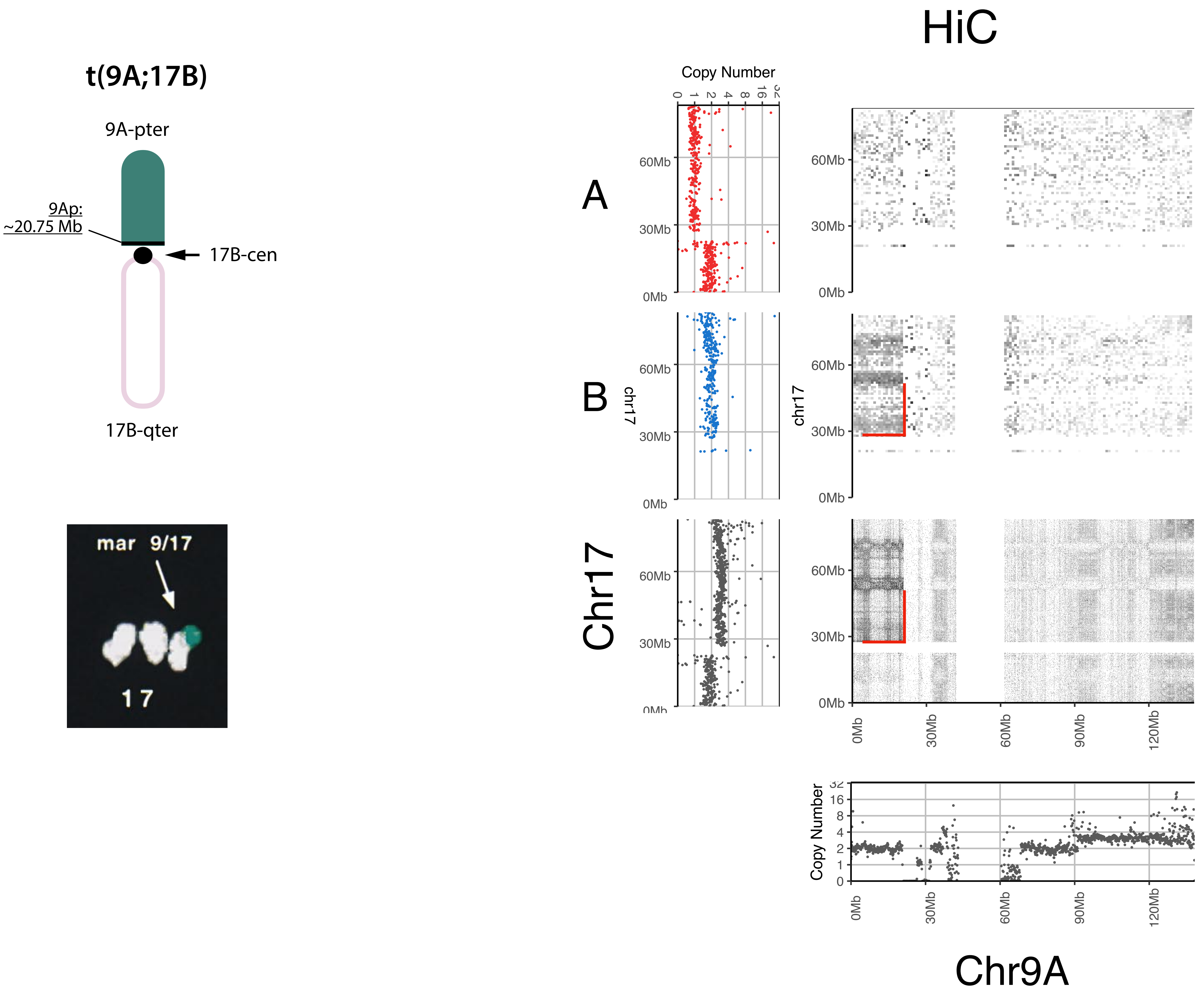
t(9A;17B)
9A-pter
9Ap:
~20.75 Mb
17B-cen
17B-qter
mar 9/17
17
HiC
Copy Number
A
B
Chr17
chr17
0Mb
30Mb
60Mb
90Mb
120Mb
0
1
2
4
8
16
32
Chr9A

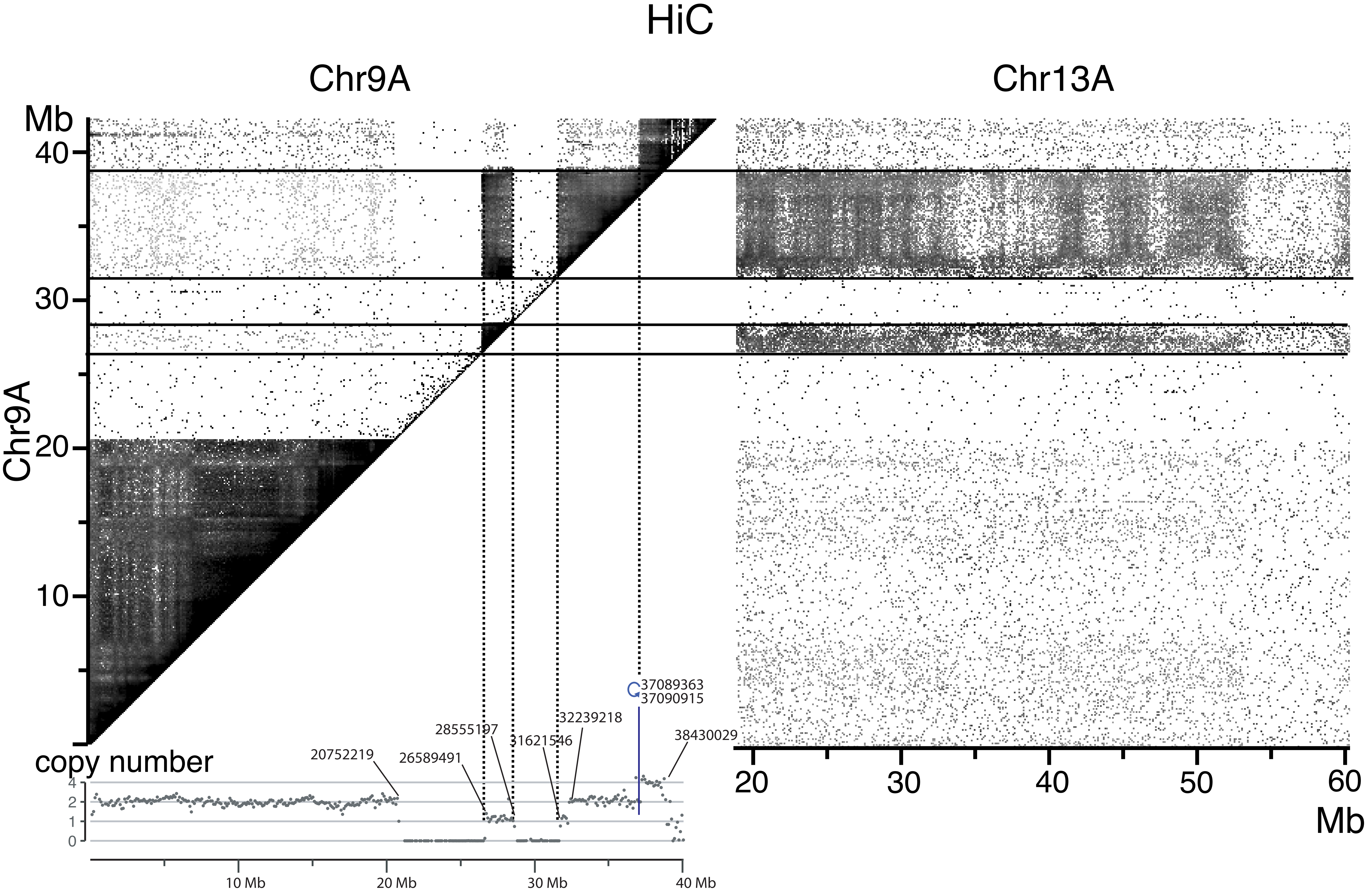

HiC
Chr9A
Chr13A
Mb
40
30
20
10
Chr9A
copy number
4
2
1
0
20752219
26589491
28555197
31621546
32239218
37089363
37090915
38430029
10 Mb
20 Mb
30 Mb
40 Mb
20
30
40
50
60
Mb

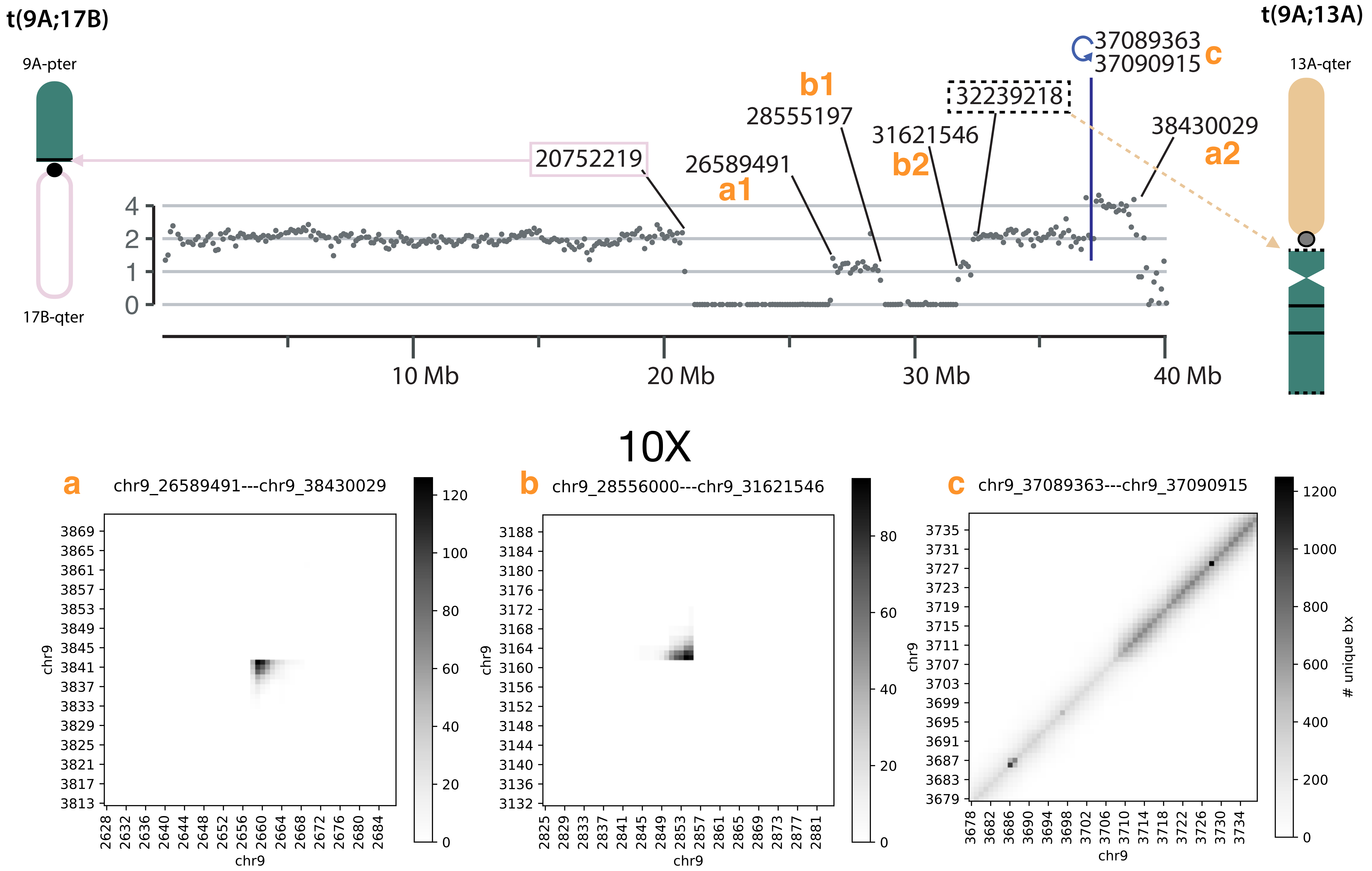

t(9A;17B)
9A-pter
17B-qter
t(9A;13A)
13A-qter
20752219
26589491
a1
28555197
b1
31621546
b2
32239218
37089363
37090915
c
38430029
a2
10 Mb
20 Mb
30 Mb
40 Mb
10X
a
chr9_26589491---chr9_38430029
b
chr9_28556000---chr9_31621546
c
chr9_37089363---chr9_37090915
chr9
unique bx

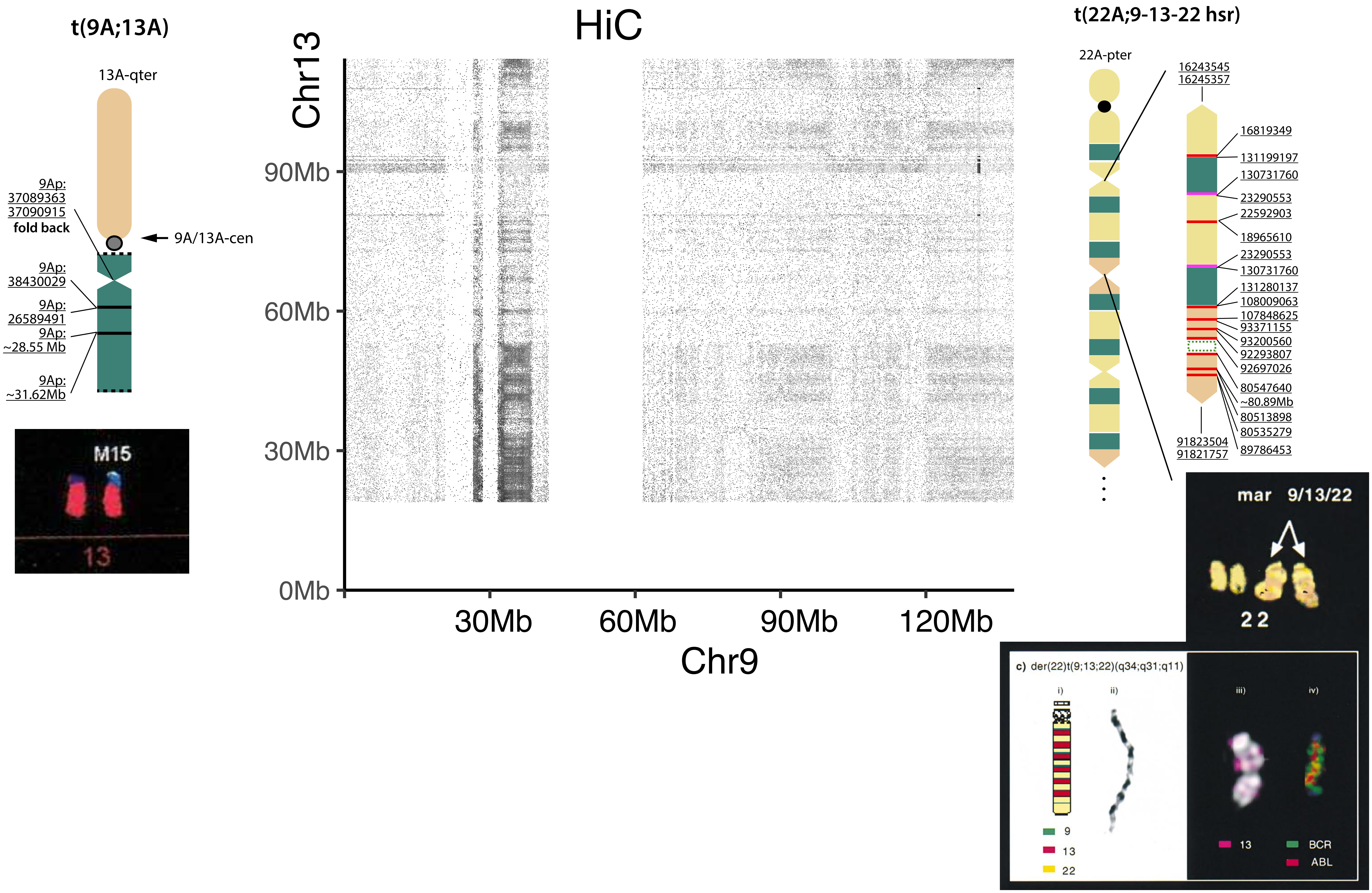

t(9A;13A)
13A-qter
9Ap:
37089363
37090915
fold back
9A/13A-cen
9Ap:
38430029
9Ap:
26589491
9Ap:
~28.55 Mb
9Ap:
~31.62Mb
M15
13
HiC
Chr13
90Mb
60Mb
30Mb
0Mb
30Mb
60Mb
90Mb
120Mb
Chr9
t(22A;9-13-22 hsr)
22A-pter
16243545
16245357
16819349
131199197
130731760
23290553
22592903
18965610
23290553
130731760
131280137
108009063
107848625
93371155
93200560
92293807
92697026
80547640
~80.89Mb
80513898
80535279
89786453
91823504
91821757
mar 9/13/22
22
c) der(22)t(9;13;22)(q34;q31;q11)
i)
ii)
iii)
iv)
9
13
22
13
BCR
ABL

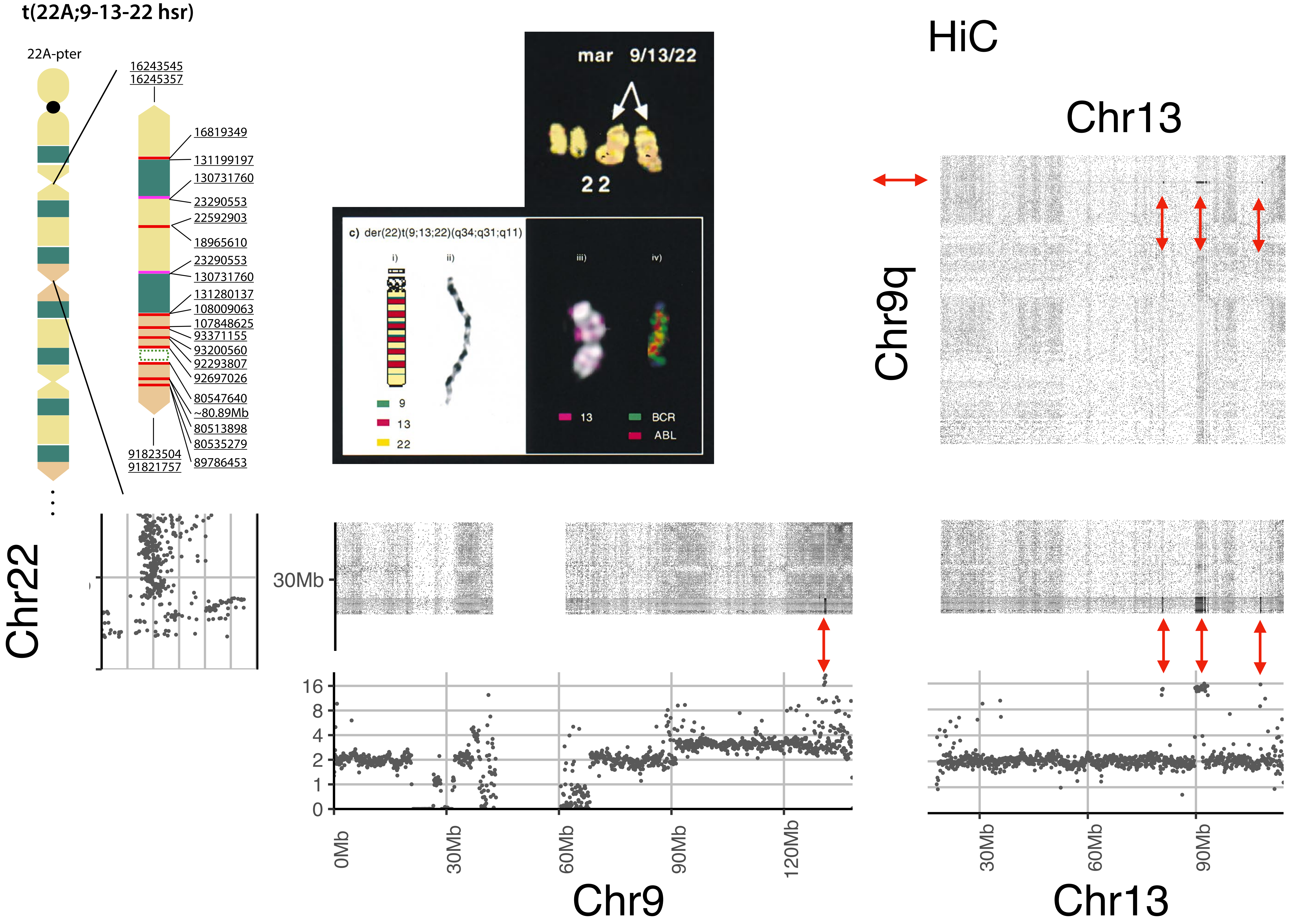

t(22A;9-13-22 hsr)
22A-pter
16243545
16245357
16819349
131199197
130731760
23290553
22592903
18965610
23290553
130731760
131280137
108009063
107848625
93371155
93200560
92293807
92697026
80547640
~80.89Mb
80513898
80535279
89786453
91823504
91821757
mar 9/13/22
22
c) der(22)t(9;13;22)(q34;q31;q11)
i)
ii)
iii)
iv)
9
13
22
13
BCR
ABL
HiC
Chr13
Chr9q
Chr22
30Mb
16
8
4
2
1
0
0Mb
30Mb
60Mb
90Mb
120Mb
Chr9
30Mb
60Mb
90Mb
Chr13

t(22A;9-13-22 hsr)

10X

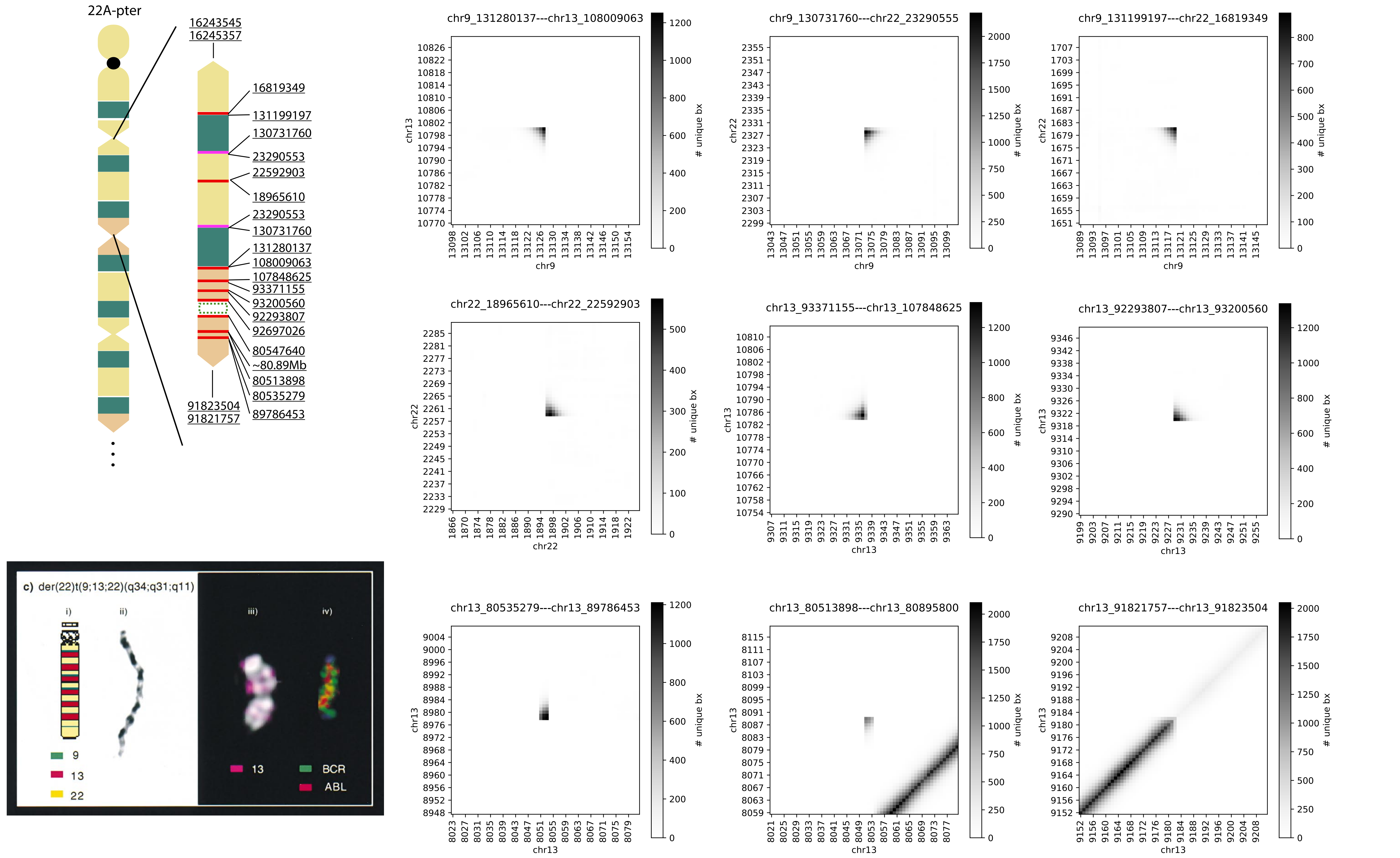

Supplement: Supplementary file 5 — Additional file 5 Please refer to Additional file 1:Determination of the K-562 karyotype by haplotype-specific genomic analysis for a detailed explanation. [file 13059_2021_2330_MOESM5_ESM.pdf]
