# Supplementary material for: Determination of complete chromosomal haplotypes by bulk DNA sequencing
Source: Genome Biol. 2021 May 6;22:139. doi: 10.1186/s13059-021-02330-1 (PMC8101039; doi:10.1186/s13059-021-02330-1)
Supplement: Supplementary file 8 — Additional file 8 Review history. [file 13059_2021_2330_MOESM8_ESM.docx]

Review History

**First round of review**

**Reviewer 1**

**Are you able to assess all statistics in the manuscript, including the appropriateness of statistical tests used?**

Yes: Please see comments below.

**Comments to author:**

The authors of this paper describe a computational strategy to determine whole-chromosome haplotypes for diploid genomes using a combination of

linked-reads and Hi-C sequencing. The strategy used by the authors first infers haplotype blocks using linked-reads and then merges these blocks into

chromosomal haplotypes using Hi-C data. My comments and concerns are listed below:

1. My main concern about this paper is that the idea of combining sequence data from different technologies to infer chromosomal-scale haplotypes is not new. Porubsky et al. (Nature Comm. 2017) combined Strand-seq data with long-read/linked-read data to show that this can be done with high accuracy. Similar results were obtained by Chaisson et al. (Nature Comm. 2019) using a combination of Hi-C/Strand-seq with long-reads/linked-reads. Even before that, Edge et al. (Genome Research 2016) had shown that combining Hi-C data with 10X genomics linked-reads results in highly complete and accurate haplotypes for a human genome.

2. The authors use a custom method for computational inference of haplotypes. Although the authors 'benchmark' the accuracy of their method, there are number of

other computational tools for haplotype phasing from sequence reads: Whatshap, hapcut2, etc. It is important to benchmark the accuracy of the authors' method

against previously published methods.

3. I would also suggest that the authors look into and refer previous papers on integrated haplotype phasing: Shay Ben-Elazar et al. 2016 (https://academic.oup.com/bioinformatics/article/32/17/i559/2450783) and Bansal 2019 (https://www.ncbi.nlm.nih.gov/pubmed/31510646), that are directly relevant to the authors' work.

4. It would have been interesting if the authors had looked into the coverage/depth requirements for obtaining dense, chromosome-scale haplotypes. This is useful for b

iologists who would like to use this strategy.

5. The DNA copy number analysis of the RPE-1 cell line are certainly novel, however, it is not clear whether chromosome-level haplotypes are required for this analysi

s. There have been several papers published that use linked-read data and haplotypes to analyze copy number alterations in cancer cell lines (Bell et al. https://academic.oup.com/nar/article/45/19/e162/4082855).

**Reviewer 2**

**Are you able to assess all statistics in the manuscript, including the appropriateness of statistical tests used?**

Yes: yes

**Comments to author:**

The authors present a "neat" computational strategy to phase genetic variants and group into phase blocks using long-range sequencing methods, linked-reads and Hi-C. The manuscript is delicately written and easy to follow. It has the advantage of using low-quality variant calls as inputs to begin with over conventional methods, e.g. HapCut2. Linking variants to increase the phase block and estimating ploidy is a unique feature, which makes Linker applicable for cancer genomes. The manuscript is in overall well written. All my comments are minor except one suggestion.

One way to evaluate phased variants being reliable is to make a consensus by mutating the reference sequence with the phased variants and evaluate phasing completeness and switch error rate with identified haplotype specific kmers. One drawback of mapping-based phasing approach is the uncertainty of the underlying variant callings, either caused by mapping biases or low sensitivity of the variant caller (false negatives). By mutating the reference with the phased variants, it becomes easier to evaluate whether the phased representation truly represents the sequenced individual. In addition to the GIAB benchmark for NA12878, it would be interesting to add k-mer based phasing evaluation statistics such as provided by Merqury or Yak. This way, readers can get a clearer view on which part of the genome is 'phase-able' using linked-reads and Hi-C only, without any long-reads.

Minor comments

1. Is it possible to provide a desired intra-block accuracy to define phase blocks?

2. Add the SAM in Fig. 4 legend. It is stated in the main text, however missing in the figure legend.

3. What is a "Marker" chromosome? Definition of the marker chromosome is missing in the main text and in Fig. 6.

4. In Fig. 6A, make the bars representing chromosome segments as arrows so the directionality can be understood.

5. Has it been evaluated to use short indels as targets for phasing, in addition to the bi-allelic SNPs? This will increase the density of variants. I expect the blocks may become larger and more accurate.

6. In the discussion, add limitations of this approach more in detail. Mapping based phasing methods obviously suffer on regions where haplotypes are highly diverged from the reference, where read mappings or variant callings will miserably fail. If possible, add the highest level of heterozygosity which Linker could reliably phase.

7. The Linker tool itself supports long-reads as input. How does the phasing performance differ when using long reads, e.g. CLR, HiFi, or ONT? Using long-reads will certainly help phase some of the difficult regions, by overcoming the mapping biases.

**Authors Response**

**Point-by-point responses to the reviewers’ comments:**

Reviewer #1: The authors of this paper describe a computational strategy to determine whole-chromosome haplotypes for diploid genomes using a combination of linked-reads and Hi-C sequencing. The strategy used by the authors first infers haplotype blocks using linked-reads and then merges these blocks into chromosomal haplotypes using Hi-C data. My comments and concerns are listed below:

1. My main concern about this paper is that the idea of combining sequence data from different technologies to infer chromosomal-scale haplotypes is not new. Porubsky et al. (Nature Comm. 2017) combined Strand-seq data with long-read/linked-read data to show that this can be done with high accuracy. Similar results were obtained by Chaisson et al. (Nature Comm. 2019) using a combination of Hi-C/Strand-seq with long-reads/linked-reads. Even before that, Edge et al. (Genome Research 2016) had shown that combining Hi-C data with 10X genomics linked-reads results in highly complete and accurate haplotypes for a human genome.

*We agree that the idea of combining different types of sequencing data to achieve long-range haplotype inference is not new. However, this idea is not the key innovation of our study. We consider the following results from our manuscript to be significant advancements over prior work.*

*a. We demonstrate that it is sufficient to determine the haplotype phase at the whole-chromosome scale without long-range switching errors using two bulk sequencing methods (linked-reads and Hi-C). In the revised manuscript, we have further demonstrated the combination of PacBio long-read sequencing and Hi-C sequencing can also achieve whole-chromosome scale haplotype inference. The most important aspect of our results is the "sufficiency" to achieve whole-chromosome haplotype inference with "no long-range switching error" using a combination of a long-molecule sequencing technology and Hi-C sequencing. None of the previous methods, including ones mentioned by the reviewers, demonstrated either "sufficiency" or "no long-range switching error" for their applications.*

*b. We showcase two applications of chromosome-specific copy number and Hi-C contacts to resolving complex rearrangements and karyotype changes. The examples demonstrate the feasibility to generate completely phased karyotypes of aneuploid cancer genomes using only sequencing data. In particular, we reconstructed the complete karyotype of the K-562 cell line using phased genomic data that recapitulates cytogenetic karytoypes. To our knowledge, this is the first direct demonstration that long-range cis-linkage can be used to infer the structure of rearranged chromosomes at the whole-chromosome scale.*

*The first two studies mentioned by the reviewer, Porubsky et al. (Nature Comm. 2017) and Chaisson et al. (Nature Comm. 2019) both required Strand-Seq data as input. As we discussed in both Introduction and Discussion, generating Strand-Seq data requires BrdU labeling of replicated DNA and therefore is only applicable to dividing cells. This is a significant barrier that cannot be overcome for applications to clinical samples. Furthermore, as Strand-Seq data already provide whole chromosome-scale haplotype information (though sparse), using strand-seq data for long-range haplotype inference is a simpler problem than using only Hi-C data. Due to these reasons, comparing our method against methods from these studies are not relevant as they are not applicable to the problem we want to address, i.e., generating complete chromosomal haplotypes using only bulk sequencing data.*

*The third study by Edge et al. (Genome Research 2016) is certainly related to our work. But there are at least two key differences. First, our computational method uses a completely different approach to incorporate long-range linkage from Hi-C data. This approach is critical for ensuring no long-range switching across an entire chromosome, even across large centromeric gaps (20-30Mb). By contrast, Edge et al. was mainly using Hi-C linkage to improve the accuracy and range of haplotype inference rather than completely eliminating long-range switching errors. When we ran HapCut2 on the same data, we observed sporadic long-range switching errors that significantly compromise the overall accuracy estimated using Hamming distance (Additional Data 5). By contrast, results from our method show both better completeness (i.e, more phased variants) and accuracy (maximum error rate per chromosome <=2%). Second, our computational method uses a simple heuristic model to estimate linkage accuracy between variant sites and therefore does not require input variants to be putative heterozygous variants. Our package also includes a variant filtration step (mlinker recover) that can be used to explicitly filter false variants with ambiguous haplotype linkage. The complete package can therefore take raw variant callset generated by a standard variant caller (GATK) and produce whole-chromosome haplotypes with >99% accuracy. This brings significant convenience for real applications as there is no well-established strategy for variant filtration. When we ran HapCut2 on the unfiltered variant callset (this is not recommended by the HapCut2 authors), its performance became significantly worse than when only high-quality variants are used. Finally, in comparison to the Edge et al. study, our haplotype inference method contains a module for self-consistency check that allows evaluation of haplotype accuracy even when the ground truth data are unknown (see Fig.S5). We think these advantages are significant improvements over the Edge et al. paper. In particular, we emphasize that demonstrating the feasibility to perform whole-chromosome haplotype inference using linked-reads plus Hi-C sequencing (which was mentioned in the Edge paper but not presented in detail) is different from demonstrating the sufficiency to generate whole-chromosome haplotypes with no long-range switching errors with these data.*

2. The authors use a custom method for computational inference of haplotypes. Although the authors 'benchmark' the accuracy of their method, there are number of other computational tools for haplotype phasing from sequence reads: Whatshap, hapcut2, etc. It is important to benchmark the accuracy of the authors' method against previously published methods.

*For reasons explained under Point 1, we think it is only fair and necessary to benchmark the performance of our method against HapCut2 by Edge et al. We emphasize that HapCut2 was designed to tackle the same problem (haplotype inference using a combination of long-read/linked-reads and Hi-C sequencing) but with a different purpose (to improve long-range haplotype inference but eliminating long-range switching errors completely). The design and implementation of our method is also different from HapCut2.*

*When we ran HapCut2 with high-quality variants (which was a requirement specified by the authors), the solution by HapCut2 showed similar average accuracy as the solution by mLinker, but had long-range switching errors in chr21 in the NA12878 genome and in chr19 in the RPE-1 data (average accuracy <90%).*

*When we used unfiltered variants as input (not recommended by HapCut2's authors), HapCut2 performed significantly worse. There were long-range switching errors in 3 chromosome (chr9, chr12, and chr21) in the NA12878 solution and in 5 chromosome (chr9, chr15, chr17, chr19, and chrX) in the RPE-1 solution.*

*In both benchmarking applications, the solutions from mLinker contained more phased variants and show better overall accuracy than the HapCut solutions.*

*Although the benchmark does show that our method is an improvement than HapCut2, we note that this improvement is specific to our application, i.e., to achieve complete whole-chromosome haplotype inference from deep linked-reads and Hi-C data. When long-range consistency is not mandatory and only low-depth data (10-20x) are available, HapCut2 may perform better in terms of completeness due to the more conservative approach we are taking.*

3. I would also suggest that the authors look into and refer previous papers on integrated haplotype phasing: Shay Ben-Elazar et al. 2016 (https://urldefense.proofpoint.com/v2/url?u=https-3A__academic.oup.com_bioinformatics_article_32_17_i559_2450783&d=DwIGaQ&c=vh6FgFnduejNhPPD0fl_yRaSfZy8CWbWnIf4XJhSqx8&r=CNNFLMxO4uNTPWvyDeZO8xce-3yyjwBs48Y1sSH5FuaUPZzC66Xz_B36lEUoRyrh&m=o5EyyShep9iQ3ppnjtWQeUQGfFQqiiX_25LplX6dZ2E&s=2nA1XzJnNLPWwyl3jeUsz1AhzOD4mIo0ReK9B0jlt-s&e= ) and Bansal 2019 (https://urldefense.proofpoint.com/v2/url?u=https-3A__www.ncbi.nlm.nih.gov_pubmed_31510646&d=DwIGaQ&c=vh6FgFnduejNhPPD0fl_yRaSfZy8CWbWnIf4XJhSqx8&r=CNNFLMxO4uNTPWvyDeZO8xce-3yyjwBs48Y1sSH5FuaUPZzC66Xz_B36lEUoRyrh&m=o5EyyShep9iQ3ppnjtWQeUQGfFQqiiX_25LplX6dZ2E&s=m4wlF1YfZKAVMCZrPSOyO222L405guRNOffRTA6Ps34&e= ), that are directly relevant to the authors' work.

*We thank the referee for bringing these two studies to our attention. The manuscript by Bansal (2019) uses local haplotypes derived from population haplotype data to refine haplotype inference using Hi-C data. The combination of population haplotype data and Hi-C linkage is certainly a powerful application. As a matter of fact, we mentioned about this extension of our formalism of haplotype inference in Discussion without knowledge of this work:*

*"One useful extension of our method is to perform joint haplotype inference using population genotypes and Hi-C data. Population-based statistical phasing [16] can produce long haplotype blocks (>1Mb) that contain random but rare switching errors. It should be possible to correct these errors using Hi-C data and generate the complete haplotype phase of common variants on individual chromosomes, which can then be used to generate phased Hi-C contact maps.*

*We were not aware of this study when preparing our manuscript and have now cited this study properly. We also have a manuscript in preparation that addresses the same problem as in Bansal (2019). In this separate manuscript, we defined the problem using the same formalism as described in the current study, but came up with a different algorithm of haplotype solution given the different nature of statistically inferred haplotype linkage from population data.*

*The study by Ben-Elazar has now been appropriately cited in our study.*

*"In contrast to previous methods that perform joint haplotype inference using linkage information from different technologies [..], ..."*

*Although this study set out to address several problems that are being addressed in our manuscript (including both haplotype inference using Hi-C data and generating haplotype-specific Hi-C contacts), it was more a proof-of-principle test than solving a real bioinformatic problem with well-calibrated benchmarks. We therefore do not think an in-depth discussion of this study is necessary.*

4. It would have been interesting if the authors had looked into the coverage/depth requirements for obtaining dense, chromosome-scale haplotypes. This is useful for biologists who would like to use this strategy.

*Following the suggestion by the reviewer, we have used our method to perform haplotype inference on down-sampled linked-reads and Hi-C data of the RPE-1 sample. The results are summarized in Table S5 with detailed benchmarks for each chromosome provided in Additional Data Table 4 — Additional benchmark metrics of the haplotype solution of the RPE-1 genome. We further added Figure S5 and additional discusson on how the depth of sequencing data (linked reads) and the choice of switching energy cutoff impact local haplotype inference. To briefly summarize the result of this analysis, we have found that using >=50% linked-reads and >=50% Hi-C reads of the original data, our method can generate whole-chromosome haplotypes with >99% accuracy and >97% completeness relative to the original haplotype solution. This result is generated using variants detected by the original linked-reads data and therefore only reflects haplotype inference but not variant detection.*

5. The DNA copy number analysis of the RPE-1 cell line are certainly novel, however, it is not clear whether chromosome-level haplotypes are required for this analysis. There have been several papers published that use linked-read data and haplotypes to analyze copy number alterations in cancer cell lines (Bell et al. https://urldefense.proofpoint.com/v2/url?u=https-3A__academic.oup.com_nar_article_45_19_e162_4082855&d=DwIGaQ&c=vh6FgFnduejNhPPD0fl_yRaSfZy8CWbWnIf4XJhSqx8&r=CNNFLMxO4uNTPWvyDeZO8xce-3yyjwBs48Y1sSH5FuaUPZzC66Xz_B36lEUoRyrh&m=o5EyyShep9iQ3ppnjtWQeUQGfFQqiiX_25LplX6dZ2E&s=SfNGx_zqzTPABTSVgn1GSt6j1gDYjTKbasxtzVluOgs&e= ).

*We agree with the reviewer that knowledge of whole-chromosome haplotype is not always necessary to derive allele-specific DNA copy number. (For this application, we also have a separate manuscript in preparation.) However, whole-chromosome haplotype will give the most definitive results on chromosome-specific DNA copy number alterations, including linking copy-number alterations on opposite chromosome arms.*

*The basic idea of allele-depth-based phasing is that each copy-number breakpoint is specific to one parental chromosome, and therefore the intact homolog will have constant allelic depth across a copy-number changepoint. Allelic-depth based phasing works well when there is whole-chromosome allelic imbalance, but cannot extend through regions with allelic balance. It is also not as useful when both parental chromosomes have multiple segmental copy-number changes on the same region.*

*The chromosome-specific copy number data of aneuploid RPE-1 cells presented here is calculated directly using haplotype-specific coverage and therefore does not make any assumption about whether copy-number changes affect one or both homologs. Moreover, in the two examples shown in Fig. 5B and 5C, both parental chromosomes have segmental copy-number alterations that can only be correctly resolved with knowledge of the complete haplotype. In Fig.5B, the segmental gain of the red homolog can be incorrectly assigned to the blue homolog as the flanking pericentric region is in allelic balance. In Fig. 5C, the varying copy number at the q-terminus is reflective of genetic variation between cells; this pattern is only evident when comparing the varying coverage of the blue homolog against the constant coverage of the red homolog.*

Reviewer #2: The authors present a "neat" computational strategy to phase genetic variants and group into phase blocks using long-range sequencing methods, linked-reads and Hi-C. The manuscript is delicately written and easy to follow. It has the advantage of using low-quality variant calls as inputs to begin with over conventional methods, e.g. HapCut2. Linking variants to increase the phase block and estimating ploidy is a unique feature, which makes Linker applicable for cancer genomes. The manuscript is in overall well written. All my comments are minor except one suggestion.

*We thank the reviewer for his/her enthusiasm about our work and the favorable comment with regard to our method in comparison to other methods including HapCut2.*

One way to evaluate phased variants being reliable is to make a consensus by mutating the reference sequence with the phased variants and evaluate phasing completeness and switch error rate with identified haplotype specific kmers. One drawback of mapping-based phasing approach is the uncertainty of the underlying variant callings, either caused by mapping biases or low sensitivity of the variant caller (false negatives). By mutating the reference with the phased variants, it becomes easier to evaluate whether the phased representation truly represents the sequenced individual. In addition to the GIAB benchmark for NA12878, it would be interesting to add k-mer based phasing evaluation statistics such as provided by Merqury or Yak. This way, readers can get a clearer view on which part of the genome is 'phase-able' using linked-reads and Hi-C only, without any long-reads.

*We thank the reviewer for this suggestion and agree that this is in principle an orthogonal strategy to validate the accuracy of haplotype inference from aligned reads. With help from Heng Li, the author of Yak, we had attempted to carry out the analysis suggested by the reviewer with some degree of success. In performing this analysis, we also revised the approach suggested by the reviewer to better address the question raised by the reviewer.*

*Heng Li kindly provided the data of parent-specific k-mers (k=31) generated from the parents of the NA12878 individual (Father: NA12891; Mother: NA12892). We constructed the DNA sequence of parental chromosomes in the NA12878 genome by incorporating phased single-nucleotide variants into the reference sequence (as suggested by the reviewer). The evaluation of phasing accuracy was done using the following command suggested by Heng Li:*

*yak trioeval -t16 NA12891-pat.PG.k31.yak NA12892-mat.PG.k31.yak .fa*

*Results from this analysis is presented in "Additional Data Table 3 — Additional benchmark metrics of the haplotype solution of the NA12878 genome." We observe that the parent-specific k-mers matching each artificial parental chromosome sequence is dominated by one parent. However, there is still a sigificant fraction of k-mers from the opposite parent that match the same artificial parental chromosome. We obtain very similar metrics when using the GIAB haplotype.*

*We think this result is caused by non-SNV variants (insertion/deletion events and structural variants) on both parental chromosomes of NA12878. In constructing the parental chromosome sequences using phased SNV sites, we have used the reference sequence to represent all non-SNV variants on both parental chromosomes. When the k-mers derived from the artificial parental chromosome sequences are compared against parent-specific k-mers generated from the parents' sequencing reads, the reference sequence at non-SNV variant sites in the artificial parental chromosome sequences can cause ambiguous matching to parent-specific k-mers. For example, if the child inherited the father's chromosome that had an indel and the mother's chromosome with the reference sequence, then the reference sequence being used in the artificial chromosomes will always match a unique k-mer in the father's genome. Therefore, the counts of parent-specific k-mers identified in the artificial parental chromosome sequences constructed from phased SNV sites do not faithfully capture the accuracy of phased SNVs.*

*To overcome this problem, we constructed and compared haplotype-specific k-mers near phased SNV sites in the NA12878 genome against parent-specific k-mers constructed from the parents' genomes. In doing so, we are limiting the comparison to phased SNV sites and evaluating the specificity of phasing at these sites. Since non-SNV variants are not included in the haplotype phase, they are also not included in this comparison. We think this comparison essentially addresses the reviewer's request for an independent validation of phasing accuracy without resorting to reference alignment.*

*For each phased variant, we separately constructed 35-mers for the alternate and the reference genotypes at each variant site and assessed their matching to parent-specific 31-mers identified using yak. There are multiple possible scenarios. If the reference allele and the alternate allele are completely segregated in the parents (e.g., one parent is homozygous reference, the other is homozygous alternate), we expect that both alternate and reference k-mers to match parent-specific k-mers. If the reference allele and the alternate allele are partially segregated (e.g., one parent is heterozygous, the other is homozygous reference or homozygous alternate), then we expect either the reference or the alternate allele to match with one parent-specific k-mer. If both parents show heterozygous genotypes, then neither the reference nor the alterlate allele will match a parent-specific k-mer. For variants that show complete segregation of genotypes in parents' genomes, we verify that the specificity of phased variants exceeds 99% for every chromosome. These variants comprise 13-18% of all phased variants on each autosome, and 36% of all phased variants on ChrX. For variants matching with k-mers in one parent, the specificity of haplotype phase is comparable to variants with completely segregated alleles, with chr19 showing the lowest specificity of 98.5%. For 25-32% of variants on autosomes, we find no matching of either reference or alternate alleles with parent-specific 31-mers. We think a large fraction of these variants are heterozygous in both parents.*

*A special case is the X chromosome, as the paternal X is hemizygous in the father's genome. Of all high-quality phased variants on the X chromosome, 35.6% are found to have complete segregation of reference and alternate alleles to both parents: we think most of these variants are homozygous in the mother's genome. 46.5% variants have only one allele that is specific to the mother's genome: these variants should be heterozygous in the mother's genome. Assuming the ratio of heterozygous genotypes to homozygous genotypes in the mother's genome to be similar for all chromosomes, we estimate that 56.7% of heterozygous variants in the child's genome will be also heterozygous in the mother's genome. If we further assume a similar ratio for variants on autosomes in the father's genome, then we expect the percentage of heterozygous variants in the child's genome that is also heterozygous in both parents to be 0.567^2=0.32, which is comparable to the percentage of variants with no matching parent-specific k-mers.*

*Although we think the above analysis adequately addresses the reviewer's request, we performed an additional validation that is perhaps more informative. We obtained phased variants in the NA12878 genome from Heng Li that were determined directly from the sequences of parental chromosomes obtained by de novo diploid assembly. We used this dataset as an additional reference data to the GIAB reference and included benchmarks against this data in Table 2, S2, and "Additional Data Table 3 — Additional benchmark metrics of the haplotype solution of the NA12878 genome." Using this new reference data, we now confirmed the accuracy of our haplotype solution for the NA12878 genome outside the confident regions in the GIAB release.*

Minor comments

1. Is it possible to provide a desired intra-block accuracy to define phase blocks?

We have introduced block-switching penalty as a measure of intra-block accuracy. This score depends on a couple of parameters, including local variant density, the range of molecular linkage, the frequency of sequencing errors, and the fraction of false variants. Although the range of molecular linkage and the frequency of sequencing errors can be assumed to be constant, the density of real and false variants can vary significantly across the genome. While choosing a low switching cutoff is sufficient to extend haplotype inference through most of the genome, doing so may incur sporadic switching errors that destroy long-range consistency. As our priority is to avoid any long-range switching error rather than to maximize the length of local haplotype blocks, we opt for a more conservative cutoff that results in shorter blocks in general.

*To assess how the choice of switching cutoff impacts the final haplotype solution (with Hi-C scaffolding),* *we have now evaluated the distribution of block-switching penalty in the local haplotype solution determined from both linked-reads and PacBio long-read data. For linked-reads data, including down-sampled data (40x, 30x, and 20x), we observe a local minimum in the switching energy distribution that is ~0.1 x median. This is a conservative cutoff that should always ensure intra-block accuracy in the haplotype solution. For the PacBio data, we do not see a local minimum, which may be due to either the low sequencing depth of the PacBio data (15x) or the shorter average length of PacBio reads in comparison to the molecular length of linked-reads data.*

*We then determined local haplotype blocks using different switching-energy cutoffs and calculated the percentage of variants in long local phase blocks (>=50 phased variants) with 98% phasing accuracy, the percentage of variants in long phase blocks (>=50 phased variants) but less than 98% accuracy, and the percentage of variants in short phase blocks (<50 variants). These results are summarized in Fig.S6B (linked reads) and S6C (PacBio). With low switching-energy cutoffs, there is a larger fraction of variants in long blocks with less accuracy; with high switching-energy cutoffs, the intra-block accuracy gets better but there is a larger fraction of variants in short phase blocks. Both of these compromise Hi-C based scaffolding of haplotype blocks that require long and accurate local blocks. If we use the fraction of variants in high-accuracy long phase blocks as a measure, then the optimal cutoff is estimated to be 50~100 for the linked-reads data. We note that this number will vary depending on the range of molecular linkage. To ensure the best accuracy of the final haplotype solution, we recommend choosing a more conservative cutoff to avoid any potential switching errors, especially those in low variant-density regions, as long as the N50 size of phase blocks is above 100 kb (to ensure specific Hi-C linkage).*

*We note that the local haplotype blocks determined from PacBio Hi-Fi reads have excellent accuracy (>98%) even with very low switching cutoffs. This reflects two advantages of PacBio Hi-Fi reads over short linked reads: better alignment accuracy and more specific haplotype linkage. These advantages make the estimation of switching errors more accurate. The PacBio haplotype solution was also generated using only high-quality variants, which eliminate ambiguous false linkage.*

*Finally, intra-block phasing accuracy can be assessed by the fraction of trans Hi-C links in the final haplotype solution even without knowledge of the true haplotype. (This is mentioned in Results and demonstrated in Fig.S5.) This feature will be useful for further optimization of the choice of intra-block accuracy.*

2. Add the SMA in Fig. 4 legend. It is stated in the main text, however missing in the figure legend.

*We have added this in the Figure Caption (highlighted in red).*

3. What is a "Marker" chromosome? Definition of the marker chromosome is missing in the main text and in Fig. 6.

*We have provided an explanation of "marker" chromosomes both in the main text and in the Fig.6 legend (highlighted in red).*

4. In Fig. 6A, make the bars representing chromosome segments as arrows so the directionality can be understood.

*We have added the arrows per suggestion by the reviewer.*

5. Has it been evaluated to use short indels as targets for phasing, in addition to the bi-allelic SNPs? This will increase the density of variants. I expect the blocks may become larger and more accurate.

*The main challenge for incorporating indel variants in haplotype phasing is to eliminate false variants and accurately genotype true variants in sequencing reads. Given that (1) SNVs have overall better detection accuracy than indels; (2) the density of SNVs is significantly higher than the density of indels; and (3) we can already generate whole-chromosome haplotypes of SNVs, we do not think including indel variants in haplotype inference is necessary for generating whole-chromosome haplotype phase. However, we think the following two applications are useful: (1) adding true indel variant genotypes to the SNV haplotype to increase local variant density; (2) filtering false indel variants using haplotype linkage between indel variant genotypes and phased SNV genotypes. We have now demonstrated these applications by performing haplotype inference on indel variants on chr.21 of the NA12878 genome and benchmark the results against both the GIAB reference (alignment-based) and the reference from de novo diploid assembly. These results are summarized in the new Table 2. The high concordance between the indel haplotype determined from haplotype linkage and the reference indel haplotype suggests that our method can phase true indel variants with great accuracy. We further note that using the haplotype-linkage filter, we have excluded a large number of variants (about 1/3) in the original callset from HaplotypeCaller, most of which are not present in either reference data and therefore likely to be false variants.*

6. In the discussion, add limitations of this approach more in detail. Mapping based phasing methods obviously suffer on regions where haplotypes are highly diverged from the reference, where read mappings or variant callings will miserably fail. If possible, add the highest level of heterozygosity which Linker could reliably phase.

*We have added discussion on the limitations of haplotype inference from aligned short reads. For regions that diverge significantly from the reference, the best approach should be de novo assembly rather than reference-based alignment. As a computational method designed primarily for alignment-based analysis, our algorithm excludes these regions rather than attempting to extend haplotype inference into these regions.*

*The limited range of molecular linkage from linked-reads data suggests that this datatype is suitable for local haplotype phasing but cannot extend haplotype blocks across regions with variant density lower than 1 per 100kb). To ensure reliable haplotype inference, our method will exclude large regions with variant density lower than 1 per 10kb. This is determined by the switching penalty.*

7. The Linker tool itself supports long-reads as input. How does the phasing performance differ when using long reads, e.g. CLR, HiFi, or ONT? Using long-reads will certainly help phase some of the difficult regions, by overcoming the mapping biases.

*The key parameters that determine phasing performance are the density of variants, the length range of molecular linkage, and the accuracy of genotypes. For PacBio data, the read length is in the range of 10-40kb, which is significantly shorter than linked reads (~100 kb). Nanopore reads can reach several megabases that exceed the maximum range of linked reads, but the average length is still shorter. Except for PacBio HiFi reads, other long reads data (PacBio CLR or Nanopore) generally have less base accuracy (10-15% error rate). Based on these considerations, we think linked-reads data offer the best overall performance (including consistency) of local haplotype inference.*

*To partially address the reviewer's question, we have now performed haplotype inference using a combination of PacBio HiFi data of RPE-1 cells and the same RPE-1 Hi-C data. Since the HiFi data have only ~15x median coverage, we do not expect this combination to achieve the same level of completeness as deep linked-reads data, but the design of our algorithm should ensure long-range consistency of the final haplotype. The result confirms our expectation. Even with 15x PacBio Hi-Fi data, our method can phase 90% of all input variants (all high-quality) with ~98% average accuracy. By contrast, HapCut2 achieved better completeness (94%) but with less accuracy (94%) and made an arm-level switching error in Chr.1. This comparison further demonstrates the different priority of our method than HapCut2.*

*PacBio Hi-Fi reads have two advantages over short reads: they have better alignment accuracy and more specific haplotype linkage than linked reads. These advantages make the estimation of switching errors more accurate. As a new result presented in Fig.S6C, we show that local haplotype blocks determined with a very low switching penalty score ~5 still show good (>98%) intra-block accuracy. By contrast, the penalty cutoff needs to be ~100 for linked-reads data to achieve the same level of intra-block accuracy. The specificity of haplotype linkage from HiFi PacBio reads is a clear advantage over linked reads.*

*The main limitation of PacBio Hi-Fi reads compared to linked reads is the shorter range of molecular linkage. The median length of Hi-Fi reads ~10kb is an order-of-magnitude lower than the molecular size of linked-reads data (~100 kb). This implies that PacBio reads cannot extend haplotype inference between two variants separated by >10kb. The caveat is that the low variant density in some regions is due to limited detection power of short reads and long PacBio reads may uncover variants in these regions that are not detectable by short reads.*

*While PacBio reads can resolve and extend haplotype inference into difficult regions, such as those that diverge significantly from the reference, we think this advantage is better utilized in phased de novo assembly of parental chromosomes. This problem has been partially addressed by a recent manuscript from Heng Li's group and is an orthogonal to the alignment-based approach undertaken here. A direct comparison of these two approaches is beyond the scope of the current manuscript.*

**Second round of review**

**Reviewer 1**

The authors have revised the manuscript significantly in response to the previous comments. They have highlighted that the main advance of their method is the ability to generate whole-chromosome haplotypes with no long-range switching errors and to do so even when the input set of variants has errors. Indeed, their method is designed to carefully build chromosome-scale haplotypes using long haplotype blocks and Hi-C data in order to avoid long-range errors. However, the abstract is very generic and does not reflect these specific advances of their method. Rewriting the abstract will help bring out the specific contributions of the method described in this paper. In addition, I have a few concerns that relate to points #1 and #2 from the previous review:

- The authors have benchmarked the performance of their method against a previously published method HapCut2. While their focus is on specifically long-range switch errors, there are several standard metrics used for assessing haplotyping methods: switch error rate (sometimes sub-classified into short and long switch error rates), hamming error rate (that better reflects global haplotype accuracy), phasing completeness (% of variants that are in the final haplotypes). Since this is a method paper, it would be appropriate to show a head to head comparison with previous methods(s) on these metrics in the main text. The results for Hapcut2 are in a supplementary table and seem to indicate that it has higher completeness that the method presented in this paper. Also, it would be useful to clarify if the high error rate for some chromosomes is due to incorrect phasing across the centromere.

- The authors should define long-range switch errors since this is key for their method.

- In additional data 4 (RPE1), PacBioCCS_15X, the number of phased variants in 'all variants' is lower than when using only 'high-quality variants'. Perhaps the labels have been switched.

- To enable reproducibility, the authors should provide details on how other methods such as Hapcut and their own method were run on the data, i.e. the parameters/commands used

**Reviewer 2**

I find the authors have addressed most of my minor concerns and improved their manuscript accordingly. Below are my responses to the authors upon their revision.

My only major suggestion was to compare parental k-mers directly to the artificially reconstructed chromosomes from phased variants produced by mLinker. The authors limited their analysis on phased SNV sites and presented specificity. However, it was unclear what the Additional Data Table 3 is intended for with no legends provided. What does the numbers mean? No detailed descriptions were found either in the main text. The authors’ response is also running straight into conclusion, which is difficult to follow without better description regarding Additional Data Table 3. I urge the authors to add better descriptions, to the main text or the Additional Data Tables. I would defer my take on this analysis until I have a better understanding.

Additional minor comments below.

1. Provide missing legends to the Additional Data Tables.

2. Fig. 6: One of my earlier comments asked for adding arrows to Fig. 6A. I see arrows were added to panel C (right), which much better improves the understanding of the rearranged chromosomes of K-562. I’d suggest adding arrows to Fig. 6A as well, to the chromosome bars below the dotplots for RPE-1.

3. Fig. S5: cis and trans are more widely used terms for HiC interactions. I’d suggest replacing “major” and “minor” in panel B, D to “cis” and “trans”.

4. In the discussion, page 10, specifically describe the PacBio sequencing being used. I assume this being HiFi. Also, in the Methods.

5. Page 13, correct grammar: “we can simply four types of linkage between geno-types …”. Was this supposed to be “simplify”?

6. Page 17, remove the “n” from “haven” in the last sentence, right above the equation 21

**Authors Response**

**Point-by-point responses to the reviewers’ comments:**

*General remarks about changes:*

*The most important addition in this revision are two subsections in Method ("Haplotype inference from linked-reads/long-read and Hi-C sequencing data by HapCUT2" and "Comparison of phased variant genotypes with parent-specific k-mer’s"). The first section addresses the comparison between our method (mLinker) and a previous published method (HapCUT2) on the same data as used in this study; the second section is devoted to the k-mer based benchmarking of haplotype inference suggested by reviewer 2. Each subsection is accompanied by a new supplementary figure: Fig.S7 for the HapCUT2 comparison shows large switching blocks in the HapCUT2 solution generated using unfiltered variants; an illustration figure within the k-mer comparison section explains different patterns of k-mer matching.*

*Besides text revisions for improving readability (highlighted in red), we have made the following changes related to results added in the previous revision.*

*There are three changes related to Additional Data Table 5: HapCUT2 Benchmark. Two changes are related to benchmarking the HapCUT2 solution of the RPE-1 data. (1) In assessing the accuracy of the HapCUT2 solution derived using all variants, we now use all variants in the truth data (monosomy sequencing) for comparison. This maintains consistency of comparison with the metric reported in Table 3, first row and Additional Data Table 4 ("No filter") for the mLinker solution. (2) In assessing the accuracy of HapCUT2 solution derived from high-quality variants, we now use high-quality variants derived by mLinker (previously this was derived from the single-cell monosomy data) to ensure high variant density on Chr.19. (3) We corrected an error in generating the HapCUT2 solution of Chr.21 from the NA12878 data, which restores the accuracy of the HapCUT2 solution. These new changes reinforce the conclusion that the accuracy of HapCUT2 solution depends on high-quality variants as input.*

*We added detailed information about the PacBio data (with key metrics also updated in Table 1) and detailed legends of the Additional Data Tables. The depth of the PacBio CCS data is 11x instead of 15x as in the previous version. This has been updated throughout the manuscript (text and figures).*

*In Table 2, the benchmarks of phased indel genotypes saw minor changes as we now restrict the comparison to variants with exactly matching reference and alternate genotypes. In the previous version, the comparison was done on variants shared between the benchmark and truth data solely based on their positions. The new comparison is more specific and shows modest improvement in indel phasing accuracy.*

Response to reviewers' questions/comments

Reviewer #1: The authors have revised the manuscript significantly in response to the previous comments. They have highlighted that the main advance of their method is the ability to generate whole-chromosome haplotypes with no long-range switching errors and to do so even when the input set of variants has errors. Indeed, their method is designed to carefully build chromosome-scale haplotypes using long haplotype blocks and Hi-C data in order to avoid long-range errors. However, the abstract is very generic and does not reflect these specific advances of their method. Rewriting the abstract will help bring out the specific contributions of the method described in this paper.

*We thank the reviewer for his/her enthusiasm about our work but wish to point out that "the main advance" of our method as mentioned by the reviewer, "the ability to generate whole-chromosome haplotypes with no long-range switching errors," was clearly articulated in the original submission. Both the title and the abstract clearly state that the main advancement is to determine "complete chromosomal haplotypes" from bulk sequencing data with high accuracy and completeness. The reviewer appeared to suggest that the advantages of our method are restricted to: (1) "carefully build chromosome-scale haplotypes to avoid long-range errors;" (2) "achieve accurate haplotype inference when the input set of variants contains false variants." These features are essential to the determination of complete chromosomal haplotypes, i.e., without long-range errors and robust against false variants. We note that the determination of complete chromosomal haplotypes requires both identification of heterozygous variants and correctly resolving their haplotype phase on parental chromosomes. Therefore, being able to identify heterozygous variants at sufficient accuracy that will enable accurate haplotype inference is an important component of the problem addressed in this paper. Based on the reviewer's comments, we think that the reviewer already recognizes and acknowledges the methodological advancements of our method but perhaps has not fully appreciated the essentiality of these features to the main problem being addressed here ("reliable determination of complete chromosomal haplotypes"). Given that there has been no computational method specifically designed and validated for addressing this problem, we do not think any significant revision or editing of the abstract is necessary. The only change we made is to add "reliably" to "determine complete whole-chromosome haplotypes..." We think this change adequately addresses the reviewer's comment.*

In addition, I have a few concerns that relate to points #1 and #2 from the previous review:

- The authors have benchmarked the performance of their method against a previously published method HapCut2. While their focus is on specifically long-range switch errors, there are several standard metrics used for assessing haplotyping methods: switch error rate (sometimes sub-classified into short and long switch error rates), hamming error rate (that better reflects global haplotype accuracy), phasing completeness (% of variants that are in the final haplotypes). Since this is a method paper, it would be appropriate to show a head to head comparison with previous methods(s) on these metrics in the main text. The results for Hapcut2 are in a supplementary table and seem to indicate that it has higher completeness that the method presented in this paper. Also, it would be useful to clarify if the high error rate for some chromosomes is due to incorrect phasing across the centromere.

*The reviewer raises various points here that we will address separately.*

*1. Definitions of phasing accuracy and completeness*

*Throughout the study, we have used the same metric of phasing accuracy (equivalent to hamming error rate as the reviewer mentioned) and phasing completeness (same as mentioned by the reviewer). Hamming distance is a term borrowed from computer science that may not be familiar to computational or general biologists, so we avoided using this specific term but defined phasing accuracy using simple explanations as follows.*

*Phasing accuracy is first defined in the 3rd paragraph in "Computational inference of parental haplotypes in diploid genomes" as "the fraction of genotypes consistent with the major haplotype assignment." Right after this definition, we immediately comment that this metric "is very sensitive to long-range switching errors that cause a large fraction of genotypes to be assigned to the minor haplotype,", which is further illustrated in Fig.4. All of these were in the original submission.*

*With regard to the accuracy and completeness of whole-chromosome haplotype inference, we provided all the numbers in Table 2, Table 3, Table S2 and Table S3 in the original submission, with detailed explanations of column headers in the table footnotes. We further added new metrics in Additional Data Table 3 and 4 in the previous revision. In the current revision, we also added detailed legends of these data tables although they should be easily interpretable based on the column headers in Table S2 and S3.*

*We therefore believe the benchmarking metrics have been well defined and there is no need for further clarification.*

*2. Short and long switching errors.*

*Short switching errors have been used to refer to single incorrectly phased genotypes in contrast to long switching errors referring to a number of adjacent genotypes whos haplotype phase is opposite to the rest of the chromosome. The definition of "long switching errors" can be arbitrary. For example, are two adjacent incorrectly phased genotypes representing a single long-switching error, or two short switching errors? What if the two variants are separated by a centromere gap? The definition of short switching errors can also vary depending on how false variants are handled. Due to these caveats, the interpretation of these metrics should generally be taken with caution.*

*We further argue that the separation of short and long switching errors is not informative about global phasing accuracy. For example, if the p-arm haplotype and the q-arm haplotype are individually phased correctly but correspond to opposite parental chromosomes, one can still claim high phasing accuracy based on the low frequency of "short" or "long" switching errors. (If there are a total of 10,000 variants, making one such long-range switching error will give a long switching error rate of 10-4.) However, based on the simple definition of global phasing accuracy as we use in this manuscript, haplotype solution with even one such long-range switching error is unacceptable (accuracy drops to 50-60%). In general, the error rate based on a direct comparison of the inferred and the truth haplotype reflects the combination of short and long switching errors and is the most straightforward measure of phasing accuracy. That we consistently achieve 99% accuracy based on this metric already establishes the reliable performance of our method. We therefore do not think separate metrics of short and long switching errors are relevant for our study. However, we did revise the text to be more specific about what we consider as having long-range errors. See the response to the reviewer's second point below.*

*3. The necessity to include head-to-head comparison against previous methods.*

*As this is a Method paper but not a Software paper, our priority here is to demonstrate the performance of our method using real data and the utility of our method to derive biological insight, rather than a side-by-side comparison demonstrating superiority of our method against previous software implementations. The presentation of apparent improvement in artificial benchmark metrics does not guarantee novelty or superiority given that many methods are developed with different priorities, optimized for different applications, and may require parameter tuning for different types of input data. We therefore do not think such benchmarks are informative to a general reader or deserve extensive discussion in the main text.*

*To highlight the key features of our method that are absent in previous studies, we did compare the performance of our method (mLinker) to a previously published method (HapCUT2) and include these results in Additional Data Table 5 with a detailed discussion in a new section in Method (Haplotype inference from linked-reads/long-read and Hi-C sequencing data by HapCUT2) for readers who may be interested in knowing the differences between them. As our algorithm is completely different from HapCUT2, we think readers interested in the technical details will appreciate the differences after a thorough reading of Method.*

*4. Higher completeness and switching errors in the HapCUT2 solution*

*As the newly included Fig.S7 shows, chromosomes with significant lower phasing accuracy (<90%) in the HapCUT2 solution indeed contain large switching blocks with a few switching errors occurring at the centromere. By contrast, mLinker solutions never show such switching errors. As mentioned above, these errors will not cause a significant increase in either short- or long-switching errors, but clearly destroy whole-chromosome scale phasing accuracy. These examples also demonstrate that the metrics of short- and long-range switching errors are not informative for the problem of whole-chromosome haplotype inference.*

*With regard to the higher apparent completeness of the HapCUT2 solution mentioned by the reviewer, the lower number of phased variants in the mLinker solution is due to filtration and exclusion of false variants in the final haplotype solution. As HapCUT2 does not have this feature, it attempts to phase all variants and its accuracy is sensitive to the quality of input variants. The poor accuracy in the HapCUT2 solution when all variants are used relative to the decent accuracy of HapCUT2 solution derived only from high-quality variants clearly demonstrates the necessity of high-quality input variants. These points are now discussed in great detail in the new subsection "Haplotype inference from linked-reads/long-read and Hi-C sequencing data by HapCUT2"*

- The authors should define long-range switch errors since this is key for their method.

*We think the examples in Fig.4 and Fig.S7 give good intuitive examples of what we consider as long-range switching errors. To define these quantitatively, however, requires the introduction of operational thresholds (e.g., how big a switched block is a long switching error) that are often subjective. To eliminate ambiguity associated with the mentioning of "long-range switching errors," we have revised the text to use phasing accuracy (first defined in the 3rd paragraph in "Computational inference of parental haplotypes in diploid genomes") instead of "long-range accuracy":*

*4th paragraph in "Computational inference of parental haplotypes in diploid genomes": "have no apparent intra-block switching (<99% accuracy)";*

*3rd paragraph in "Benchmark of the haplotype solution": "No chromosome in either sample shows <95% accuracy, suggesting that the combined error rate of mismatch (short switching) and block-switching errors is less than 5%."*

*1st paragraph in "Haplotype inference with down-sampled data" : "...large switching blocks (>10% of the entire chromosome)"*

*4th paragraph in "Discussion": "show consistent global phasing accuracy (>95%) relative to a single parental haplotype."*

- In additional data 4 (RPE1), PacBioCCS_15X, the number of phased variants in 'all variants' is lower than when using only 'high-quality variants'. Perhaps the labels have been switched.

*The labelling is correct and the difference between the two solutions is due to different input variants. This was explained in the section "Haplotype inference with down-sampled data" in the previous revision:*

*"When the complete variant callset was used as input, we needed to choose a conservative switching cutoff (∆E = 250) to avoid switching errors due to false variants... When we used high-quality variants (determined by the linkage filter from linked reads) as input, we could lower the switching cutoff to ∆E = 5 and still preserve intra-block accuracy..."*

*The seemingly counter-intuitive result of having fewer phased variants when using more as input is due to our algorithm being stringent about true haplotype linkage: the presence of false variants creates ambiguous linkage that contaminates true haplotype linkage and therefore results in both lower phasing accuracy and completeness.*

*In the current revision, we performed additional variant recovery that increased the number of phased genotypes. The new numbers are updated both in the text and in Additional Data Table 4.*

- To enable reproducibility, the authors should provide details on how other methods such as Hapcut and their own method were run on the data, i.e. the parameters/commands used

*The exact commands for our method (mLinker), including all customizable and built-in parameters, were described in great detail in the Method section in the original submission with additional examples in the github repository. In the current revision, we also provided all the commands used for generating the HapCUT2 results.*

Reviewer #2: I find the authors have addressed most of my minor concerns and improved their manuscript accordingly. Below are my responses to the authors upon their revision.

My only major suggestion was to compare parental k-mers directly to the artificially reconstructed chromosomes from phased variants produced by mLinker. The authors limited their analysis on phased SNV sites and presented specificity. However, it was unclear what the Additional Data Table 3 is intended for with no legends provided. What does the numbers mean? No detailed descriptions were found either in the main text. The authors’ response is also running straight into conclusion, which is difficult to follow without better description regarding Additional Data Table 3. I urge the authors to add better descriptions, to the main text or the Additional Data Tables. I would defer my take on this analysis until I have a better understanding.

*We now added a full subsection in Method ("Comparison of phased variant genotypes with parent-specific k-mer’s") that hopefully has addressed the reviewer's question.*

Additional minor comments below.

1. Provide missing legends to the Additional Data Tables.

*We have added detailed legends to Additional Data Tables 1-4, with further discussion and explanation of results in Additional Data Table 3 and 5 in two new subsections in Method.*

2. Fig. 6: One of my earlier comments asked for adding arrows to Fig. 6A. I see arrows were added to panel C (right), which much better improves the understanding of the rearranged chromosomes of K-562. I’d suggest adding arrows to Fig. 6A as well, to the chromosome bars below the dotplots for RPE-1.

*We have added arrows to Fig.6A according to the same definition as in Fig.6C.*

3. Fig. S5: cis and trans are more widely used terms for HiC interactions. I’d suggest replacing “major” and “minor” in panel B, D to “cis” and “trans”.

*For Hi-C interactions, "cis" and "trans" refer to intra- and inter-molecular Hi-C links determined using true parental haplotypes. As we use dominant Hi-C links to infer the parental haplotype phase, the "major" and "minor" links refer to "cis" and "trans" Hi-C links according to the inferred haplotype. The equivalence of "major" and "cis" (and "minor" and "trans") Hi-C links is preconditioned on the correct inference of p- and q-arm haplotypes. If for example, either the p- or the q-arm haplotype was not fully accurate, then some of the "major" Hi-C links are in fact inter-chromosomal and some of the "minor" links are intra-chromosomal. We feel that this distinction is subtle but important, so have kept both annotations in the figure and its legend.*

4. In the discussion, page 10, specifically describe the PacBio sequencing being used. I assume this being HiFi. Also, in the Methods.

*A new subsection in Method ("PacBio Circular Consensus Sequencing data of RPE-1 cells") is added and additional details of the data (including its SRA ID) are provided in Table 1. We updated the mean sequencing depth of the PacBio data to 11x instead of 15x as originally stated.*

5. Page 13, correct grammar: “we can simply four types of linkage between geno-types …”. Was this supposed to be “simplify”?

6. Page 17, remove the “n” from “haven” in the last sentence, right above the equation 21

*We thank the reviewer for his/her careful reading of the manuscript and apologize for occasional typos.*

**Third round of review**

**Reviewer 1**

The response to the previous comments indicate that the authors have a different perspective about benchmarking results that should be included in a Methods paper. The authors' revisions are acceptable to me, I have no further comments.
